# Supplementary material for: Copper(II) Complexes with 4-Substituted 2,6-Bis(thiazol-2-yl)pyridines—An Overview of Structural–Optical Relationships
Source: Int J Mol Sci. 2025 Dec 9;26(24):11868. doi: 10.3390/ijms262411868 (PMC12733273; doi:10.3390/ijms262411868)

checkCIF/PLATON report

Structure factors have been supplied for datablock(s) cucl2dtpy

THIS REPORT IS FOR GUIDANCE ONLY. IF USED AS PART OF A REVIEW PROCEDURE FOR PUBLICATION, IT SHOULD NOT REPLACE THE EXPERTISE OF AN EXPERIENCED CRYSTALLOGRAPHIC REFEREE.

No syntax errors found. CIF dictionary Interpreting this report

Datablock: cucl2dtpy

```
Bond precision:   C-C = 0.0043 A          Wavelength=0.71073

Cell:             a=8.1922(5)     b=11.1975(6)        c=15.9212(10)
                  alpha=90        beta=104.569(7)     gamma=90
Temperature:      293 K

                 Calculated                  Reported
Volume           1413.53(15)                 1413.52(15)
Space group      P 21/n                      P 1 21/n 1
Hall group       -P 2yn                      -P 2yn
Moiety formula   C11 H7 Cl2 Cu N3 S2, H2 O   C11 H7 Cl2 Cu N3 S2, H2 O
Sum formula      C11 H9 Cl2 Cu N3 O S2       C11 H9 Cl2 Cu N3 O S2
Mr               397.78                      397.77
Dx,g cm-3        1.869                       1.869
Z                4                           4
Mu (mm-1)        2.214                       2.214
F000             796.0                       796.0
F000'            799.60
h,k,lmax         11,15,22                    11,15,20
Nref             3911                        3430
Tmin,Tmax        0.750,0.856                 0.711,1.000
Tmin'            0.750

Correction method= # Reported T Limits: Tmin=0.711 Tmax=1.000
AbsCorr = MULTI-SCAN
```

```
Data completeness= 0.877            Theta(max)= 29.424

R(reflections)= 0.0344( 2679)                          wR2(reflections)=
                                                        0.0819( 3430)
S = 1.066                  Npar= 184
```

The following ALERTS were generated. Each ALERT has the format
test-name_ALERT_alert-type_alert-level.
Click on the hyperlinks for more details of the test.

Alert level C

```
PLAT910_ALERT_3_C Missing FCF Reflection(s) Below Theta(Min)[Deg]=       3.64 Note
                1  1  0,  -1  0  1,   1  0  1,  -1  1  1,   0  1  1,   0  0  2,
                0  1  2,
```

Alert level G

```
PLAT007_ALERT_5_G Number of Unrefined Donor-H Atoms .............          2 Report
              H1A     H1B
PLAT199_ALERT_1_G Reported _cell_measurement_temperature ..... (K)        293 Check
PLAT200_ALERT_1_G Reported   _diffrn_ambient_temperature ..... (K)        293 Check
PLAT794_ALERT_5_G Tentative Bond Valency for Cu1       (II)      .       2.24 Info
PLAT883_ALERT_1_G Absent Datum for _atom_sites_solution_primary ..     Please Do !
PLAT912_ALERT_4_G Missing # of FCF Reflections Above STh/L=  0.600        451 Note
PLAT941_ALERT_3_G Average HKL Measurement Multiplicity ..........         2.5 Low
PLAT952_ALERT_5_G Calculated (ThMax) and CIF-Reported Lmax Differ.          2 Units
PLAT958_ALERT_1_G Calculated (ThMax) and Actual (FCF) Lmax Differ.          2 Units
PLAT969_ALERT_5_G The 'Henn et al.' R-Factor-gap value ..........       2.407 Note
              Predicted wR2: Based on SigI**2  3.40 or SHELX Weight  7.69
PLAT978_ALERT_2_G Number C-C Bonds with Positive Residual Density.          1 Info
```

```
   0 ALERT level A = Most likely a serious problem - resolve or explain
   0 ALERT level B = A potentially serious problem, consider carefully
   1 ALERT level C = Check. Ensure it is not caused by an omission or oversight
  11 ALERT level G = General information/check it is not something unexpected

   4 ALERT type 1 CIF construction/syntax error, inconsistent or missing data
   1 ALERT type 2 Indicator that the structure model may be wrong or deficient
   2 ALERT type 3 Indicator that the structure quality may be low
   1 ALERT type 4 Improvement, methodology, query or suggestion
   4 ALERT type 5 Informative message, check
```

It is advisable to attempt to resolve as many as possible of the alerts in all categories. Often the minor alerts point to easily fixed oversights, errors and omissions in your CIF or refinement strategy, so attention to these fine details can be worthwhile. It is up to the individual to critically assess their own results and, if necessary, seek expert advice.

PLATON version of 26/09/2025; check.def file version of 20/09/2025

duplicate check

No duplication found

Datablock cucl2dtpy - ellipsoid plot

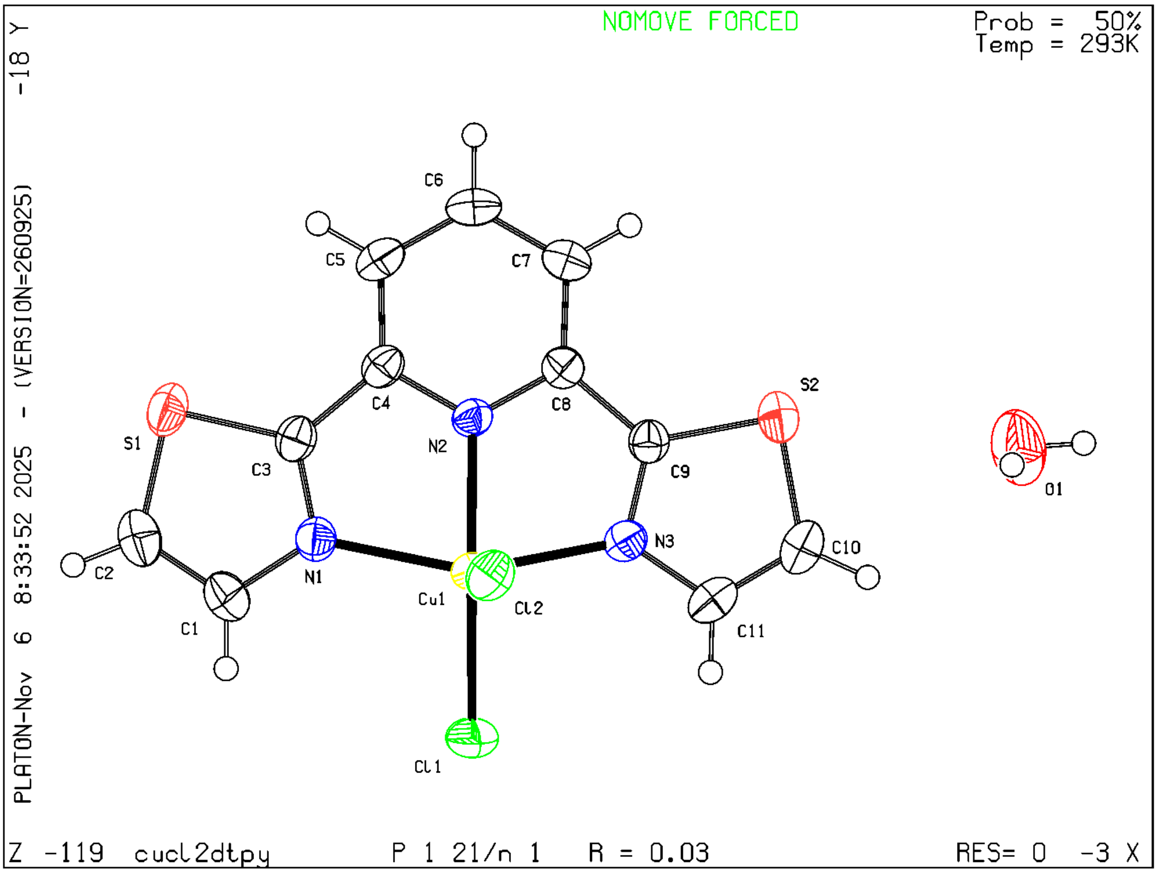

Supplement: Supplementary file 1 [file ijms-26-11868-s001.zip › ESI/checkcif_1.pdf]
