# Supplementary material for: Copper(II) Complexes with 4-Substituted 2,6-Bis(thiazol-2-yl)pyridines—An Overview of Structural–Optical Relationships
Source: Int J Mol Sci. 2025 Dec 9;26(24):11868. doi: 10.3390/ijms262411868 (PMC12733273; doi:10.3390/ijms262411868)

checkCIF/PLATON report

Structure factors have been supplied for datablock(s) cucl2phdtpyb

THIS REPORT IS FOR GUIDANCE ONLY. IF USED AS PART OF A REVIEW PROCEDURE FOR PUBLICATION, IT SHOULD NOT REPLACE THE EXPERTISE OF AN EXPERIENCED CRYSTALLOGRAPHIC REFEREE.

No syntax errors found.   CIF dictionary   Interpreting this report

Datablock: cucl2phdtpyb

Bond precision: C-C = 0.0047 A   Wavelength=0.71073

Cell:	a=18.5235(10)	b=8.4191(4)	c=23.4437(12)
	alpha=90	beta=102.266(5)	gamma=90
Temperature:	293 K		

	Calculated	Reported
Volume	3572.6(3)	3572.6(3)
Space group	P 21/n	P 1 21/n 1
Hall group	-P 2yn	-P 2yn
Moiety formula	C17 H11 Cl2 Cu N3 S2	C17 H11 Cl2 Cu N3 S2
Sum formula	C17 H11 Cl2 Cu N3 S2	C17 H11 Cl2 Cu N3 S2
Mr	455.86	455.85
Dx,g cm-3	1.695	1.695
Z	8	8
Mu (mm-1)	1.760	1.760
F000	1832.0	1832.0
F000’	1839.24	
h,k,lmax	22,10,27	22,10,27
Nref	6312	6293
Tmin,Tmax	0.617,0.824	0.639,1.000
Tmin’	0.605	

Correction method= # Reported T Limits: Tmin=0.639 Tmax=1.000
AbsCorr = MULTI-SCAN

Data completeness= 0.997 Theta(max)= 25.050

R(reflections)= 0.0324(5045) wR2(reflections)= 0.0815(6293)

S = 1.032 Npar= 451

The following ALERTS were generated. Each ALERT has the format
test-name_ALERT_alert-type_alert-level.
Click on the hyperlinks for more details of the test.

Alert level B

```
PLAT910_ALERT_3_B Missing FCF Reflection(s) Below Theta(Min)[Deg]=       3.52 Note
                1  1  0,   2  0  0,   2  1  0,  -3  0  1,  -2  1  1,  -1  0  1,
               -1  1  1,   0  1  1,   1  0  1,   1  1  1,  -2  0  2,  -1  1  2,
                0  0  2,   0  1  2,   1  1  2,   2  0  2,  -1  0  3,   1  0  3,
```

Alert level C

```
PLAT241_ALERT_2_C High   'MainMol' Ueq as Compared to Neighbors of        C31 Check
PLAT242_ALERT_2_C Low    'MainMol' Ueq as Compared to Neighbors of        C29 Check
PLAT331_ALERT_2_C Small Aver Phenyl C-C Dist C29      --C34      .       1.37 Ang.
```

Alert level G

```
PLAT199_ALERT_1_G Reported _cell_measurement_temperature ..... (K)        293 Check
PLAT200_ALERT_1_G Reported   _diffrn_ambient_temperature ..... (K)        293 Check
PLAT794_ALERT_5_G Tentative Bond Valency for Cu1       (II)      .       2.27 Info
PLAT794_ALERT_5_G Tentative Bond Valency for Cu2       (II)      .       2.28 Info
PLAT909_ALERT_3_G Percentage of I>2sig(I) Data at Theta(Max) Still        63% Note
PLAT941_ALERT_3_G Average HKL Measurement Multiplicity ..........         2.4 Low
PLAT961_ALERT_5_G Dataset Contains no Negative Intensities ......     Please Check
PLAT967_ALERT_5_G Note: Two-Theta Cutoff Value in Embedded .res ..       50.1 Degree
PLAT969_ALERT_5_G The 'Henn et al.' R-Factor-gap value ..........       2.459 Note
              Predicted wR2: Based on SigI**2  3.31 or SHELX Weight  7.89
PLAT978_ALERT_2_G Number C-C Bonds with Positive Residual Density.          1 Info
```

---

0 **ALERT level A** = Most likely a serious problem - resolve or explain
1 **ALERT level B** = A potentially serious problem, consider carefully
3 **ALERT level C** = Check. Ensure it is not caused by an omission or oversight
10 **ALERT level G** = General information/check it is not something unexpected

2 ALERT type 1 CIF construction/syntax error, inconsistent or missing data
4 ALERT type 2 Indicator that the structure model may be wrong or deficient
3 ALERT type 3 Indicator that the structure quality may be low
0 ALERT type 4 Improvement, methodology, query or suggestion
5 ALERT type 5 Informative message, check

It is advisable to attempt to resolve as many as possible of the alerts in all categories. Often the minor alerts point to easily fixed oversights, errors and omissions in your CIF or refinement strategy, so attention to these fine details can be worthwhile. It is up to the individual to critically assess their own results and, if necessary, seek expert advice.

PLATON version of 26/09/2025; check.def file version of 20/09/2025

---

duplicate check

A reduced cell check using CCDC's cellCheckCSD service has found that one or more structures in this CIF are similar to those previously published in the CSD or the ICSD.

DATABLOCK: cucl2phdtpyb

- CSD Refcode: PIBWUU

  CCDC

  [Cell Parameters for PIBWUU: 18.5932,8.4641,23.526(90,102.327,90)]

Datablock cucl2phdtpyb - ellipsoid plot

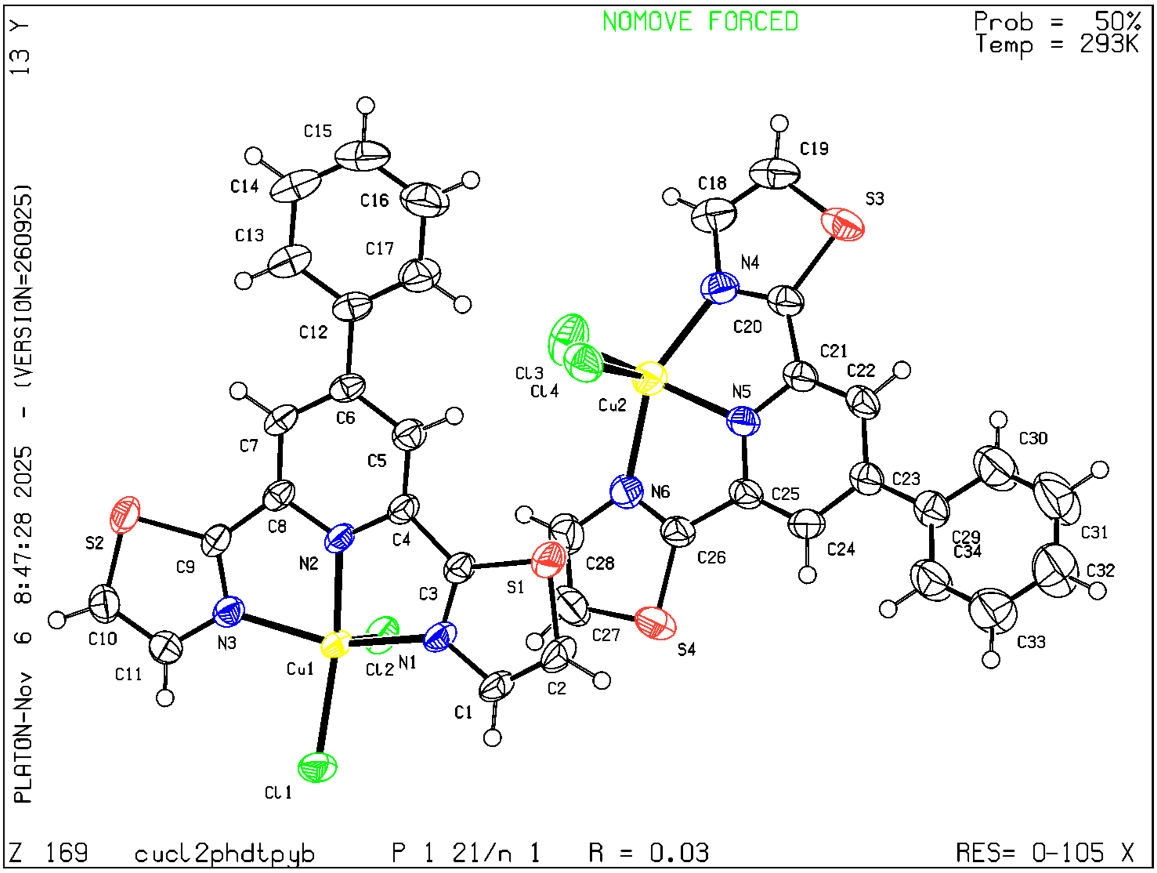

Supplement: Supplementary file 1 [file ijms-26-11868-s001.zip › ESI/checkcif_2.pdf]
