# Supplementary material for: Copper(II) Complexes with 4-Substituted 2,6-Bis(thiazol-2-yl)pyridines—An Overview of Structural–Optical Relationships
Source: Int J Mol Sci. 2025 Dec 9;26(24):11868. doi: 10.3390/ijms262411868 (PMC12733273; doi:10.3390/ijms262411868)

checkCIF/PLATON report

Structure factors have been supplied for datablock(s) cucl2dm16p

THIS REPORT IS FOR GUIDANCE ONLY. IF USED AS PART OF A REVIEW PROCEDURE FOR PUBLICATION, IT SHOULD NOT REPLACE THE EXPERTISE OF AN EXPERIENCED CRYSTALLOGRAPHIC REFEREE.

No syntax errors found. CIF dictionary Interpreting this report

Datablock: cucl2dm16p

Bond precision:	C-C = 0.0043 A		Wavelength=0.71073
Cell:	a=7.5457(7)	b=12.655(1)	c=13.2542(9)
	alpha=77.635(6)	beta=86.523(6)	gamma=80.957(7)
Temperature:	293 K		

	Calculated	Reported
Volume	1220.45(17)	1220.45(17)
Space group	P -1	P -1
Hall group	-P 1	-P 1
Moiety formula	C17 H11 Cl2 Cu N3 O2 S3, 2(C H4 O), H2 O	C17 H11 Cl2 Cu N3 O2 S3, 2(C H4 O), H2 O
Sum formula	C19 H21 Cl2 Cu N3 O5 S3	C19 H21 Cl2 Cu N3 O5 S3
Mr	602.02	602.01
Dx,g cm-3	1.638	1.638
Z	2	2
Mu (mm-1)	1.407	1.407
F000	614.0	614.0
F000’	616.16	
h,k,lmax	10,17,18	9,15,18
Nref	6820	5828
Tmin,Tmax	0.803,0.894	0.709,1.000
Tmin’	0.798	

Correction method= # Reported T Limits: Tmin=0.709 Tmax=1.000
AbsCorr = MULTI-SCAN

```
Data completeness= 0.855            Theta(max)= 29.514

R(reflections)= 0.0447( 4131)                          wR2(reflections)=
                                                       0.1140( 5828)
S = 1.042                    Npar= 324
```

The following ALERTS were generated. Each ALERT has the format
test-name_ALERT_alert-type_alert-level.
Click on the hyperlinks for more details of the test.

Alert level B

```
PLAT910_ALERT_3_B Missing FCF Reflection(s) Below Theta(Min)[Deg]=       3.57 Note
               1  0  0,  -1  1  0,   0  1  0,   1  1  0,   0  2  0,   0 -1  1,
              -1  0  1,   0  0  1,   1  0  1,   0  1  1,   1  1  1,   0  2  1,
               0  0  2,   0  1  2,
```

Alert level C

```
PLAT260_ALERT_2_C Large Average Ueq of Residue Including        O4      0.152 Check
PLAT414_ALERT_2_C Short Intra D-H..H-X       H3       ..H18B     .       1.98 Ang.
                                                    x,y,z  =      1_555 Check
PLAT711_ALERT_1_C BOND    Unknown or Inconsistent Label .........      C14AA Check
                    C14AA       H14A
PLAT711_ALERT_1_C BOND    Unknown or Inconsistent Label .........      C14AA Check
                    C14AA       H14B
PLAT711_ALERT_1_C BOND    Unknown or Inconsistent Label .........      C14AA Check
                    C14AA       C15A
PLAT711_ALERT_1_C BOND    Unknown or Inconsistent Label .........      C15AA Check
                    C15AA       H15A
PLAT711_ALERT_1_C BOND    Unknown or Inconsistent Label .........      C15AA Check
                    C15AA       H15B
PLAT711_ALERT_1_C BOND    Unknown or Inconsistent Label .........      C14BB Check
                    C14BB       H14C
PLAT711_ALERT_1_C BOND    Unknown or Inconsistent Label .........      C14BB Check
                    C14BB       H14D
PLAT711_ALERT_1_C BOND    Unknown or Inconsistent Label .........      C14BB Check
                    C14BB       C15B
PLAT711_ALERT_1_C BOND    Unknown or Inconsistent Label .........      C15BB Check
                    C15BB       H15C
PLAT711_ALERT_1_C BOND    Unknown or Inconsistent Label .........      C15BB Check
                    C15BB       H15D
PLAT712_ALERT_1_C ANGLE   Unknown or Inconsistent Label .........      C15BB Check
                    C15BB       C14BB       O1
PLAT712_ALERT_1_C ANGLE   Unknown or Inconsistent Label .........      C14BB Check
                    C14BB       C15BB       O2
PLAT712_ALERT_1_C ANGLE   Unknown or Inconsistent Label .........      C15AA Check
                    C15AA       C14AA       H14A
PLAT712_ALERT_1_C ANGLE   Unknown or Inconsistent Label .........      H14AA Check
                    H14AA       C14AA       H14B
PLAT712_ALERT_1_C ANGLE   Unknown or Inconsistent Label .........      C15AA Check
                    C15AA       C14AA       H14B
PLAT712_ALERT_1_C ANGLE   Unknown or Inconsistent Label .........      C14AA Check
                    C14AA       C15AA       H15A
```

```
PLAT712_ALERT_1_C ANGLE   Unknown or Inconsistent Label .........      C14AA Check
                    C14AA       C15AA       H15B
PLAT712_ALERT_1_C ANGLE   Unknown or Inconsistent Label .........      H15AA Check
                    H15AA       C15AA       H15B
PLAT712_ALERT_1_C ANGLE   Unknown or Inconsistent Label .........      C15BB Check
                    C15BB       C14BB       H14C
PLAT712_ALERT_1_C ANGLE   Unknown or Inconsistent Label .........      C15BB Check
                    C15BB       C14BB       H14D
PLAT712_ALERT_1_C ANGLE   Unknown or Inconsistent Label .........      H14CB Check
                    H14CB       C14BB       H14D
PLAT712_ALERT_1_C ANGLE   Unknown or Inconsistent Label .........      C14BB Check
                    C14BB       C15BB       H15C
PLAT712_ALERT_1_C ANGLE   Unknown or Inconsistent Label .........      C14BB Check
                    C14BB       C15BB       H15D
PLAT712_ALERT_1_C ANGLE   Unknown or Inconsistent Label .........      H15CB Check
                    H15CB       C15BB       H15D
PLAT712_ALERT_1_C ANGLE   Unknown or Inconsistent Label .........      C14AA Check
                    O1          C14AA       H14A
PLAT712_ALERT_1_C ANGLE   Unknown or Inconsistent Label .........      C14AA Check
                    O1          C14AA       H14B
PLAT712_ALERT_1_C ANGLE   Unknown or Inconsistent Label .........      C14AA Check
                    O1          C14AA       C15A
PLAT712_ALERT_1_C ANGLE   Unknown or Inconsistent Label .........      C14BB Check
                    O1          C14BB       H14C
PLAT712_ALERT_1_C ANGLE   Unknown or Inconsistent Label .........      C14BB Check
                    O1          C14BB       H14D
PLAT712_ALERT_1_C ANGLE   Unknown or Inconsistent Label .........      C15AA Check
                    O2          C15AA       C14A
PLAT712_ALERT_1_C ANGLE   Unknown or Inconsistent Label .........      C15AA Check
                    O2          C15AA       H15A
PLAT712_ALERT_1_C ANGLE   Unknown or Inconsistent Label .........      C15AA Check
                    O2          C15AA       H15B
PLAT712_ALERT_1_C ANGLE   Unknown or Inconsistent Label .........      C15BB Check
                    O2          C15BB       H15C
PLAT712_ALERT_1_C ANGLE   Unknown or Inconsistent Label .........      C15BB Check
                    O2          C15BB       H15D
PLAT713_ALERT_1_C TORSION Unknown or Inconsistent Label .........      C14AA Check
                    C14AA       O1          C13         C12
PLAT713_ALERT_1_C TORSION Unknown or Inconsistent Label .........      C14BB Check
                    C14BB       O1          C13         C12
PLAT713_ALERT_1_C TORSION Unknown or Inconsistent Label .........      C14AA Check
                    C14AA       O1          C13         C16
PLAT713_ALERT_1_C TORSION Unknown or Inconsistent Label .........      C14BB Check
                    C14BB       O1          C13         C16
PLAT713_ALERT_1_C TORSION Unknown or Inconsistent Label .........      C15BB Check
                    C15BB       O2          C16         C13
PLAT713_ALERT_1_C TORSION Unknown or Inconsistent Label .........      C15AA Check
                    C15AA       O2          C16         C13
PLAT713_ALERT_1_C TORSION Unknown or Inconsistent Label .........      C15AA Check
                    C15AA       O2          C16         C17
PLAT713_ALERT_1_C TORSION Unknown or Inconsistent Label .........      C15BB Check
                    C15BB       O2          C16         C17
PLAT713_ALERT_1_C TORSION Unknown or Inconsistent Label .........      C14AA Check
                    O1          C14AA       C15AA       O2
PLAT713_ALERT_1_C TORSION Unknown or Inconsistent Label .........      C14BB Check
                    O1          C14BB       C15BB       O2
PLAT713_ALERT_1_C TORSION Unknown or Inconsistent Label .........      C14AA Check
```

```
                    C13         O1          C14AA       C15AA
PLAT713_ALERT_1_C TORSION Unknown or Inconsistent Label .........      C14BB Check
                    C13         O1          C14BB       C15BB
PLAT713_ALERT_1_C TORSION Unknown or Inconsistent Label .........      C15BB Check
                    C16         O2          C15BB       C14BB
PLAT713_ALERT_1_C TORSION Unknown or Inconsistent Label .........      C15AA Check
                    C16         O2          C15AA       C14AA
PLAT905_ALERT_3_C Negative K value in the Analysis of Variance ...     -0.938 Report
```

Alert level G

```
PLAT007_ALERT_5_G Number of Unrefined Donor-H Atoms .............          4 Report
              H3       H4       H5A      H5B
PLAT199_ALERT_1_G Reported _cell_measurement_temperature ..... (K)        293 Check
PLAT200_ALERT_1_G Reported   _diffrn_ambient_temperature ..... (K)        293 Check
PLAT301_ALERT_3_G Main Residue  Disorder ..............(Resd    1)         7% Note
PLAT790_ALERT_4_G Centre of Gravity not Within Unit-Cell: Resd.  #          3 Note
              C H4 O
PLAT794_ALERT_5_G Tentative Bond Valency for Cu1       (II)      .       2.25 Info
PLAT912_ALERT_4_G Missing # of FCF Reflections Above STh/L=  0.600        761 Note
PLAT941_ALERT_3_G Average HKL Measurement Multiplicity ..........         2.0 Low
PLAT951_ALERT_5_G Calculated (ThMax) and CIF-Reported Kmax Differ           2 Units
PLAT957_ALERT_1_G Calculated (ThMax) and Actual (FCF) Kmax Differ           2 Units
PLAT969_ALERT_5_G The 'Henn et al.' R-Factor-gap value ..........       2.662 Note
              Predicted wR2: Based on SigI**2  4.28 or SHELX Weight 10.93
PLAT978_ALERT_2_G Number C-C Bonds with Positive Residual Density.          1 Info
```

```
   0 ALERT level A = Most likely a serious problem - resolve or explain
   1 ALERT level B = A potentially serious problem, consider carefully
  51 ALERT level C = Check. Ensure it is not caused by an omission or oversight
  12 ALERT level G = General information/check it is not something unexpected

  51 ALERT type 1 CIF construction/syntax error, inconsistent or missing data
   3 ALERT type 2 Indicator that the structure model may be wrong or deficient
   4 ALERT type 3 Indicator that the structure quality may be low
   2 ALERT type 4 Improvement, methodology, query or suggestion
   4 ALERT type 5 Informative message, check
```

It is advisable to attempt to resolve as many as possible of the alerts in all categories. Often the minor alerts point to easily fixed oversights, errors and omissions in your CIF or refinement strategy, so attention to these fine details can be worthwhile. It is up to the individual to critically assess their own results and, if necessary, seek expert advice.

PLATON version of 26/09/2025; check.def file version of 20/09/2025

duplicate check

No duplication found

Datablock cucl2dm16p - ellipsoid plot

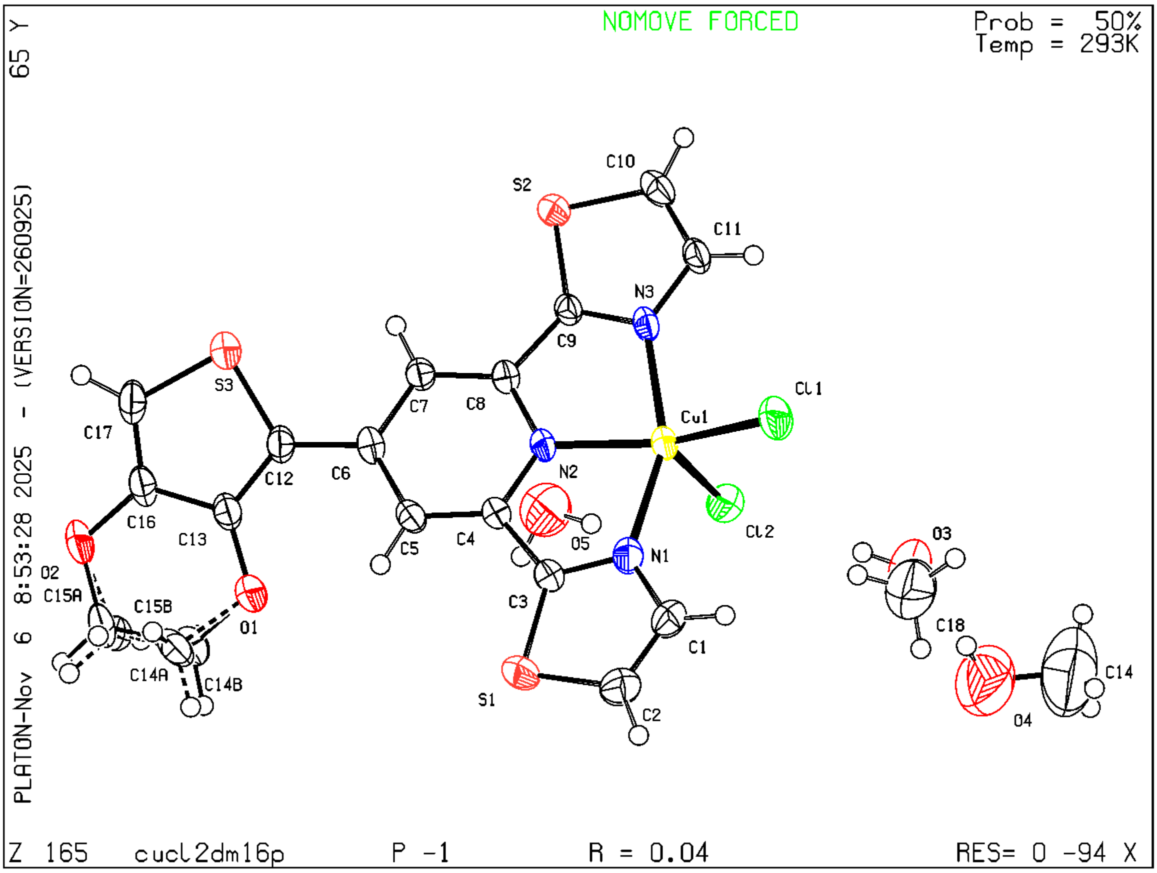

Supplement: Supplementary file 1 [file ijms-26-11868-s001.zip › ESI/checkcif_4.pdf]
