# Supplementary material for: Copper(II) Complexes with 4-Substituted 2,6-Bis(thiazol-2-yl)pyridines—An Overview of Structural–Optical Relationships
Source: Int J Mol Sci. 2025 Dec 9;26(24):11868. doi: 10.3390/ijms262411868 (PMC12733273; doi:10.3390/ijms262411868)

checkCIF/PLATON report

Structure factors have been supplied for datablock(s) cucl2ewm27cz

THIS REPORT IS FOR GUIDANCE ONLY. IF USED AS PART OF A REVIEW PROCEDURE FOR PUBLICATION, IT SHOULD NOT REPLACE THE EXPERTISE OF AN EXPERIENCED CRYSTALLOGRAPHIC REFEREE.

No syntax errors found. CIF dictionary Interpreting this report

Datablock: cucl2ewm27cz

```
Bond precision:    C-C = 0.0047 A          Wavelength=0.71073

Cell:              a=9.7299(4)      b=15.0039(6)        c=15.6869(9)
                   alpha=90         beta=98.481(5)      gamma=90
Temperature:       293 K
```

	Calculated	Reported
Volume	2265.03(19)	2265.03(19)
Space group	P 21/c	P 1 21/c 1
Hall group	-P 2ybc	-P 2ybc
Moiety formula	C19 H11 Cl2 Cu N3 S4, 2(H2 O)	C19 H11 Cl2 Cu N3 S4, 2(H2 O)
Sum formula	C19 H15 Cl2 Cu N3 O2 S4	C19 H15 Cl2 Cu N3 O2 S4
Mr	580.03	580.02
Dx,g cm-3	1.701	1.701
Z	4	4
Mu (mm-1)	1.592	1.592
F000	1172.0	1172.0
F000'	1176.71	
h,k,lmax	13,20,21	13,20,21
Nref	6277	5361
Tmin,Tmax	0.826,0.909	0.752,1.000
Tmin'	0.826	

Correction method= # Reported T Limits: Tmin=0.752 Tmax=1.000
AbsCorr = MULTI-SCAN

Data completeness= 0.854 Theta(max)= 29.434

R(reflections)= 0.0455(3707) wR2(reflections)= 0.1261(5361)

S = 1.021 Npar= 280

The following ALERTS were generated. Each ALERT has the format
test-name_ALERT_alert-type_alert-level.
Click on the hyperlinks for more details of the test.

Alert level B

```
PLAT420_ALERT_2_B D-H Bond Without Acceptor  O1       --H1A      .     Please Check
```

Alert level C

```
PLAT230_ALERT_2_C Hirshfeld Test Diff for    C17      --C18      .         6.7 s.u.
PLAT260_ALERT_2_C Large Average Ueq of Residue Including        O1      0.123 Check
PLAT910_ALERT_3_C Missing FCF Reflection(s) Below Theta(Min)[Deg]=       3.40 Note
                1  0  0,   1  1  0,   0  2  0,  -1  1  1,   0  1  1,   1  1  1,
                0  2  1,  -1  0  2,   0  0  2,   0  1  2,
PLAT975_ALERT_2_C Check Calcd Resid. Dens.  1.04Ang From O1      .       0.48 eA-3
```

Alert level G

```
PLAT007_ALERT_5_G Number of Unrefined Donor-H Atoms .............          4 Report
              H1A     H1B     H2A     H2B
PLAT199_ALERT_1_G Reported _cell_measurement_temperature ..... (K)        293 Check
PLAT200_ALERT_1_G Reported   _diffrn_ambient_temperature ..... (K)        293 Check
PLAT790_ALERT_4_G Centre of Gravity not Within Unit-Cell: Resd.  #          2 Note
              H2 O
PLAT794_ALERT_5_G Tentative Bond Valency for Cu1       (II)      .       2.19 Info
PLAT883_ALERT_1_G Absent Datum for _atom_sites_solution_primary ..     Please Do !
PLAT912_ALERT_4_G Missing # of FCF Reflections Above STh/L=  0.600        906 Note
PLAT941_ALERT_3_G Average HKL Measurement Multiplicity ..........         2.2 Low
PLAT969_ALERT_5_G The 'Henn et al.' R-Factor-gap value ..........       3.747 Note
              Predicted wR2: Based on SigI**2  3.37 or SHELX Weight 12.34
PLAT978_ALERT_2_G Number C-C Bonds with Positive Residual Density.          2 Info
```

---

0 **ALERT level A** = Most likely a serious problem - resolve or explain
1 **ALERT level B** = A potentially serious problem, consider carefully
4 **ALERT level C** = Check. Ensure it is not caused by an omission or oversight
10 **ALERT level G** = General information/check it is not something unexpected

3 ALERT type 1 CIF construction/syntax error, inconsistent or missing data
5 ALERT type 2 Indicator that the structure model may be wrong or deficient
2 ALERT type 3 Indicator that the structure quality may be low
2 ALERT type 4 Improvement, methodology, query or suggestion
3 ALERT type 5 Informative message, check

It is advisable to attempt to resolve as many as possible of the alerts in all categories. Often the minor alerts point to easily fixed oversights, errors and omissions in your CIF or refinement strategy, so attention to these fine details can be worthwhile. It is up to the individual to critically assess their own results and, if necessary, seek expert advice.

PLATON version of 26/09/2025; check.def file version of 20/09/2025

---

duplicate check

No duplication found

Datablock cucl2ewm27cz - ellipsoid plot

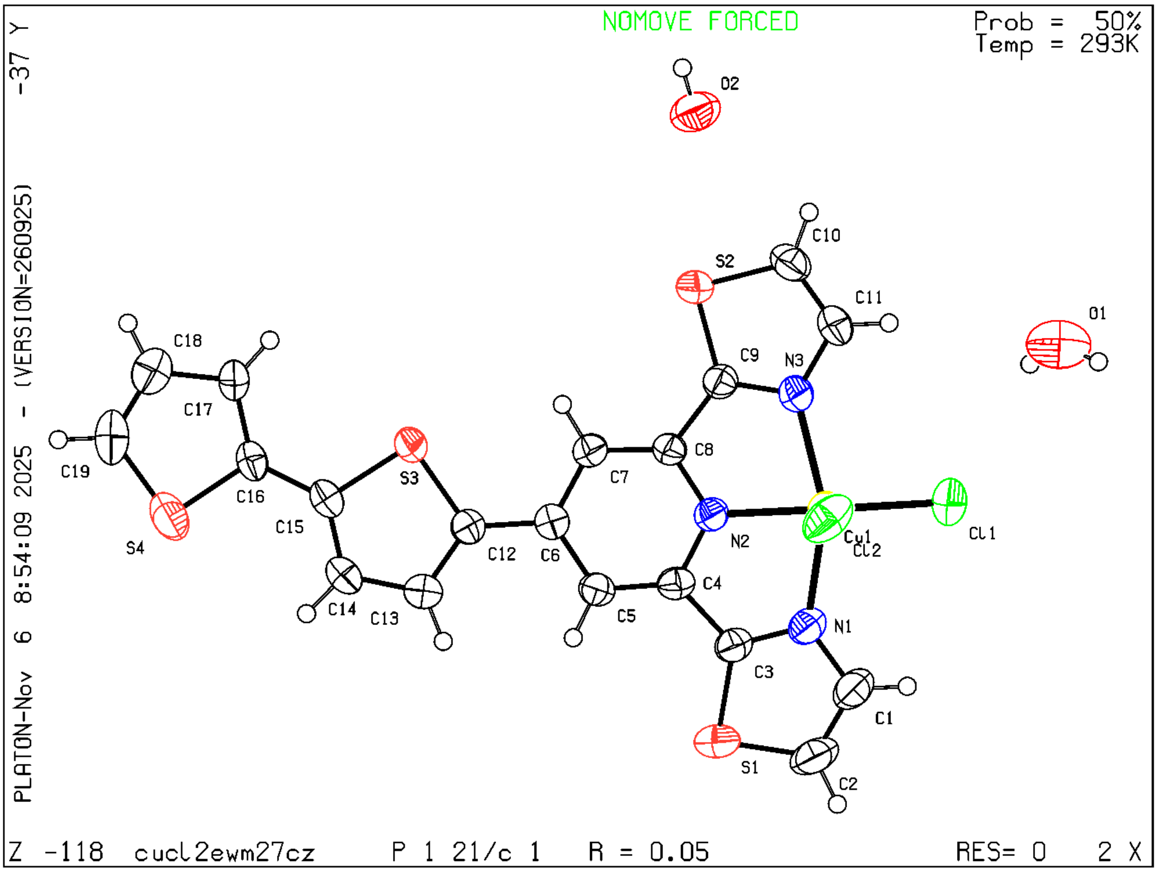

Supplement: Supplementary file 1 [file ijms-26-11868-s001.zip › ESI/checkcif_5.pdf]
