# Supplementary material for: Copper(II) Complexes with 4-Substituted 2,6-Bis(thiazol-2-yl)pyridines—An Overview of Structural–Optical Relationships
Source: Int J Mol Sci. 2025 Dec 9;26(24):11868. doi: 10.3390/ijms262411868 (PMC12733273; doi:10.3390/ijms262411868)

checkCIF/PLATON report

Structure factors have been supplied for datablock(s) cucl2ewm29s

THIS REPORT IS FOR GUIDANCE ONLY. IF USED AS PART OF A REVIEW PROCEDURE FOR PUBLICATION, IT SHOULD NOT REPLACE THE EXPERTISE OF AN EXPERIENCED CRYSTALLOGRAPHIC REFEREE.

No syntax errors found. CIF dictionary Interpreting this report

Datablock: cucl2ewm29s

Bond precision: C-C = 0.0058 A Wavelength=0.71073

Cell:	a=8.0883(5)	b=10.1003(6)	c=12.0986(7)
	alpha=65.536(5)	beta=86.260(5)	gamma=89.456(5)
Temperature:	293 K		

	Calculated	Reported
Volume	897.57(10)	897.57(10)
Space group	P -1	P -1
Hall group	-P 1	-P 1
Moiety formula	C17 H11 Cl2 Cu N3 O S2	C17 H11 Cl2 Cu N3 O S2
Sum formula	C17 H11 Cl2 Cu N3 O S2	C17 H11 Cl2 Cu N3 O S2
Mr	471.86	471.85
Dx,g cm-3	1.746	1.746
Z	2	2
Mu (mm-1)	1.759	1.759
F000	474.0	474.0
F000'	475.83	
h,k,lmax	11,13,16	11,13,15
Nref	4969	4201
Tmin,Tmax	0.810,0.932	0.447,1.000
Tmin'	0.716	

Correction method= # Reported T Limits: Tmin=0.447 Tmax=1.000
AbsCorr = MULTI-SCAN

```
Data completeness= 0.845          Theta(max)= 29.441

R(reflections)= 0.0472( 3093)                    wR2(reflections)=
                                                  0.1245( 4201)
S = 1.108                    Npar= 235
```

The following ALERTS were generated. Each ALERT has the format
test-name_ALERT_alert-type_alert-level.
Click on the hyperlinks for more details of the test.

Alert level C

```
PLAT910_ALERT_3_C Missing FCF Reflection(s) Below Theta(Min)[Deg]=       3.43 Note
                1  0  0,  -1  1  0,   0  1  0,   1  1  0,  -1  0  1,   0  0  1,
                1  0  1,   0  1  1,   1  1  1,
PLAT934_ALERT_3_C Number of (Iobs-Icalc)/Sigma(W) > 10 Outliers ..          1 Check
               -2  3  1,
```

Alert level G

```
PLAT154_ALERT_1_G The s.u.'s on the Cell Angles are Equal ..(Note)      0.005 Degree
PLAT199_ALERT_1_G Reported _cell_measurement_temperature ..... (K)        293 Check
PLAT200_ALERT_1_G Reported   _diffrn_ambient_temperature ..... (K)        293 Check
PLAT398_ALERT_2_G Deviating  C-O-C   Angle From 120 for O1       .      106.4 Degree
PLAT794_ALERT_5_G Tentative Bond Valency for Cu1       (II)      .       2.24 Info
PLAT883_ALERT_1_G Absent Datum for _atom_sites_solution_primary ..     Please Do !
PLAT912_ALERT_4_G Missing # of FCF Reflections Above STh/L=  0.600        721 Note
PLAT941_ALERT_3_G Average HKL Measurement Multiplicity ..........         2.0 Low
PLAT969_ALERT_5_G The 'Henn et al.' R-Factor-gap value ..........      3.221 Note
              Predicted wR2: Based on SigI**2  3.87 or SHELX Weight 11.24
PLAT978_ALERT_2_G Number C-C Bonds with Positive Residual Density.          1 Info
```

```
   0 ALERT level A = Most likely a serious problem - resolve or explain
   0 ALERT level B = A potentially serious problem, consider carefully
   2 ALERT level C = Check. Ensure it is not caused by an omission or oversight
  10 ALERT level G = General information/check it is not something unexpected

   4 ALERT type 1 CIF construction/syntax error, inconsistent or missing data
   2 ALERT type 2 Indicator that the structure model may be wrong or deficient
   3 ALERT type 3 Indicator that the structure quality may be low
   1 ALERT type 4 Improvement, methodology, query or suggestion
   2 ALERT type 5 Informative message, check
```

It is advisable to attempt to resolve as many as possible of the alerts in all categories. Often the minor alerts point to easily fixed oversights, errors and omissions in your CIF or refinement strategy, so attention to these fine details can be worthwhile. It is up to the individual to critically assess their own results and, if necessary, seek expert advice.

PLATON version of 26/09/2025; check.def file version of 20/09/2025

duplicate check

No duplication found

Datablock cucl2ewm29s - ellipsoid plot

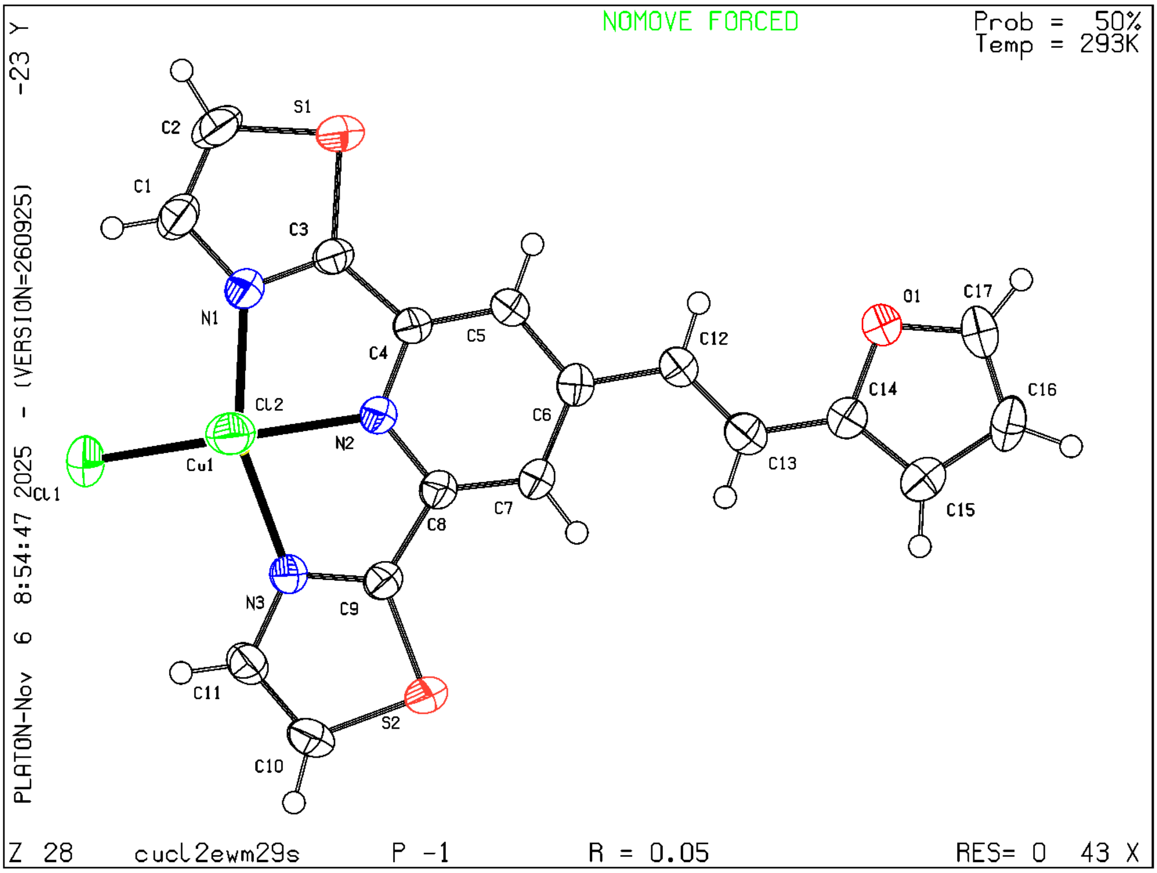

Supplement: Supplementary file 1 [file ijms-26-11868-s001.zip › ESI/checkcif_6.pdf]
