# Supplementary material for: Copper(II) Complexes with 4-Substituted 2,6-Bis(thiazol-2-yl)pyridines—An Overview of Structural–Optical Relationships
Source: Int J Mol Sci. 2025 Dec 9;26(24):11868. doi: 10.3390/ijms262411868 (PMC12733273; doi:10.3390/ijms262411868)

checkCIF/PLATON report

Structure factors have been supplied for datablock(s) cucl2af6

THIS REPORT IS FOR GUIDANCE ONLY. IF USED AS PART OF A REVIEW PROCEDURE FOR PUBLICATION, IT SHOULD NOT REPLACE THE EXPERTISE OF AN EXPERIENCED CRYSTALLOGRAPHIC REFEREE.

No syntax errors found.   CIF dictionary   Interpreting this report

Datablock: cucl2af6

Bond precision: C-C = 0.0052 A   Wavelength=0.71073

Cell:	a=8.0631(5)	b=11.0698(10)	c=12.7644(10)
	alpha=70.692(8)	beta=85.962(6)	gamma=69.087(7)
Temperature:	293 K		

	Calculated	Reported
Volume	1002.93(15)	1002.94(15)
Space group	P -1	P -1
Hall group	-P 1	-P 1
Moiety formula	C18 H10 Cl2 Cu N4 S2 [+ solvent]	C18 H10 Cl2 Cu N4 S2
Sum formula	C18 H10 Cl2 Cu N4 S2 [+ solvent]	C18 H10 Cl2 Cu N4 S2
Mr	480.87	480.86
Dx,g cm-3	1.592	1.592
Z	2	2
Mu (mm-1)	1.574	1.574
F000	482.0	482.0
F000’	483.81	
h,k,lmax	9,13,15	9,13,15
Nref	3564	3554
Tmin,Tmax	0.854,0.910	0.916,1.000
Tmin’	0.854	

Correction method= # Reported T Limits: Tmin=0.916 Tmax=1.000
AbsCorr = MULTI-SCAN

Data completeness= 0.997 Theta(max)= 25.049

R(reflections)= 0.0394(2960) wR2(reflections)= 0.1024(3554)

S = 1.078 Npar= 244

The following ALERTS were generated. Each ALERT has the format
test-name_ALERT_alert-type_alert-level.
Click on the hyperlinks for more details of the test.

Alert level C

```
PLAT910_ALERT_3_C Missing FCF Reflection(s) Below Theta(Min)[Deg]=       3.35 Note
                1  0  0,   0  1  0,   1  1  0,   0 -1  1,  -1  0  1,   0  0  1,
                1  0  1,   0  1  1,   1  1  1,
PLAT911_ALERT_3_C Missing FCF Refl Between Thmin & STh/L=    0.596          2 Report
               -6  5  8,   0  4 13,
```

Alert level G

```
PLAT199_ALERT_1_G Reported _cell_measurement_temperature ..... (K)        293 Check
PLAT200_ALERT_1_G Reported   _diffrn_ambient_temperature ..... (K)        293 Check
PLAT232_ALERT_2_G Hirshfeld Test Diff (M-X)  Cu1      --Cl2      .        8.7 s.u.
PLAT605_ALERT_4_G Largest Solvent Accessible VOID in the Structure        121 A**3
PLAT794_ALERT_5_G Tentative Bond Valency for Cu1       (II)      .       2.30 Info
PLAT868_ALERT_4_G ALERTS Due to the Use of _smtbx_masks Suppressed          ! Info
PLAT909_ALERT_3_G Percentage of I>2sig(I) Data at Theta(Max) Still        69% Note
PLAT941_ALERT_3_G Average HKL Measurement Multiplicity ..........         2.3 Low
PLAT961_ALERT_5_G Dataset Contains no Negative Intensities ......     Please Check
PLAT967_ALERT_5_G Note: Two-Theta Cutoff Value in Embedded .res ..       50.1 Degree
PLAT969_ALERT_5_G The 'Henn et al.' R-Factor-gap value ..........       1.753 Note
              Predicted wR2: Based on SigI**2  5.84 or SHELX Weight  9.50
PLAT978_ALERT_2_G Number C-C Bonds with Positive Residual Density.          4 Info
```

---

0 **ALERT level A** = Most likely a serious problem - resolve or explain
0 **ALERT level B** = A potentially serious problem, consider carefully
2 **ALERT level C** = Check. Ensure it is not caused by an omission or oversight
12 **ALERT level G** = General information/check it is not something unexpected

2 ALERT type 1 CIF construction/syntax error, inconsistent or missing data
2 ALERT type 2 Indicator that the structure model may be wrong or deficient
4 ALERT type 3 Indicator that the structure quality may be low
2 ALERT type 4 Improvement, methodology, query or suggestion
4 ALERT type 5 Informative message, check

It is advisable to attempt to resolve as many as possible of the alerts in all categories. Often the minor alerts point to easily fixed oversights, errors and omissions in your CIF or refinement strategy, so attention to these fine details can be worthwhile. It is up to the individual to critically assess their own results and, if necessary, seek expert advice.

PLATON version of 26/09/2025; check.def file version of 20/09/2025

---

duplicate check

No duplication found

Datablock cucl2af6 - ellipsoid plot

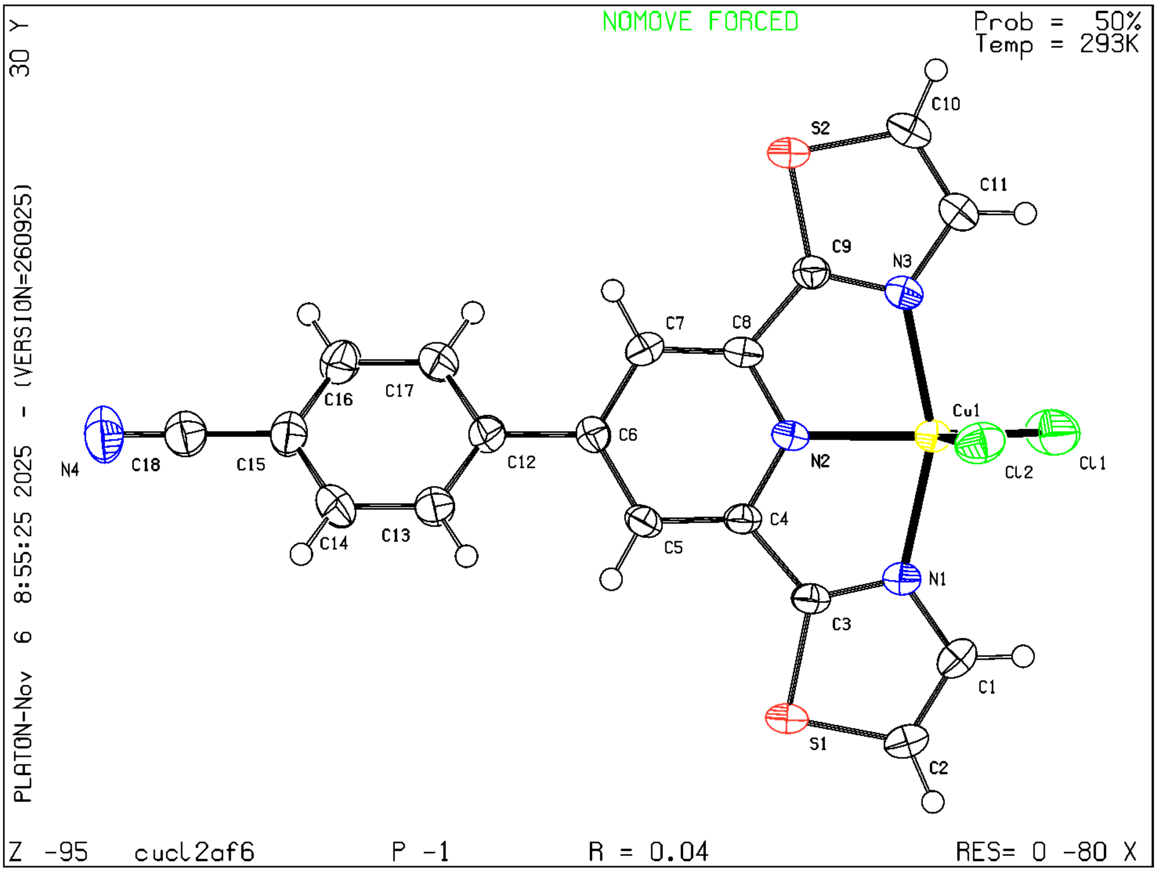

Supplement: Supplementary file 1 [file ijms-26-11868-s001.zip › ESI/checkcif_7.pdf]
