# Supplementary material for: Copper(II) Complexes with 4-Substituted 2,6-Bis(thiazol-2-yl)pyridines—An Overview of Structural–Optical Relationships
Source: Int J Mol Sci. 2025 Dec 9;26(24):11868. doi: 10.3390/ijms262411868 (PMC12733273; doi:10.3390/ijms262411868)

# checkCIF/PLATON report

Structure factors have been supplied for datablock(s) cucl2ewm2

THIS REPORT IS FOR GUIDANCE ONLY. IF USED AS PART OF A REVIEW PROCEDURE FOR PUBLICATION, IT SHOULD NOT REPLACE THE EXPERTISE OF AN EXPERIENCED CRYSTALLOGRAPHIC REFEREE.

No syntax errors found. CIF dictionary Interpreting this report

# Datablock: cucl2ewm2

---

| | | | |
|---|---|---|---|
| Bond precision: | C-C = 0.0054 A | | Wavelength=0.71073 |
| Cell: | a=13.3642(8) | b=7.8994(6) | c=22.1169(10) |
| | alpha=90 | beta=99.082(5) | gamma=90 |
| Temperature: | 293 K | | |

| | Calculated | Reported |
|---|---|---|
| Volume | 2305.6(2) | 2305.6(2) |
| Space group | P 21/c | P 1 21/c 1 |
| Hall group | -P 2ybc | -P 2ybc |
| Moiety formula | C17 H10 Br Cl2 Cu N3 S2 [+ solvent] | C17 H10 Br Cl2 Cu N3 S2 |
| Sum formula | C17 H10 Br Cl2 Cu N3 S2 [+ solvent] | C17 H10 Br Cl2 Cu N3 S2 |
| Mr | 534.75 | 534.75 |
| Dx,g cm-3 | 1.541 | 1.541 |
| Z | 4 | 4 |
| Mu (mm-1) | 3.099 | 3.099 |
| F000 | 1052.0 | 1052.0 |
| F000’ | 1054.54 | |
| h,k,lmax | 18,10,30 | 16,10,30 |
| Nref | 6362 | 5489 |
| Tmin,Tmax | 0.671,0.734 | 0.630,1.000 |
| Tmin’ | 0.567 | |

```
Correction method= # Reported T Limits: Tmin=0.630 Tmax=1.000
AbsCorr = MULTI-SCAN

Data completeness= 0.863            Theta(max)= 29.394

                                                    wR2(reflections)=
R(reflections)= 0.0466( 3824)                       0.1182( 5489)

S = 1.033                Npar= 235
```

---

The following ALERTS were generated. Each ALERT has the format
**test-name_ALERT_alert-type_alert-level**.
Click on the hyperlinks for more details of the test.

---

**Alert level B**

```
PLAT910_ALERT_3_B Missing FCF Reflection(s) Below Theta(Min)[Deg]=       3.41 Note
                 1  0  0,   1  1  0,   2  0  0,  -1  1  1,   0  1  1,   1  1  1,
                -2  0  2,  -1  0  2,   0  0  2,   0  1  2,   1  0  2,
```

---

**Alert level C**

```
PLAT905_ALERT_3_C Negative K value in the Analysis of Variance ...     -0.812 Report
PLAT911_ALERT_3_C Missing FCF Refl Between Thmin & STh/L=    0.600         10 Report
                12  4  2,  12  4  3, -10  4  4, -10  4  5,  12  4  5,  12  4  6,
               -11  4  7, -11  4  8, -12  4 10,   9  3 10,
PLAT934_ALERT_3_C Number of (Iobs-Icalc)/Sigma(W) > 10 Outliers ..          1 Check
                 0  2  4,
```

---

**Alert level G**

```
PLAT199_ALERT_1_G Reported _cell_measurement_temperature ..... (K)        293 Check
PLAT200_ALERT_1_G Reported   _diffrn_ambient_temperature ..... (K)        293 Check
PLAT434_ALERT_2_G Short Inter HL..HL Contact Br1      ..Cl2      .       3.48 Ang.
                                               1-x,2-y,1-z  =     3_676 Check
PLAT605_ALERT_4_G Largest Solvent Accessible VOID in the Structure        288 A**3
PLAT794_ALERT_5_G Tentative Bond Valency for Cu1       (II)      .       2.22 Info
PLAT868_ALERT_4_G ALERTS Due to the Use of _smtbx_masks Suppressed          ! Info
PLAT912_ALERT_4_G Missing # of FCF Reflections Above STh/L=  0.600        748 Note
PLAT941_ALERT_3_G Average HKL Measurement Multiplicity ...........        2.7 Low
PLAT950_ALERT_5_G Calculated (ThMax) and CIF-Reported Hmax Differ           2 Units
PLAT956_ALERT_1_G Calculated (ThMax) and Actual (FCF) Hmax Differ           2 Units
PLAT969_ALERT_5_G The 'Henn et al.' R-Factor-gap value ..........       2.828 Note
              Predicted wR2: Based on SigI**2  4.18 or SHELX Weight 11.45
PLAT978_ALERT_2_G Number C-C Bonds with Positive Residual Density.          2 Info
```

---

0 **ALERT level A** = Most likely a serious problem - resolve or explain
1 **ALERT level B** = A potentially serious problem, consider carefully
3 **ALERT level C** = Check. Ensure it is not caused by an omission or oversight
12 **ALERT level G** = General information/check it is not something unexpected

3 ALERT type 1 CIF construction/syntax error, inconsistent or missing data

```
  2 ALERT type 2 Indicator that the structure model may be wrong or deficient
  5 ALERT type 3 Indicator that the structure quality may be low
  3 ALERT type 4 Improvement, methodology, query or suggestion
  3 ALERT type 5 Informative message, check
```

---

It is advisable to attempt to resolve as many as possible of the alerts in all categories. Often the minor alerts point to easily fixed oversights, errors and omissions in your CIF or refinement strategy, so attention to these fine details can be worthwhile. It is up to the individual to critically assess their own results and, if necessary, seek expert advice.

---

**PLATON version of 26/09/2025; check.def file version of 20/09/2025**

---

# duplicate check

**No duplication found**

**Datablock cucl2ewm2** - ellipsoid plot

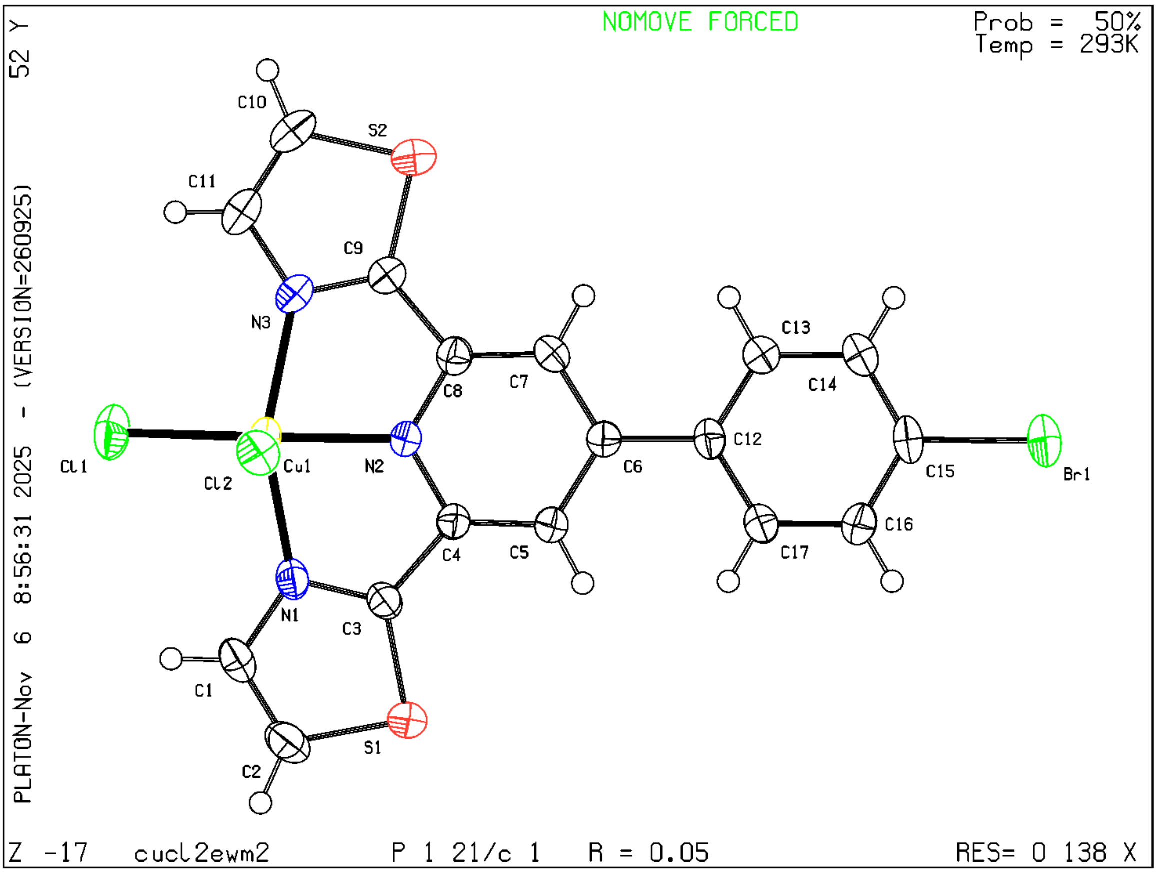

Supplement: Supplementary file 1 [file ijms-26-11868-s001.zip › ESI/checkcif_8.pdf]
