# Supplementary material for: Copper(II) Complexes with 4-Substituted 2,6-Bis(thiazol-2-yl)pyridines—An Overview of Structural–Optical Relationships
Source: Int J Mol Sci. 2025 Dec 9;26(24):11868. doi: 10.3390/ijms262411868 (PMC12733273; doi:10.3390/ijms262411868)

# checkCIF/PLATON report

Structure factors have been supplied for datablock(s) cucl2sktl16a

THIS REPORT IS FOR GUIDANCE ONLY. IF USED AS PART OF A REVIEW PROCEDURE FOR PUBLICATION, IT SHOULD NOT REPLACE THE EXPERTISE OF AN EXPERIENCED CRYSTALLOGRAPHIC REFEREE.

No syntax errors found. CIF dictionary Interpreting this report

## Datablock: cucl2sktl16a

---

```
Bond precision:   C-C = 0.0071 A          Wavelength=0.71073

Cell:            a=8.0603(7)        b=8.9973(7)       c=15.1195(12)
                 alpha=80.145(6)    beta=84.018(7)    gamma=89.634(7)
Temperature:     293 K

                  Calculated                    Reported
Volume            1074.35(15)                   1074.34(15)
Space group       P -1                          P -1
Hall group        -P 1                          -P 1
Moiety formula    C23 H15 Cl2 Cu N3 S2          C23 H15 Cl2 Cu N3 S2
Sum formula       C23 H15 Cl2 Cu N3 S2          C23 H15 Cl2 Cu N3 S2
Mr                531.95                        531.94
Dx,g cm-3         1.644                         1.644
Z                 2                             2
Mu (mm-1)         1.477                         1.477
F000              538.0                         538.0
F000'             539.84
h,k,lmax          9,10,18                       9,10,18
Nref              3817                          3791
Tmin,Tmax         0.853,0.929                   0.750,1.000
Tmin'             0.813

Correction method= # Reported T Limits: Tmin=0.750 Tmax=1.000
AbsCorr = MULTI-SCAN
```

```
Data completeness= 0.993          Theta(max)= 25.049

                                                  wR2(reflections)=
R(reflections)= 0.0552( 2535)                     0.1431( 3791)

S = 0.974                   Npar= 280
```

---

The following ALERTS were generated. Each ALERT has the format
**test-name_ALERT_alert-type_alert-level**.
Click on the hyperlinks for more details of the test.

---

**Alert level C**

```
PLAT341_ALERT_3_C Low Bond Precision on  C-C Bonds ..............    0.00709 Ang.
PLAT790_ALERT_4_C Centre of Gravity not Within Unit-Cell: Resd.  #          1 Note
              C23 H15 Cl2 Cu N3 S2
PLAT906_ALERT_3_C Large K Value in the Analysis of Variance ......      4.299 Check
PLAT910_ALERT_3_C Missing FCF Reflection(s) Below Theta(Min)[Deg]=       3.41 Note
                1  0  0,   0  1  0,   0 -1  1,  -1  0  1,   0  0  1,   1  0  1,
                0  1  1,   0  0  2,   0  1  2,
PLAT911_ALERT_3_C Missing FCF Refl Between Thmin & STh/L=    0.596         17 Report
                0  1  7,  -1  1 12,   1  1 12,  -2  1 13,  -1  1 13,   1  1 13,
                2  0 14,  -4  1 14,   0  1 14,  -3  1 15,  -2  1 15,  -1  1 15,
                3 -1 16,   4 -1 16,   1  0 16,  -1  0 17,   0  0 17,
PLAT934_ALERT_3_C Number of (Iobs-Icalc)/Sigma(W) > 10 Outliers ..          1 Check
               -3  0 13,
```

---

**Alert level G**

```
PLAT199_ALERT_1_G Reported _cell_measurement_temperature ..... (K)        293 Check
PLAT200_ALERT_1_G Reported   _diffrn_ambient_temperature ..... (K)        293 Check
PLAT432_ALERT_2_G Short Inter X...Y Contact  C11      ..C11      .       3.08 Ang.
                                                -x,-y,1-z  =      2_556 Check
PLAT794_ALERT_5_G Tentative Bond Valency for Cu1       (II)      .       2.24 Info
PLAT909_ALERT_3_G Percentage of I>2sig(I) Data at Theta(Max) Still        39% Note
PLAT941_ALERT_3_G Average HKL Measurement Multiplicity ..........        2.5 Low
PLAT961_ALERT_5_G Dataset Contains no Negative Intensities ......     Please Check
PLAT967_ALERT_5_G Note: Two-Theta Cutoff Value in Embedded .res ..       50.1 Degree
PLAT969_ALERT_5_G The 'Henn et al.' R-Factor-gap value ..........      2.007 Note
              Predicted wR2: Based on SigI**2  7.13 or SHELX Weight 14.69
PLAT978_ALERT_2_G Number C-C Bonds with Positive Residual Density.          0 Info
```

---

0 **ALERT level A** = Most likely a serious problem - resolve or explain
0 **ALERT level B** = A potentially serious problem, consider carefully
6 **ALERT level C** = Check. Ensure it is not caused by an omission or oversight
10 **ALERT level G** = General information/check it is not something unexpected

2 ALERT type 1 CIF construction/syntax error, inconsistent or missing data
2 ALERT type 2 Indicator that the structure model may be wrong or deficient
7 ALERT type 3 Indicator that the structure quality may be low
1 ALERT type 4 Improvement, methodology, query or suggestion
4 ALERT type 5 Informative message, check

---

---

It is advisable to attempt to resolve as many as possible of the alerts in all categories. Often the minor alerts point to easily fixed oversights, errors and omissions in your CIF or refinement strategy, so attention to these fine details can be worthwhile. It is up to the individual to critically assess their own results and, if necessary, seek expert advice.

---

**PLATON version of 26/09/2025; check.def file version of 20/09/2025**

---

# duplicate check

**No duplication found**

---

**Datablock cucl2sktl16a** - ellipsoid plot

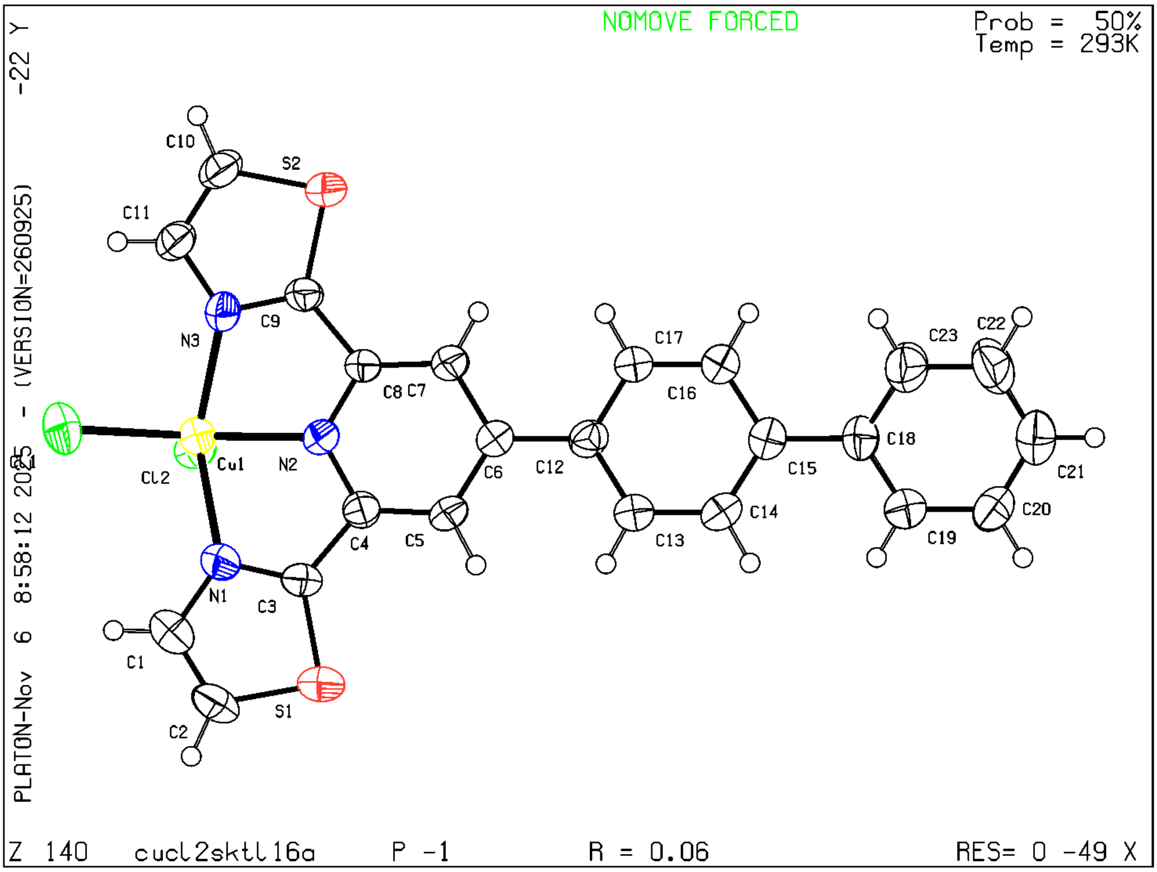

Supplement: Supplementary file 1 [file ijms-26-11868-s001.zip › ESI/checkcif_9.pdf]
