# Supplementary material for: Copper(II) Complexes with 4-Substituted 2,6-Bis(thiazol-2-yl)pyridines—An Overview of Structural–Optical Relationships
Source: Int J Mol Sci. 2025 Dec 9;26(24):11868. doi: 10.3390/ijms262411868 (PMC12733273; doi:10.3390/ijms262411868)
Supplement: Supplementary file 1 [file ijms-26-11868-s001.zip › ESI/Cudtpy_ESI_final.pdf]

Table of Contents

**General Characterization**	
HRMS of complexes **1–13**	Figure S1
IR spectra of the complexes **1–13** in comparison to the free ligands	Figure S2
Comparison of $\nu_{C=N, C=C}$ [cm^{-1}] for Cu(II) complexes with terpy and dtpy	Table S1
**X-ray studies**	
Crystal data and structure refinement	Tables S2-S3
Selected bond lengths and angles	Tables S4-S14
Short intra- and intermolecular hydrogen bonds	Tables S15-S21
Short π•••π interactions	Tables S22-S30
View of the intermolecular interactions and crystal packings	Figures S3-S13
Structural features of Cu(II) compounds based on 2,6-bis(thiazol-2-yl)pyridines (dtpy) derivatives	Table S31
Structural features of Cu(II) compounds based on 2,2′:6′,2″-terpyridine (terpy) derivatives	Table S32
Comparison of Cu-$N_{peripheral}$ bond lengths in groups of copper(II) compounds constructed on terpy (blue diamonds) and dtpy (red squares) derivatives	Figure S14
**Optical properties**	
Absorption band maxima for [Cu(R-dtpy)Cl_2]	Table S33
Absorption band maxima for [Cu(R-terpy)Cl_2]	Table S34
UV-vis spectra of **1**–**6** and **14**–**22** in diluted methanolic solutions, inset: UV-vis spectra of **1**–**6** and **18**–**22** in concentrated methanolic solutions	Figure S15
Wavelengths of the longest absorption bands vs σ_p Hammett’s constants of substituents (R) for [Cu(4-R-dtpy)Cl_2] and [Cu(4′-R-terpy)Cl_2] in diluted methanolic solutions	Figure S16

General Characterization

Figure S1. HRMS of complexes **1–13**.

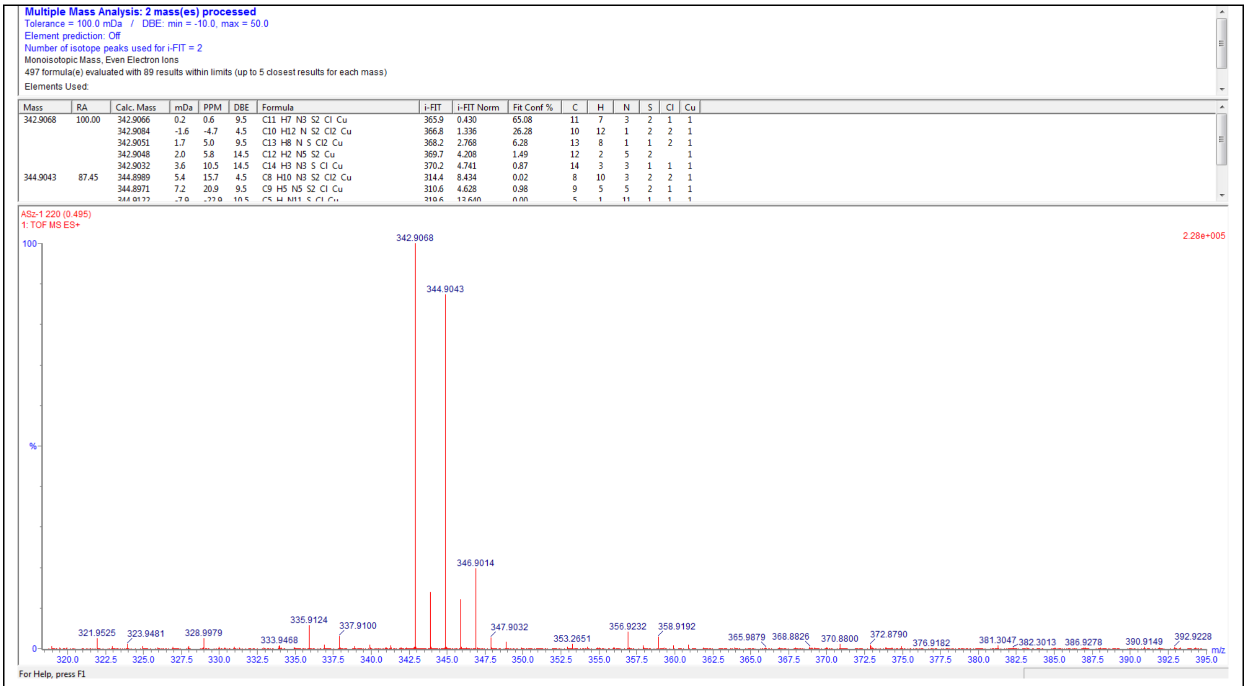

1

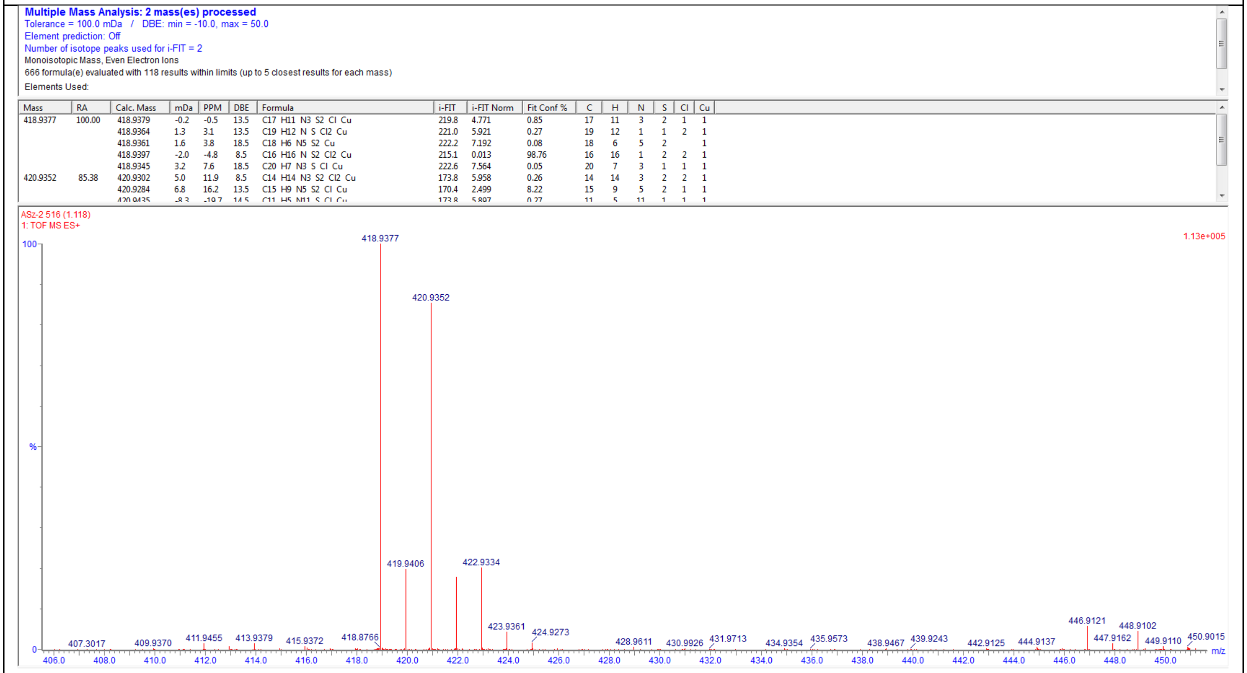

2

Multiple Mass Analysis: 2 mass(es) processed
Tolerance = 100.0 mDa / DBE: min = -10.0, max = 50.0
Element prediction: Off
Number of isotope peaks used for i-FIT = 2
Monoisotopic Mass, Even Electron Ions
672 formula(e) evaluated with 114 results within limits (up to 5 closest results for each mass)
Elements Used:

Mass	RA	Calc. Mass	mDa	PPM	DBE	Formula	i-FIT	i-FIT Norm	Fit Conf %	C	H	N	S	Cl	Cu
419.9334	100.00	419.9331	0.3	0.7	13.5	$C_{16}H_{10}N_4S_2ClCu$	527.2	1.661	19.00	16	10	4	2	1	1
		419.9350	-1.6	-3.8	8.5	$C_{15}H_{15}N_2S_2Cl_2Cu$	532.3	6.794	0.11	15	15	2	2	2	1
		419.9316	1.8	4.3	13.5	$C_{18}H_{11}N_2SCl_2Cu$	525.8	0.224	79.96	18	11	2	1	2	1
		419.9313	2.1	5.0	18.5	$C_{17}H_5N_6S_2Cu$	530.4	4.870	0.77	17	5	6	2		1
		419.9298	3.6	8.6	18.5	$C_{19}H_6N_4SClCu$	532.0	6.411	0.16	19	6	4	1	1	1
421.9310	85.85	421.9255	5.5	13.0	8.5	$C_{13}H_{13}N_4S_2Cl_2Cu$	493.8	7.987	0.03	13	13	4	2	2	1
		421.9236	7.4	17.5	13.5	$C_{14}H_8N_6S_2ClCu$	485.9	0.014	98.58	14	8	6	2	1	1
		[illegible]	[illegible]	[illegible]	[illegible]	[illegible]	[illegible]	[illegible]	[illegible]	[illegible]	[illegible]	[illegible]	[illegible]	[illegible]	[illegible]

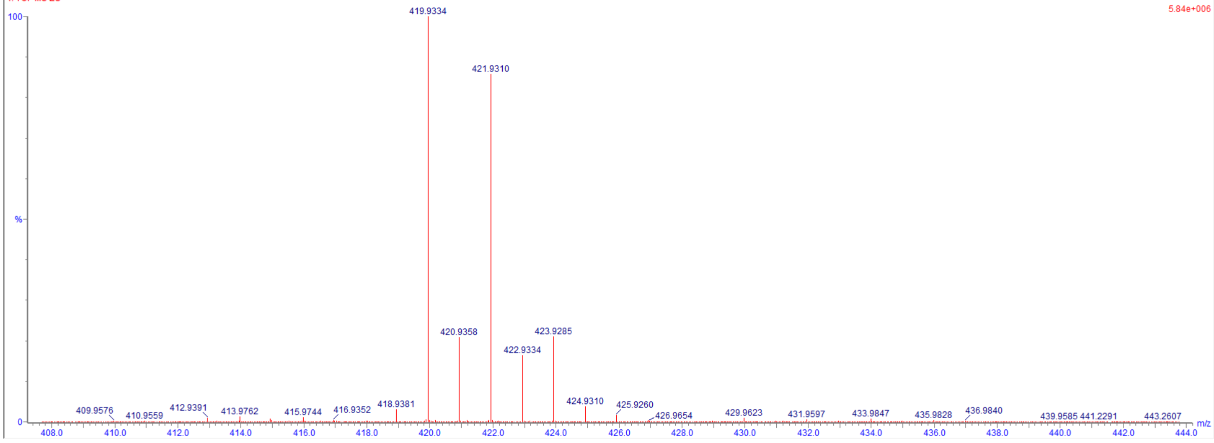

3

Multiple Mass Analysis: 2 mass(es) processed
Tolerance = 100.0 mDa / DBE: min = -10.0, max = 50.0
Element prediction: Off
Number of isotope peaks used for i-FIT = 2
Monoisotopic Mass, Even Electron Ions
7338 formula(e) evaluated with 1380 results within limits (all results (up to 1000) for each mass)
Elements Used:

Mass	RA	Calc. Mass	mDa	PPM	DBE	Formula	i-FIT	i-FIT Norm	Fit Conf %	C	H	N	O	S	Cl	Cu
482.8998	100.00	482.8998	0.0	0.0	13.5	$C_{17}H_{11}N_3O_2S_3ClCu$	393.7	11.669	0.00	17	11	3	2	3	1	1
		482.8996	0.2	0.4	10.5	$C_9H_7N_9O_5S_2ClCu$	397.5	15.437	0.00	9	7	9	5	2	1	1
		482.8994	0.4	0.8	15.5	$C_{12}H_4N_{11}OSCl_2Cu$	393.7	11.694	0.00	12	4	11	1	1	2	1
		482.9003	-0.5	-1.0	19.5	$C_{17}H_3N_7O_3SClCu$	393.9	11.842	0.00	17	3	7	3	1	1	1
		482.9005	-0.7	-1.4	14.5	$C_{14}H_6N_5O_7S_2Cu$	388.8	6.739	0.12	14	6	5	7	2		1
		482.8990	0.8	1.7	1.5	$CH_{11}N_{11}O_7S_3ClCu$	403.1	21.088	0.00	1	11	11	7	3	1	1
		482.8989	0.9	1.9	14.5	$C_{16}H_7N_3O_7SClCu$	385.7	3.688	2.50	16	7	3	7	1	1	1
		[illegible]	[illegible]	[illegible]	[illegible]	[illegible]	[illegible]	[illegible]	[illegible]	[illegible]	[illegible]	[illegible]	[illegible]	[illegible]	[illegible]	[illegible]

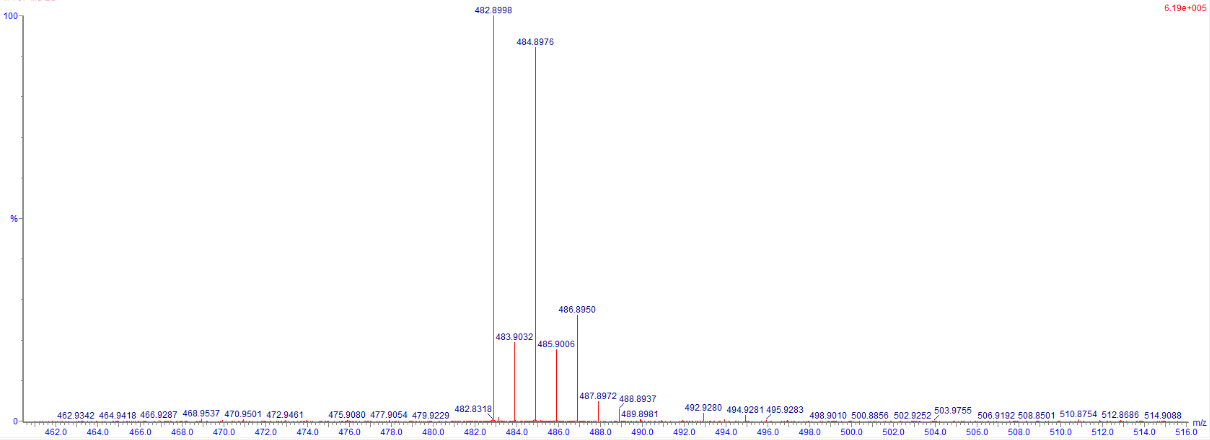

For Help, press F1

4

Multiple Mass Analysis: 5 mass(es) processed
Tolerance = 100.0 mDa / DBE: min = -10.0, max = 50.0
Element prediction: Off
Number of isotope peaks used for i-FIT = 2
Monoisotopic Mass, Even Electron Ions
76 formula(e) evaluated with 14 results within limits (all results (up to 1000) for each mass)
Elements Used:

Mass	RA	Calc. Mass	mDa	PPM	DBE	Formula	i-FIT	i-FIT Norm	Fit Conf %	C	H	N	S	Cl	Cu
506.8821	100.00	506.8820	0.1	0.2	15.5	C19 H11 N3 S4 Cl Cu	560.1	2.872	5.66	19	11	3	4	1	1
		506.8569	25.2	49.7	16.5	C17 H7 N5 S4 Cl Cu	558.7	1.450	23.46	17	7	5	4	1	1
		506.9508	-68.7	-135.5	9.5	C16 H19 N5 S4 Cl Cu	557.6	0.361	69.68	16	19	5	4	1	1
		506.9759	-93.8	-185.1	8.5	C18 H23 N3 S4 Cl Cu	561.7	4.430	1.19	18	23	3	4	1	1
507.8850	21.83	507.8773	7.7	15.2	15.5	C18 H10 N4 S4 Cl Cu	592.7	5.767	0.31	18	10	4	4	1	1
		507.9712	-86.2	-169.7	8.5	C17 H22 N4 S4 Cl Cu	586.9	0.003	99.69	17	22	4	4	1	1
508.8796	95.43	508.8725	7.1	14.0	15.5	C17 H9 N5 S4 Cl Cu	565.2	1.358	25.71	17	9	5	4	1	1
		508.8977	-18.1	-35.6	14.5	C19 H13 N3 S4 Cl Cu	564.2	0.376	68.67	19	13	3	4	1	1

ASz-15 166 (0.372) Cm (152:172)
1: TOF MS ES+

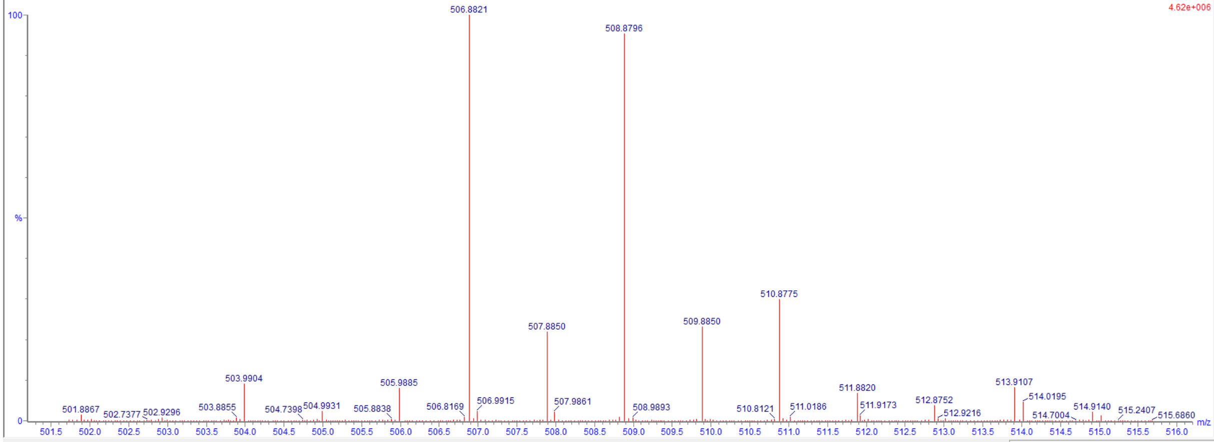

For Help, press F1

5

Multiple Mass Analysis: 5 mass(es) processed
Tolerance = 100.0 mDa / DBE: min = -10.0, max = 50.0
Element prediction: Off
Number of isotope peaks used for i-FIT = 2
Monoisotopic Mass, Even Electron Ions
8980 formula(e) evaluated with 2408 results within limits (up to 5 closest results for each mass)
Elements Used:

Mass	RA	Calc. Mass	mDa	PPM	DBE	Formula	i-FIT	i-FIT Norm	Fit Conf %	C	H	N	O	S	Cl	Cu
434.9330	100.00	434.9328	0.2	0.5	13.5	C17 H11 N3 O S2 Cl Cu	561.1	0.056	94.55	17	11	3	1	2	1	1
		434.9322	0.8	1.8	4.5	C9 H15 N5 O3 S3 Cl Cu	564.8	3.785	2.27	9	15	5	3	3	1	1
		434.9340	-1.0	-2.3	-0.5	C8 H20 N3 O3 S3 Cl2 Cu	565.4	4.362	1.28	8	20	3	3	3	2	1
		434.9326	0.4	0.9	10.5	C9 H7 N9 O4 S Cl Cu	565.5	4.465	1.15	9	7	9	4	1	1	1
		434.9331	-0.1	-0.2	0.5	C7 H16 N3 O8 S Cl2 Cu	565.9	4.887	0.75	7	16	3	8	1	2	1
435.9357	19.15	435.9364	-0.7	-1.6	-4.5	C H19 N8 O4 S3 Cl2 Cu	596.9	0.954	38.53	1	19	8	4	3	2	1
		435.9346	1.1	2.5	0.5	C2 H14 N10 O4 S3 Cl Cu	597.0	1.091	33.59	2	14	10	4	3	1	1
		435.9353	0.4	0.9	9.5	C10 H10 N8 O2 S2 Cl Cu	598.1	2.132	11.86	10	10	8	2	2	1	1

ASz-6 383 (0.845) Cm (376:394)
1: TOF MS ES+

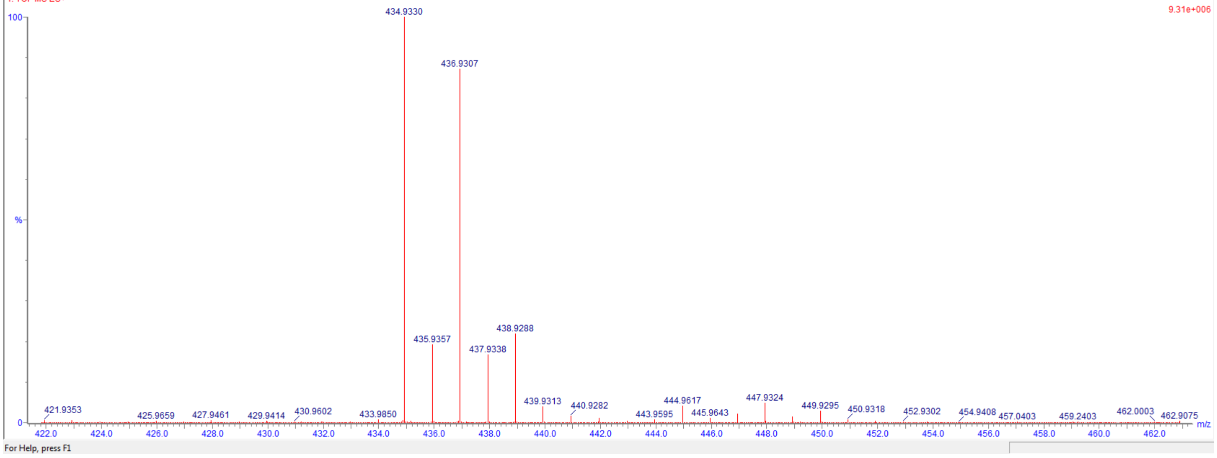

For Help, press F1

6

Multiple Mass Analysis: 5 mass(es) processed

Tolerance = 100.0 mDa / DBE: min = -10.0, max = 50.0

Element prediction: Off

Number of isotope peaks used for i-FIT = 2

Monoisotopic Mass, Odd and Even Electron Ions

25 formula(e) evaluated with 9 results within limits (all results (up to 1000) for each mass)

Elements Used:

Mass	RA	Calc. Mass	mDa	PPM	DBE	Formula	i-FIT	i-FIT Norm	Fit Conf %	C	H	N	S	Cl	Cu
443.9331	100.00	443.9331	0.0	0.0	15.5	C18 H10 N4 S2 Cl Cu	577.9	5.847	0.29	18	10	4	2	1	1
		444.0270	-93.9	-211.5	8.5	C17 H22 N4 S2 Cl Cu	572.0	0.003	99.71	17	22	4	2	1	1
444.9368	21.75	444.9410	-4.2	-9.4	15.0	C18 H11 N4 S2 Cl Cu	607.5	0.001	99.95	18	11	4	2	1	1
		445.0349	-98.1	-220.5	8.0	C17 H23 N4 S2 Cl Cu	615.1	7.526	0.05	17	23	4	2	1	1
445.9308	86.57	445.9488	-18.0	-40.4	14.5	C18 H12 N4 S2 Cl Cu	573.4	n/a	n/a	18	12	4	2	1	1
446.9380	21.05	446.9566	-18.6	-41.6	14.0	C18 H13 N4 S2 Cl Cu	556.4	0.680	50.67	18	13	4	2	1	1
		446.8627	75.3	168.5	21.0	C19 H N4 S2 Cl Cu	556.5	0.707	49.33	19	1	4	2	1	1
447.9294	24.51	447.9644	-35.0	-78.1	13.5	C18 H14 N4 S2 Cl Cu	584.0	0.136	87.32	18	14	4	2	1	1

ASz-7 925 (1.995) Cm (919:946)
1: TOF MS ES+

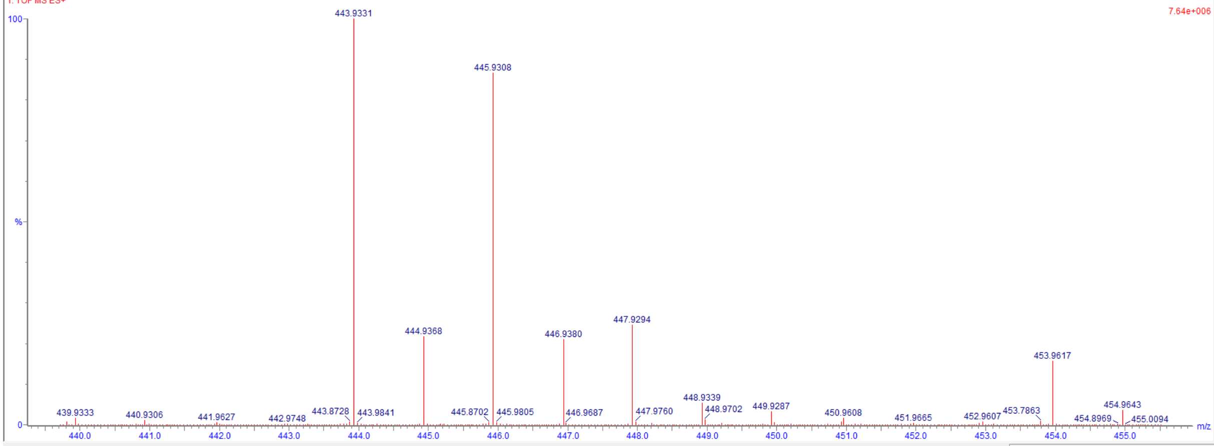

For Help, press F1

7

Multiple Mass Analysis: 3 mass(es) processed

Tolerance = 100.0 mDa / DBE: min = -10.0, max = 50.0

Element prediction: Off

Number of isotope peaks used for i-FIT = 2

Monoisotopic Mass, Even Electron Ions

13 formula(e) evaluated with 5 results within limits (all results (up to 1000) for each mass)

Elements Used:

Mass	RA	Calc. Mass	mDa	PPM	DBE	Formula	i-FIT	i-FIT Norm	Fit Conf %	C	H	N	S	Cl	Cu	Br
496.8490	53.78	496.8484	0.6	1.2	13.5	C17 H10 N3 S2 Cl Cu Br	532.9	0.213	80.80	17	10	3	2	1	1	1
		496.9423	-93.3	-187.8	6.5	C16 H22 N3 S2 Cl Cu Br	534.4	1.650	19.20	16	22	3	2	1	1	1
498.8472	100.00	498.8641	-16.9	-33.9	12.5	C17 H12 N3 S2 Cl Cu Br	538.8	n/a	n/a	17	12	3	2	1	1	1
500.8448	57.68	500.8797	-34.9	-69.7	11.5	C17 H14 N3 S2 Cl Cu Br	506.3	0.199	82.00	17	14	3	2	1	1	1
		500.7858	59.0	117.8	18.5	C18 H2 N3 S2 Cl Cu Br	507.8	1.715	18.00	18	2	3	2	1	1	1

ASz-8 105 (0.256) Cm (104:114)
1: TOF MS ES+

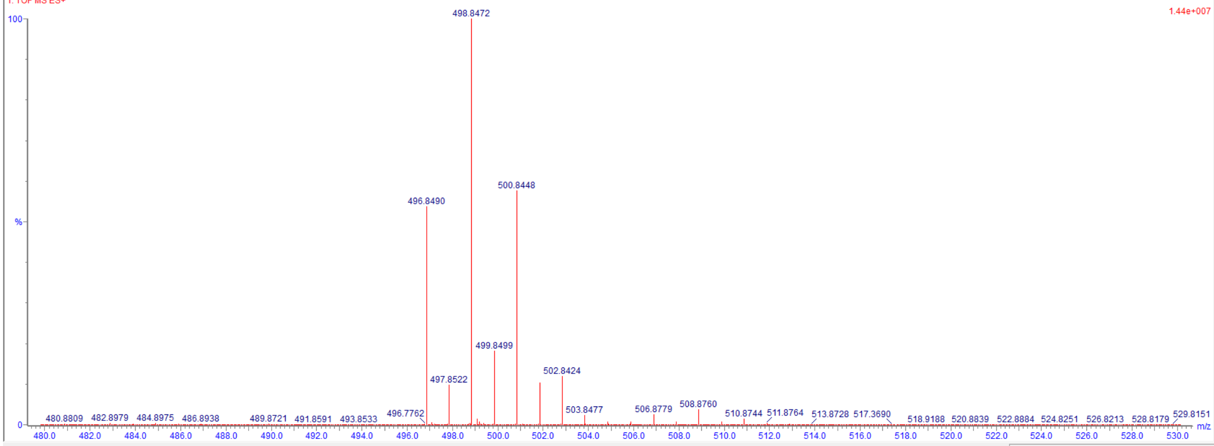

For Help, press F1

8

Multiple Mass Analysis: 5 mass(es) processed
Tolerance = 100.0 mDa / DBE: min = -10.0, max = 50.0
Element prediction: Off
Number of isotope peaks used for i-FIT = 2
Monoisotopic Mass, Even Electron Ions
30 formula(e) evaluated with 7 results within limits (all results (up to 1000) for each mass)
Elements Used:

Mass	RA	Calc. Mass	mDa	PPM	DBE	Formula	i-FIT	i-FIT Norm	Fit Conf %	C	H	N	S	Cl	Cu
494.9697	100.00	494.9692	0.5	1.0	17.5	C23 H15 N3 S2 Cl Cu	638.1	0.427	65.25	23	15	3	2	1	1
		495.0631	-93.4	-188.7	10.5	C22 H27 N3 S2 Cl Cu	638.8	1.099	33.32	22	27	3	2	1	1
		494.8753	94.4	190.7	24.5	C24 H3 N3 S2 Cl Cu	641.9	4.248	1.43	24	3	3	2	1	1
495.9724	25.74	---													
496.9675	89.84	496.9848	-17.3	-34.8	16.5	C23 H17 N3 S2 Cl Cu	627.9	0.058	94.34	23	17	3	2	1	1
		496.8909	76.6	154.1	23.5	C24 H5 N3 S2 Cl Cu	630.7	2.871	5.66	24	5	3	2	1	1
497.9701	22.46	---													
498.9652	23.34	499.0005	-35.3	-70.7	15.5	C23 H19 N3 S2 Cl Cu	557.4	0.223	80.03	23	19	3	2	1	1

ASz-9 320 (0.705) Cm (316:336)
1: TOF MS ES+

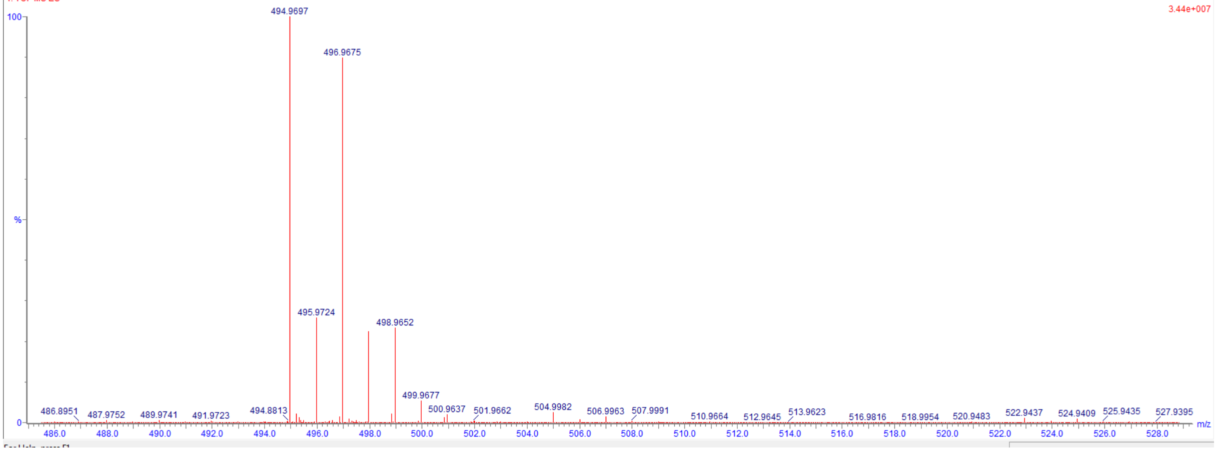

9

Multiple Mass Analysis: 5 mass(es) processed
Tolerance = 100.0 mDa / DBE: min = -10.0, max = 50.0
Element prediction: Off
Number of isotope peaks used for i-FIT = 2
Monoisotopic Mass, Even Electron Ions
82 formula(e) evaluated with 17 results within limits (all results (up to 1000) for each mass)
Elements Used:

Mass	RA	Calc. Mass	mDa	PPM	DBE	Formula	i-FIT	i-FIT Norm	Fit Conf %	C	H	N	S	Cl	Cu
500.9913	100.00	500.9910	0.3	0.6	15.5	C21 H17 N5 S2 Cl Cu	595.5	0.447	63.95	21	17	5	2	1	1
		501.0161	-24.8	-49.5	14.5	C23 H21 N3 S2 Cl Cu	596.7	1.660	19.01	23	21	3	2	1	1
		500.9222	69.1	137.9	21.5	C24 H9 N3 S2 Cl Cu	598.7	3.629	2.65	24	9	3	2	1	1
		501.0849	-93.6	-186.8	8.5	C20 H29 N5 S2 Cl Cu	597.2	2.132	11.87	20	29	5	2	1	1
		500.8971	94.2	188.0	22.5	C22 H5 N5 S2 Cl Cu	598.7	3.681	2.52	22	5	5	2	1	1
501.9943	22.86	502.0114	-17.1	-34.1	14.5	C22 H20 N4 S2 Cl Cu	647.9	0.649	52.24	22	20	4	2	1	1
		501.9175	76.8	153.0	21.5	C23 H8 N4 S2 Cl Cu	648.0	0.739	47.76	23	8	4	2	1	1
502.9891	86.69	503.0066	-17.5	-34.8	14.5	C21 H19 N5 S2 Cl Cu	586.4	0.329	71.99	21	19	5	2	1	1

ASz-10 364 (0.809) Cm (359:375)
1: TOF MS ES+

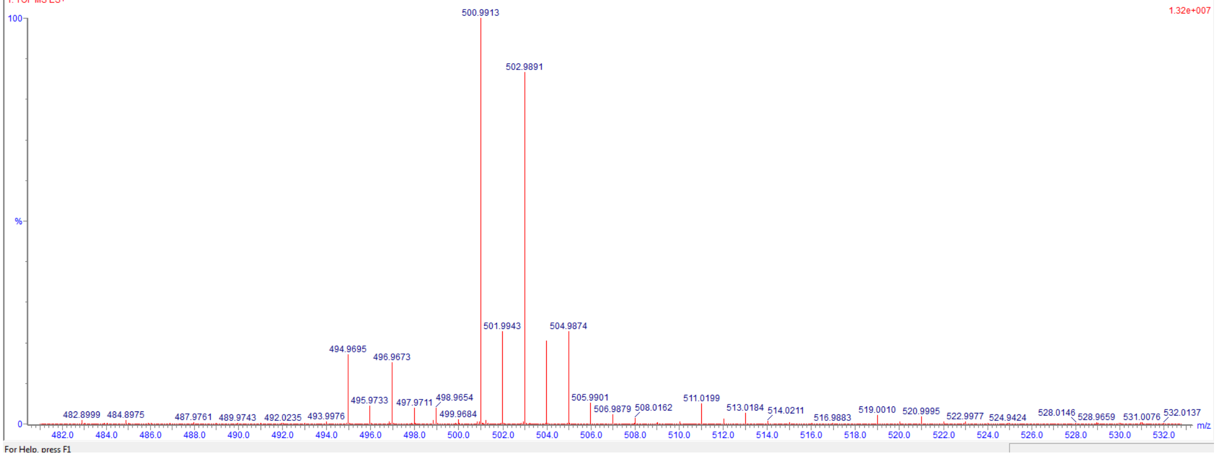

For Help, press F1

10

Multiple Mass Analysis: 5 mass(es) processed
Tolerance = 100.0 mDa / DBE: min = -10.0, max = 50.0
Element prediction: Off
Number of isotope peaks used for i-FIT = 2
Monoisotopic Mass, Even Electron Ions
77 formula(e) evaluated with 13 results within limits (all results (up to 1000) for each mass)
Elements Used:

Mass	RA	Calc. Mass	mDa	PPM	DBE	Formula	i-FIT	i-FIT Norm	Fit Conf %	C	H	N	S	Cl	Cu
461.9803	100.00	461.9801	0.2	0.4	13.5	C19 H16 N4 S2 Cl Cu	436.1	0.303	73.89	19	16	4	2	1	1
		462.0740	-93.7	-202.8	6.5	C18 H28 N4 S2 Cl Cu	437.3	1.491	22.52	18	28	4	2	1	1
		461.8862	94.1	203.7	20.5	C20 H4 N4 S2 Cl Cu	439.1	3.326	3.59	20	4	4	2	1	1
462.9836	20.98	462.9753	8.3	17.9	13.5	C18 H15 N5 S2 Cl Cu	442.2	1.345	26.05	18	15	5	2	1	1
		463.0005	-16.9	-36.5	12.5	C20 H19 N3 S2 Cl Cu	442.3	1.391	24.88	20	19	3	2	1	1
		462.9066	77.0	166.3	19.5	C21 H7 N3 S2 Cl Cu	442.3	1.475	22.88	21	7	3	2	1	1
		463.0692	-85.6	-184.9	6.5	C17 H27 N5 S2 Cl Cu	442.2	1.340	26.19	17	27	5	2	1	1
463.9780	86.29	463.9957	-17.7	-38.1	12.5	C19 H18 N4 S2 Cl Cu	358.1	0.089	91.47	19	18	4	2	1	1

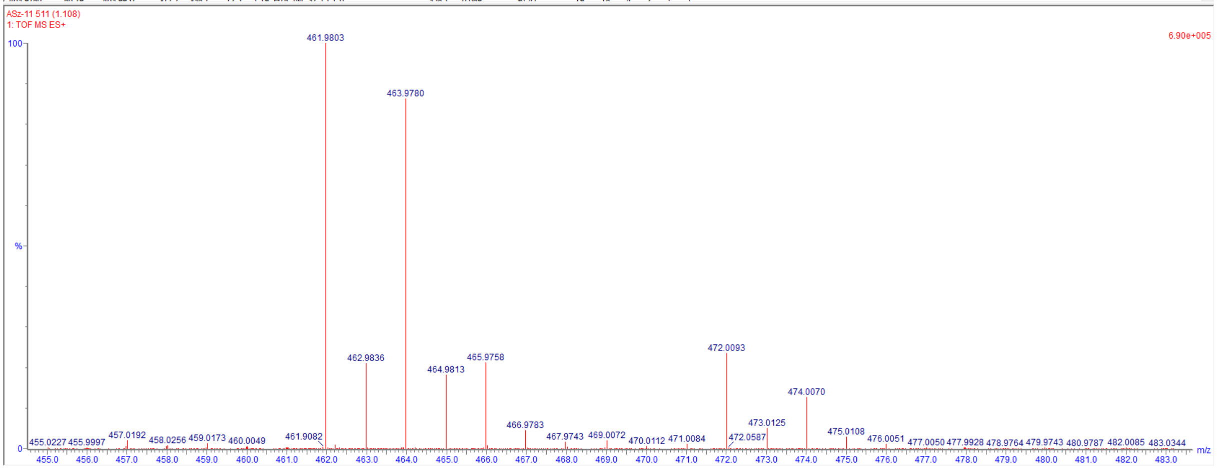

11

Multiple Mass Analysis: 5 mass(es) processed
Tolerance = 100.0 mDa / DBE: min = -10.0, max = 50.0
Element prediction: Off
Number of isotope peaks used for i-FIT = 2
Monoisotopic Mass, Even Electron Ions
78 formula(e) evaluated with 15 results within limits (all results (up to 1000) for each mass)
Elements Used:

Mass	RA	Calc. Mass	mDa	PPM	DBE	Formula	i-FIT	i-FIT Norm	Fit Conf %	C	H	N	S	Cl	Cu
487.9958	100.00	487.9957	0.1	0.2	14.5	C21 H18 N4 S2 Cl Cu	441.5	0.230	79.48	21	18	4	2	1	1
		488.0896	-93.8	-192.2	7.5	C20 H30 N4 S2 Cl Cu	443.1	1.786	16.77	20	30	4	2	1	1
		487.9018	94.0	192.6	21.5	C22 H6 N4 S2 Cl Cu	444.6	3.282	3.76	22	6	4	2	1	1
488.9991	22.59	488.9910	8.1	16.6	14.5	C20 H17 N5 S2 Cl Cu	440.0	1.339	26.20	20	17	5	2	1	1
		489.0161	-17.0	-34.8	13.5	C22 H21 N3 S2 Cl Cu	440.0	1.389	24.94	22	21	3	2	1	1
		488.9222	76.9	157.3	20.5	C23 H9 N3 S2 Cl Cu	440.1	1.476	22.85	23	9	3	2	1	1
		489.0849	-85.8	-175.5	7.5	C19 H29 N5 S2 Cl Cu	440.0	1.347	26.01	19	29	5	2	1	1
489.9939	86.22	490.0114	-17.5	-35.7	13.5	C21 H20 N4 S2 Cl Cu	394.0	0.116	89.08	21	20	4	2	1	1

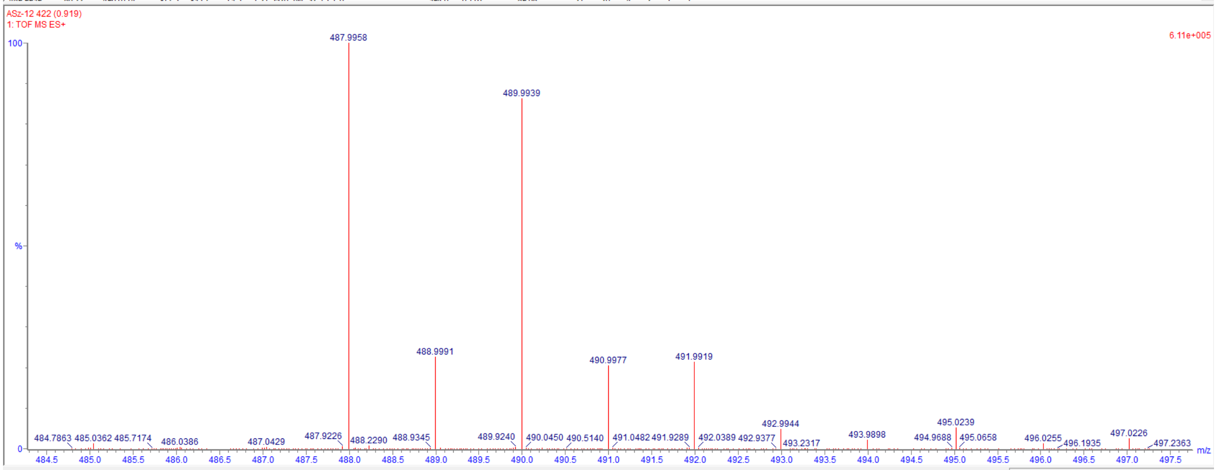

12

Multiple Mass Analysis: 5 mass(es) processed
Tolerance = 100.0 mDa / DBE: min = -10.0, max = 50.0
Element prediction: Off
Number of isotope peaks used for i-FIT = 2
Monoisotopic Mass, Even Electron Ions
78 formula(e) evaluated with 15 results within limits (all results (up to 1000) for each mass)
Elements Used:

Mass	RA	Calc. Mass	mDa	PPM	DBE	Formula	i-FIT	i-FIT Norm	Fit Conf %	C	H	N	O	S	Cl	Cu
503.9910	100.00	503.9907	0.3	0.6	14.5	C21 H18 N4 O S2 Cl Cu	442.3	0.176	83.83	21	18	4	1	2	1	1
		504.0846	-93.6	-185.7	7.5	C20 H30 N4 O S2 Cl Cu	444.2	2.038	13.03	20	30	4	1	2	1	1
		503.8968	94.2	186.9	21.5	C22 H6 N4 O S2 Cl Cu	445.6	3.462	3.14	22	6	4	1	2	1	1
504.9938	22.05	504.9859	7.9	15.6	14.5	C20 H17 N5 O S2 Cl Cu	489.3	1.340	26.19	20	17	5	1	2	1	1
		505.0111	-17.3	-34.3	13.5	C22 H21 N3 O S2 Cl Cu	489.4	1.384	25.06	22	21	3	1	2	1	1
		504.9172	76.6	151.7	20.5	C23 H9 N3 O S2 Cl Cu	489.5	1.482	22.72	23	9	3	1	2	1	1
		505.0798	-86.0	-170.3	7.5	C19 H29 N5 O S2 Cl Cu	489.3	1.346	26.03	19	29	5	1	2	1	1
505.9891	88.40	506.0063	-17.2	-34.0	13.5	C21 H20 N4 O S2 Cl Cu	436.3	0.106	89.94	21	20	4	1	2	1	1

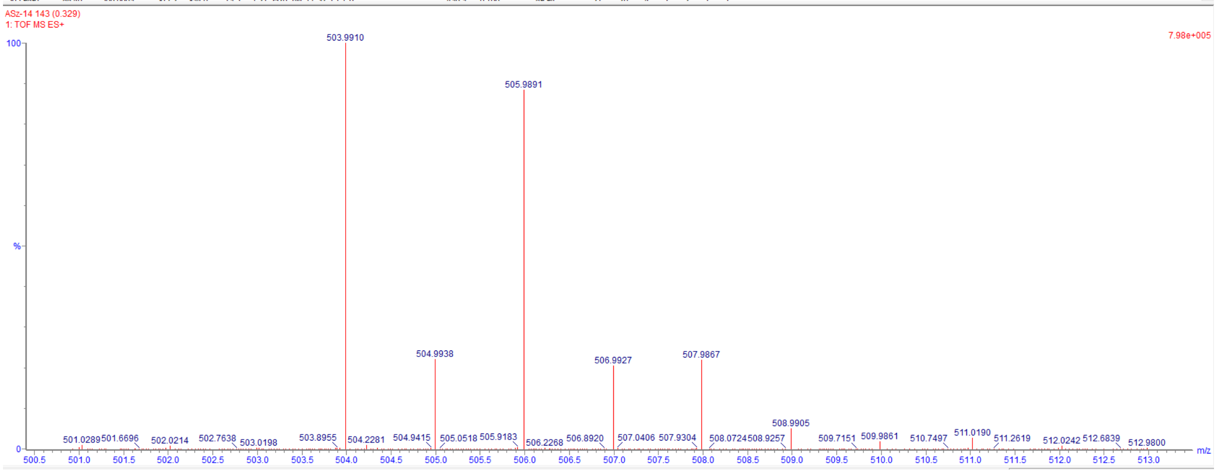

Figure S2. IR spectra of the complexes **1–13** in comparison to the free ligands.

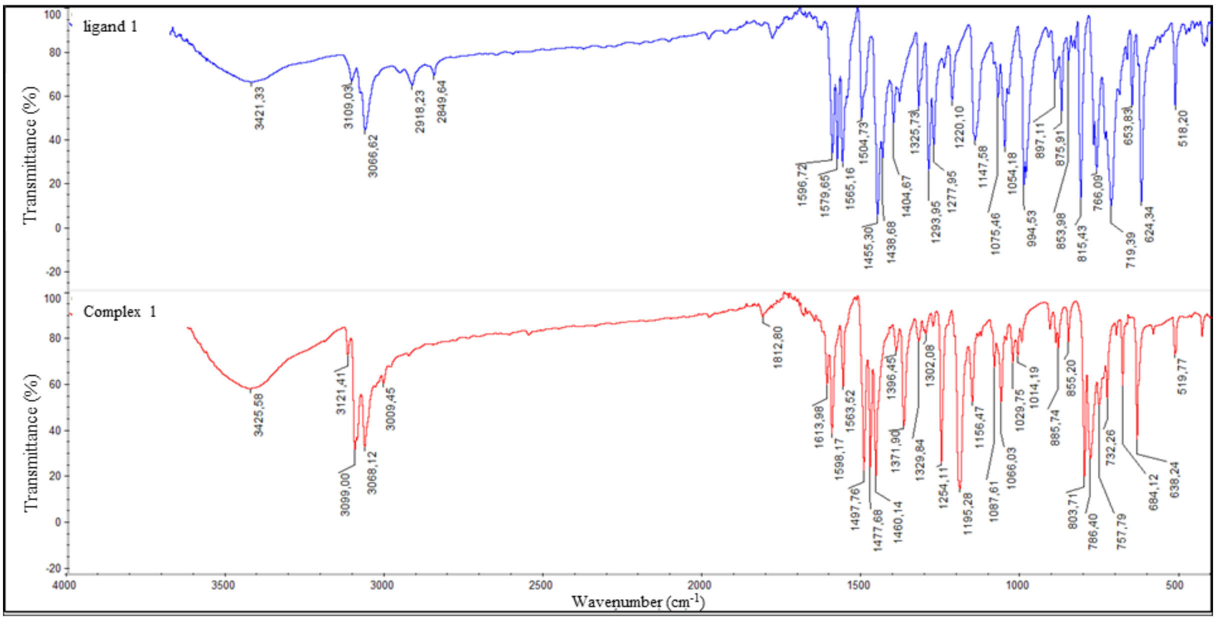

1

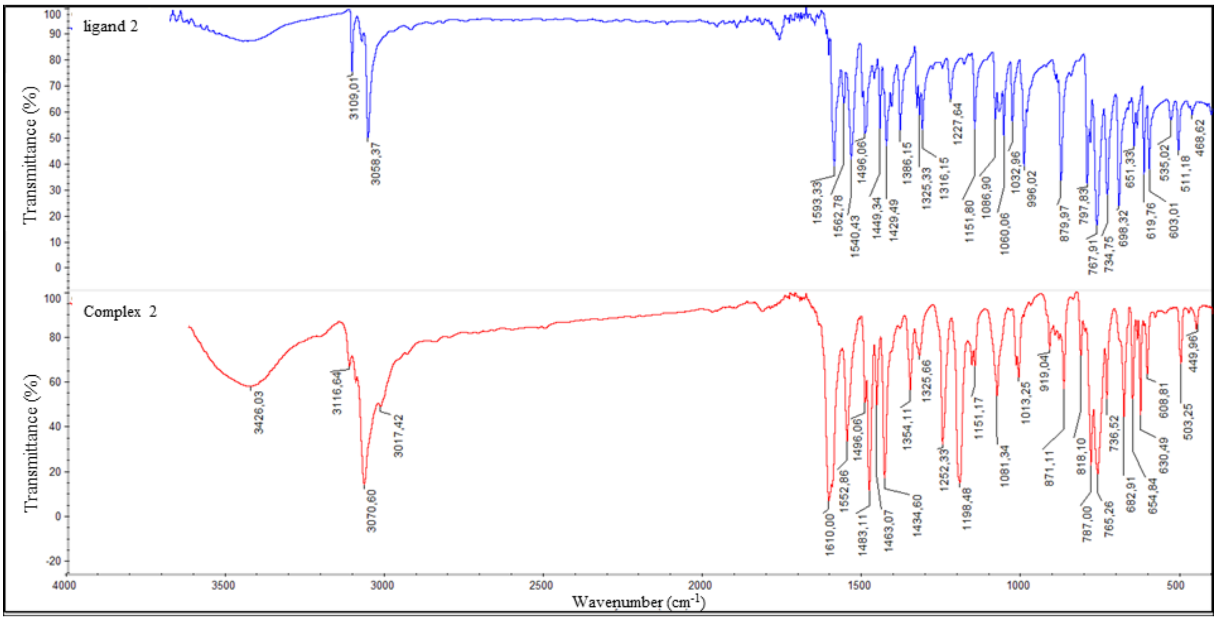

2

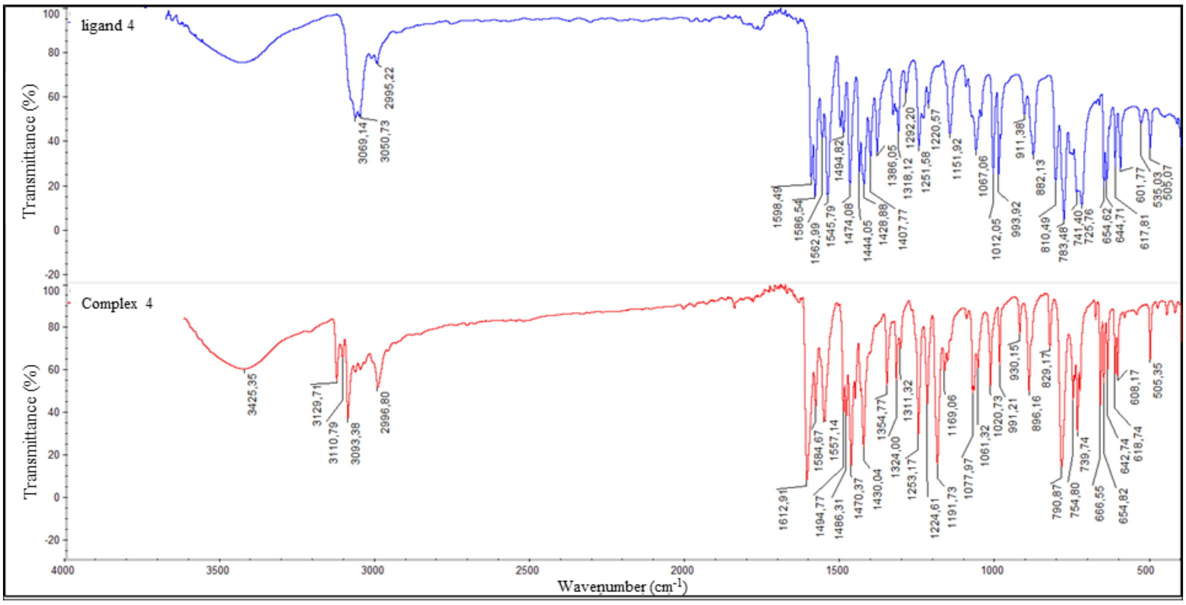
ligand 4
Complex 4
Transmittance (%)
Wavenumber (cm-1)
3069,14
3050,73
2995,22
1598,49
1586,54
1562,99
1545,79
1494,82
1474,08
1444,05
1428,88
1407,77
1386,05
1318,12
1292,20
1251,58
1220,57
1151,92
1067,06
1012,05
993,92
911,38
882,13
810,49
783,48
741,40
725,76
654,62
644,71
617,81
601,77
535,03
505,07
3425,35
3129,71
3110,79
3093,38
2996,80
1612,91
1584,67
1557,14
1494,77
1486,31
1470,37
1430,04
1354,77
1324,00
1311,32
1253,17
1224,61
1191,73
1169,06
1077,97
1061,32
1020,73
991,21
930,15
896,16
829,17
790,87
754,80
739,74
666,55
654,82
642,74
618,74
608,17
505,35

3

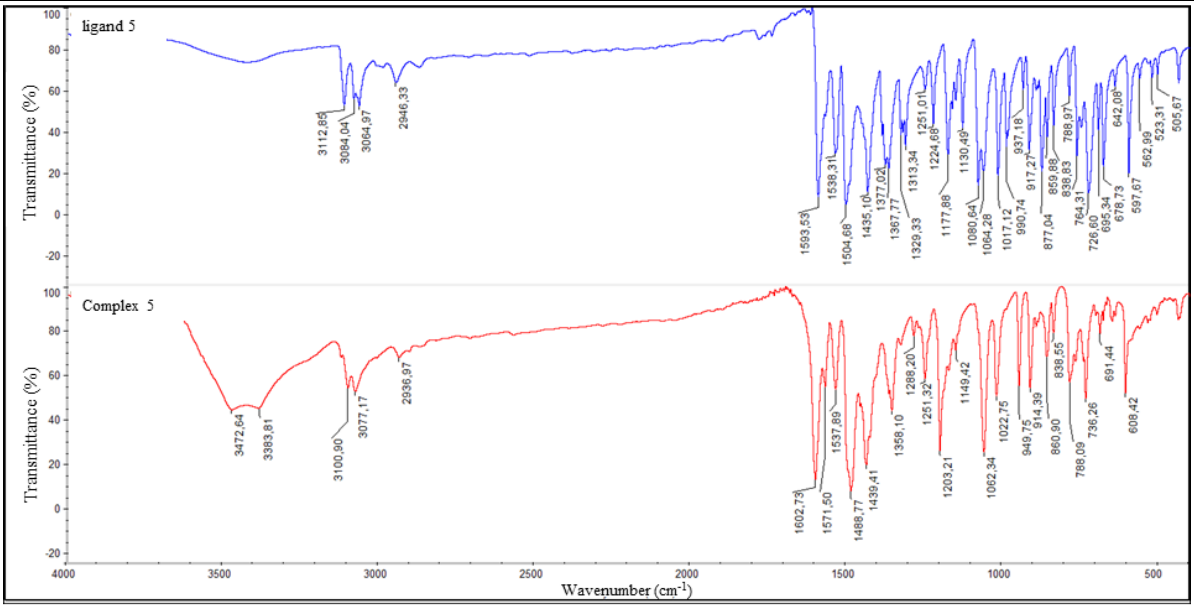
ligand 5
Complex 5
Transmittance (%)
Wavenumber (cm-1)
3112,85
3084,04
3064,97
2946,33
1593,53
1538,31
1504,68
1435,10
1377,02
1367,77
1329,33
1313,34
1251,01
1224,68
1177,88
1130,49
1080,64
1064,28
1017,12
990,74
937,18
917,27
877,04
859,88
838,83
788,97
764,31
726,60
695,34
678,73
642,08
597,67
562,99
523,31
505,67
3472,64
3383,81
3100,90
3077,17
2936,97
1602,73
1571,50
1537,89
1488,77
1439,41
1358,10
1288,20
1251,32
1203,21
1149,42
1062,34
1022,75
949,75
914,39
860,90
838,55
788,09
736,26
691,44
608,42

4

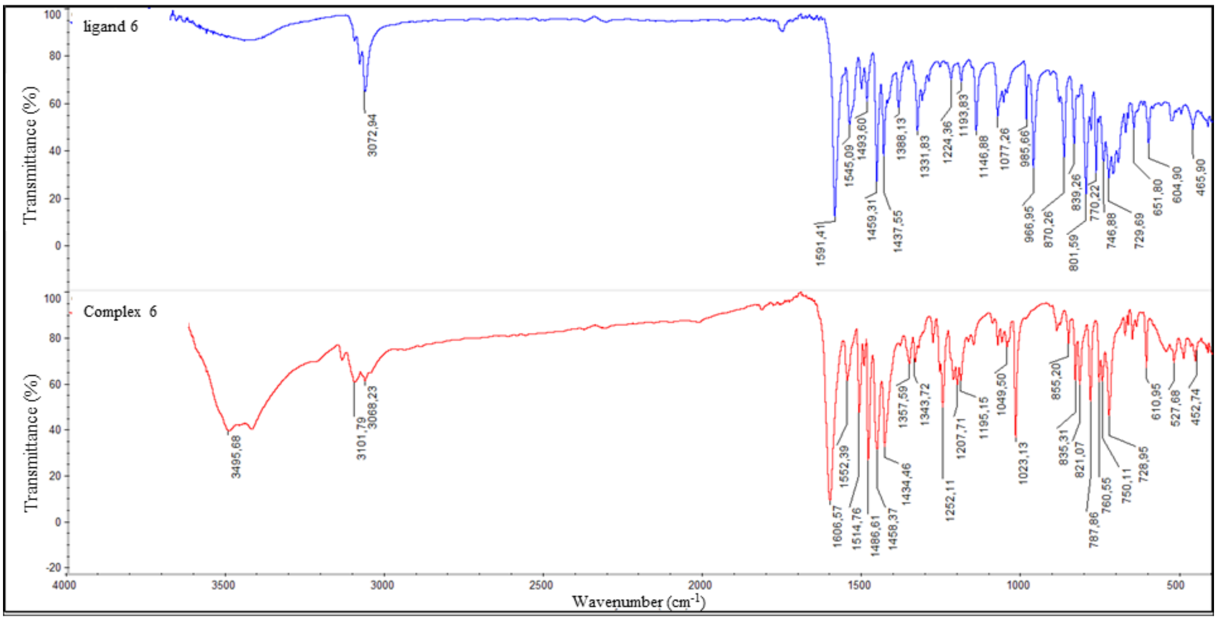

ligand 6
Complex 6
Transmittance (%)
Wavenumber (cm-1)
3072,94
1591,41
1545,09
1493,60
1459,31
1437,55
1388,13
1331,83
1224,36
1193,83
1146,88
1077,26
985,66
966,95
870,26
839,26
801,59
770,22
746,88
729,69
651,80
604,90
465,90
3495,68
3101,79
3068,23
1606,57
1552,39
1514,76
1486,61
1458,37
1434,46
1357,59
1343,72
1252,11
1207,71
1195,15
1049,50
1023,13
855,20
835,31
821,07
787,86
760,55
750,11
728,95
610,95
527,68
452,74

5

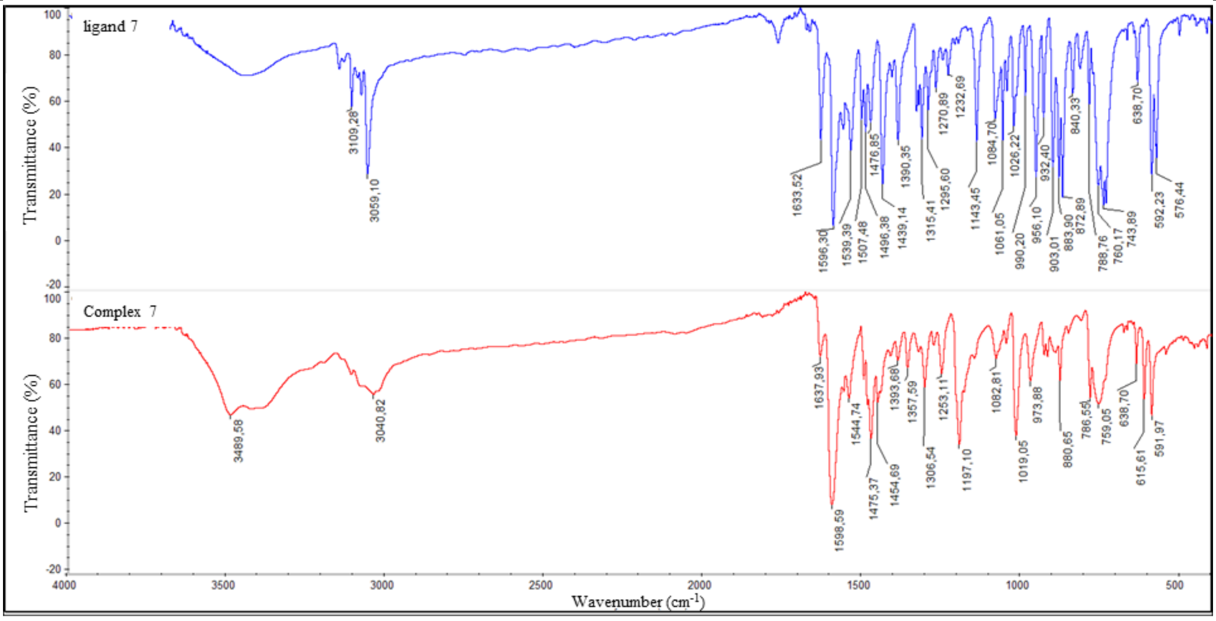

ligand 7
Complex 7
Transmittance (%)
Wavenumber (cm-1)
3109,28
3059,10
1633,52
1596,30
1539,39
1507,48
1496,38
1476,85
1439,14
1390,35
1315,41
1295,60
1270,89
1232,69
1143,45
1084,70
1061,05
1026,22
990,20
956,10
932,40
903,01
883,90
872,89
840,33
788,76
760,17
743,89
638,70
592,23
576,44
3489,58
3040,82
1637,93
1598,59
1544,74
1475,37
1454,69
1393,68
1357,59
1306,54
1253,11
1197,10
1082,81
1019,05
973,88
880,65
786,55
759,05
638,70
615,61
591,97

6

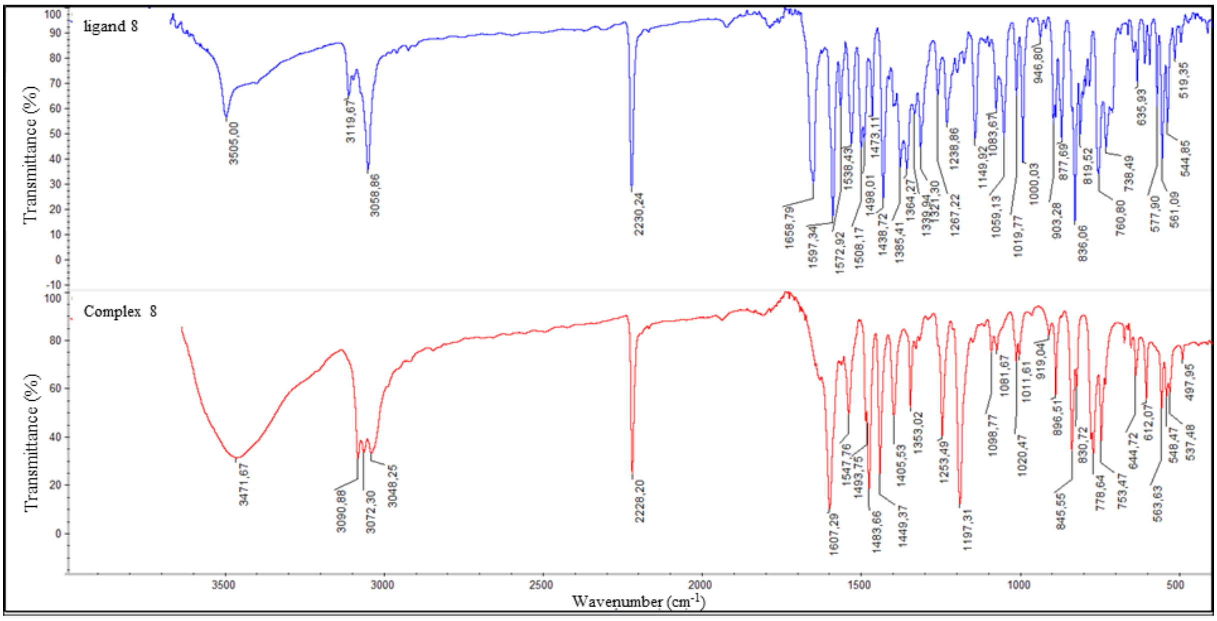

ligand 8
Complex 8
Transmittance (%)
Transmittance (%)
Wavenumber (cm-1)
3505,00
3119,67
3058,86
2230,24
1658,79
1597,34
1572,92
1538,43
1508,17
1498,01
1473,11
1438,72
1385,41
1364,27
1321,30
1267,22
1238,86
1149,92
1083,67
1059,13
1019,77
1000,03
946,80
903,28
877,69
836,06
819,52
760,80
738,49
635,93
577,90
561,09
544,85
519,35
3471,67
3090,88
3072,30
3048,25
2228,20
1607,29
1547,76
1493,75
1483,66
1449,37
1405,53
1353,02
1253,49
1197,31
1098,77
1081,67
1020,47
1011,61
919,04
896,51
845,55
830,72
778,64
753,47
644,72
612,07
563,63
548,47
537,48
497,95

7

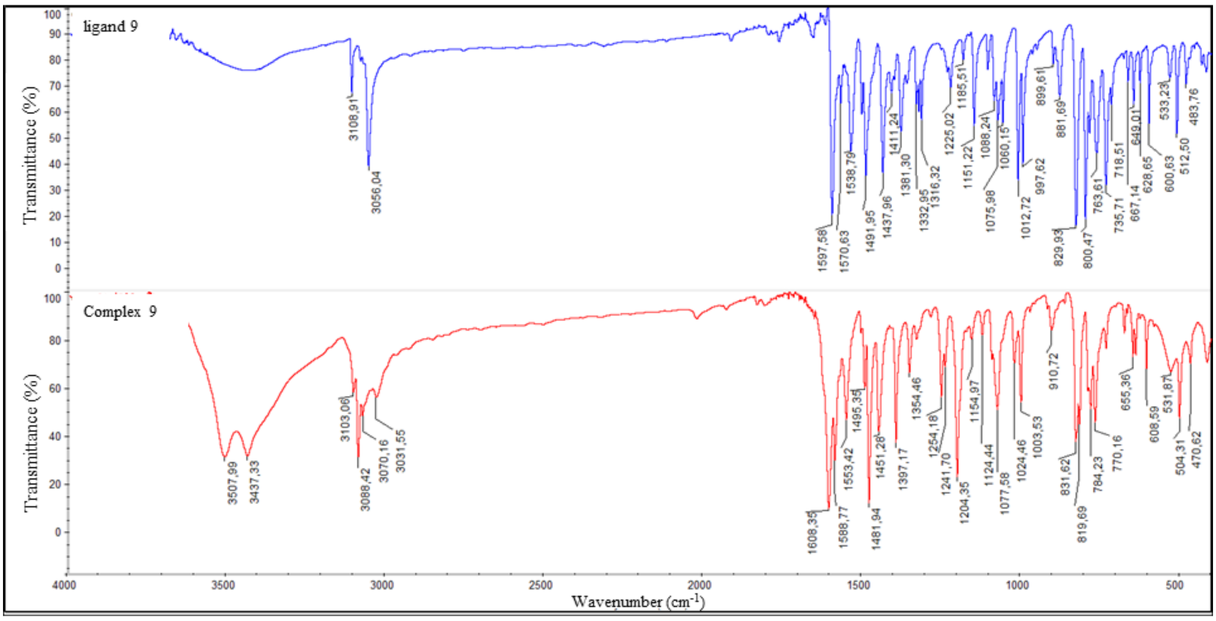

ligand 9
Complex 9
Transmittance (%)
Transmittance (%)
Wavenumber (cm-1)
3108,91
3056,04
1597,58
1570,63
1538,79
1491,95
1437,96
1411,24
1381,30
1332,95
1316,32
1225,02
1185,51
1151,22
1088,24
1075,98
1060,15
1012,72
997,62
899,61
881,69
829,93
800,47
763,61
735,71
718,51
667,14
649,01
628,65
600,63
533,23
512,50
483,76
3507,99
3437,33
3103,06
3088,42
3070,16
3031,55
1608,35
1588,77
1553,42
1495,35
1481,94
1451,28
1397,17
1354,46
1254,18
1241,70
1204,35
1154,97
1124,44
1077,58
1024,46
1003,53
910,72
831,62
819,69
784,23
770,16
655,36
608,59
531,87
504,31
470,62

8

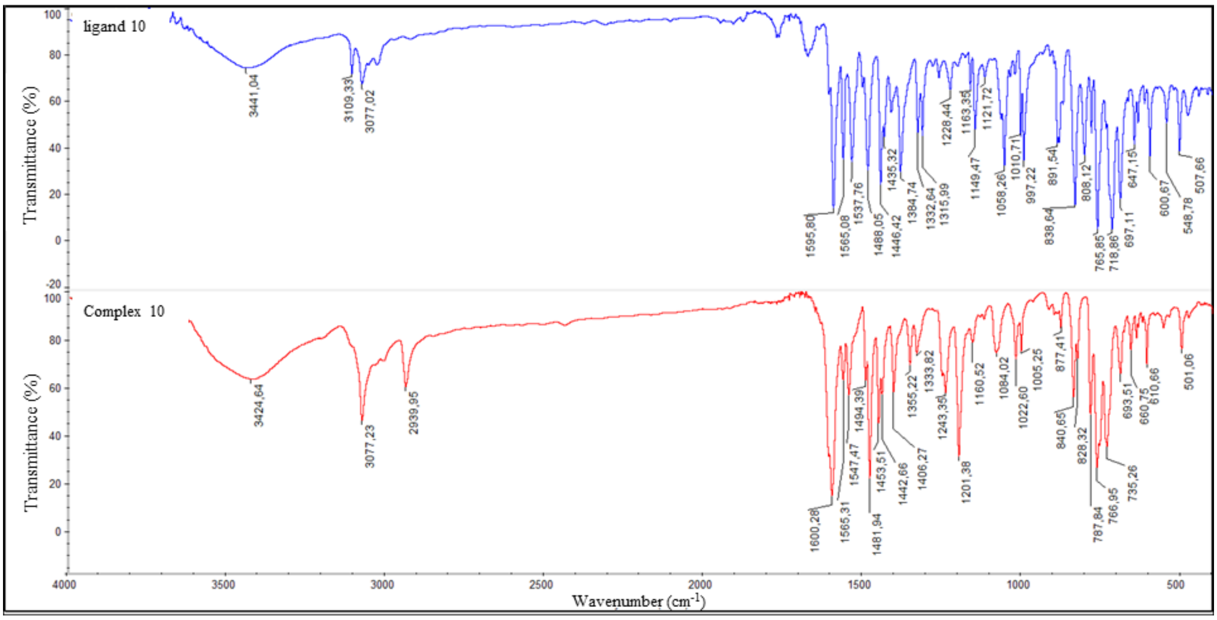
ligand 10
Complex 10
Transmittance (%)
Wavenumber (cm-1)
3441,04
3109,33
3077,02
1595,80
1565,08
1537,76
1488,05
1446,42
1435,32
1384,74
1332,64
1315,99
1228,44
1163,35
1149,47
1121,72
1058,26
1010,71
997,22
891,54
838,64
808,12
765,85
718,86
697,11
647,15
600,67
548,78
507,66
3424,64
3077,23
2939,95
1600,28
1565,31
1547,47
1494,39
1481,94
1453,51
1442,66
1406,27
1355,22
1333,82
1243,35
1201,38
1160,52
1084,02
1022,60
1005,25
877,41
840,65
828,32
787,84
766,95
735,26
693,51
660,75
610,66
501,06

9

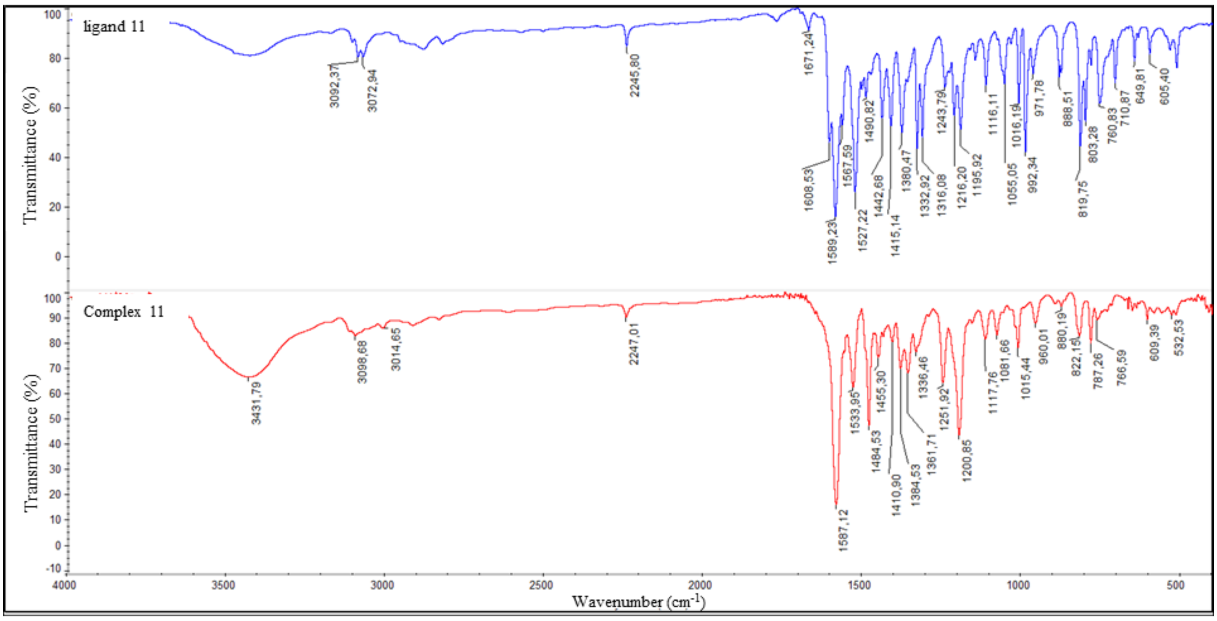
ligand 11
Complex 11
Transmittance (%)
Wavenumber (cm-1)
3092,37
3072,94
2245,80
1671,24
1608,53
1589,23
1567,59
1527,22
1490,82
1442,68
1415,14
1380,47
1332,92
1316,08
1243,79
1216,20
1195,92
1116,11
1055,05
1016,19
992,34
971,78
888,51
819,75
803,28
760,83
710,87
649,81
605,40
3431,79
3098,68
3014,65
2247,01
1587,12
1533,95
1484,53
1455,30
1410,90
1384,53
1361,71
1336,46
1251,92
1200,85
1117,76
1081,66
1015,44
960,01
880,19
822,15
787,26
766,59
609,39
532,53

10

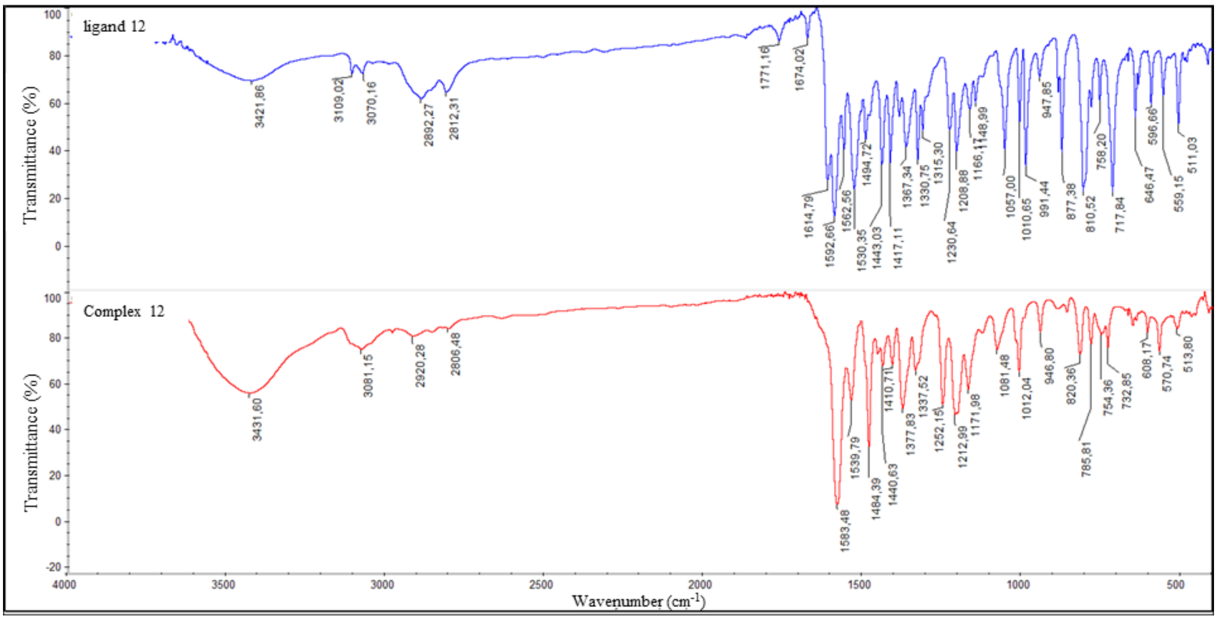
ligand 12
Complex 12
Transmittance (%)
Wavenumber (cm-1)
3421,86
3109,02
3070,16
2892,27
2812,31
1771,16
1674,02
1614,79
1592,66
1562,56
1530,35
1494,72
1443,03
1417,11
1367,34
1330,75
1315,30
1230,64
1208,88
1166,17
1148,99
1057,00
1010,65
991,44
947,85
877,38
810,52
758,20
717,84
646,47
596,66
559,15
511,03
3431,60
3081,15
2920,28
2806,48
1583,48
1539,79
1484,39
1440,63
1410,71
1377,83
1337,52
1252,15
1212,99
1171,98
1081,48
1012,04
946,80
820,36
785,81
754,36
732,85
608,17
570,74
513,80

11

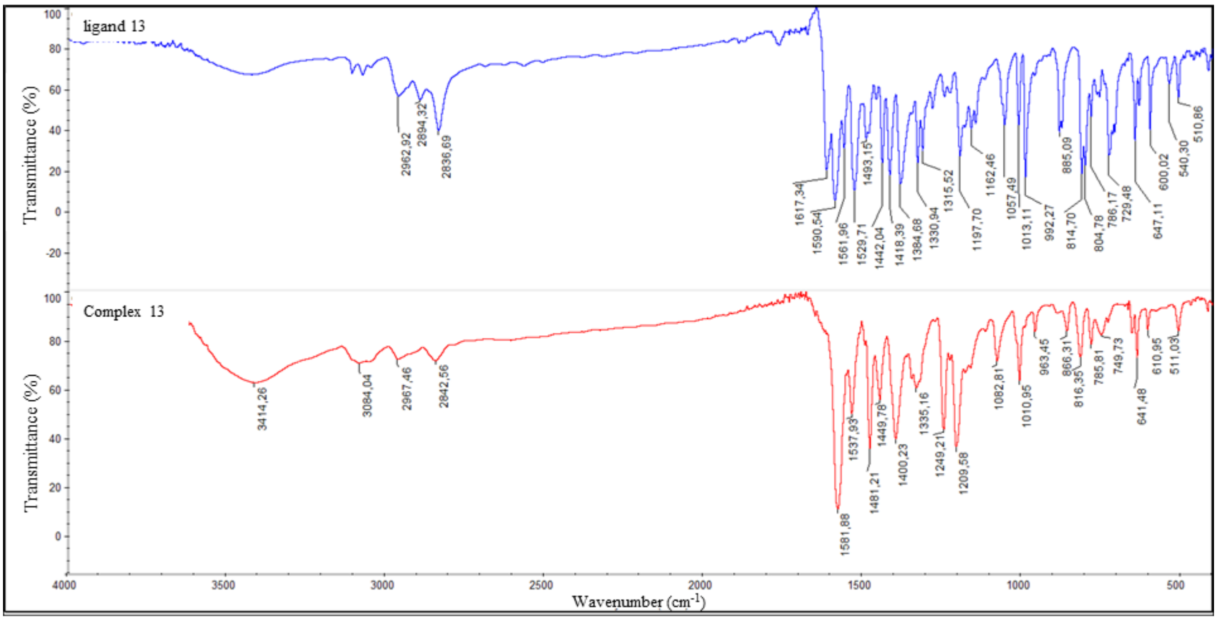
ligand 13
Complex 13
Transmittance (%)
Wavenumber (cm-1)
2962,92
2894,32
2836,69
1617,34
1590,54
1561,96
1529,71
1493,15
1442,04
1418,39
1384,68
1330,94
1315,52
1197,70
1162,46
1057,49
1013,11
992,27
885,09
814,70
804,78
786,17
729,48
647,11
600,02
540,30
510,86
3414,26
3084,04
2967,46
2842,56
1581,88
1537,93
1481,21
1449,78
1400,23
1335,16
1249,21
1209,58
1082,81
1010,95
963,45
866,31
816,35
785,81
749,73
641,48
610,95
511,03

12

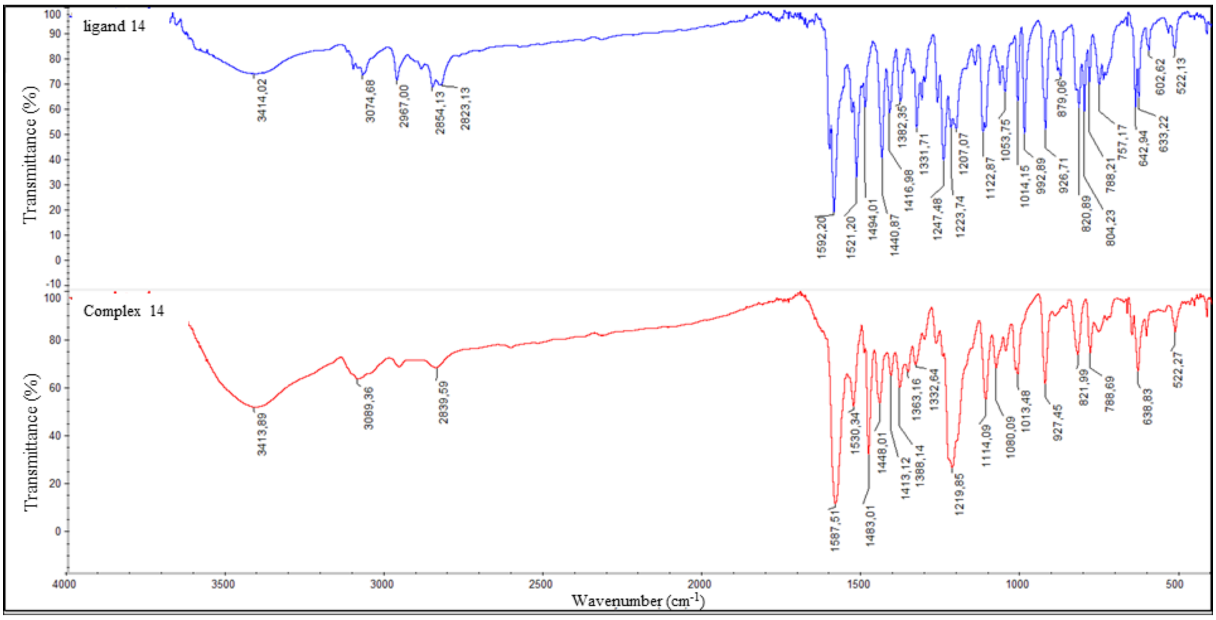
ligand 14
Complex 14
Transmittance (%)
Wavenumber (cm-1)
3414,02
3074,68
2967,00
2854,13
2823,13
1592,20
1521,20
1494,01
1440,87
1416,98
1382,35
1331,71
1247,48
1223,74
1207,07
1122,87
1053,75
1014,15
992,89
926,71
879,06
820,89
804,23
788,21
757,17
642,94
633,22
602,62
522,13
3413,89
3089,36
2839,59
1587,51
1530,34
1483,01
1448,01
1413,12
1388,14
1363,16
1332,64
1219,85
1114,09
1080,09
1013,48
927,45
821,99
788,69
638,83
522,27

13

Table S1. Comparison of $\nu_{C=N, C=C}$ [cm^{-1}] for Cu(II) complexes with terpy and dtpy.

Cuterpy	FT-IR $\nu_{C=N, C=C}$ [cm^{-1}]	Ref	terpy	FT-IR $\nu_{C=N, C=C}$ [cm^{-1}]	Ref	Cudtpy	FT-IR $\nu_{C=N, C=C}$ [cm^{-1}]	Ref	dtpy	FT-IR $\nu_{C=N, C=C}$ [cm^{-1}]	Ref
**1-Cuterpy**	1585<br>1560<br>1435	[1]	**L^{1}-terpy**	1559	[2]	**1 (lit)**	1613<br>1598<br>1563	this work and [3]	**L^{1}**	1597<br>1580<br>1565	this work
**2-Cuterpy**	1606<br>1555	[4]	**L^{2}-terpy**	1699<br>1585	[5]	**2 (lit)**	1610<br>1552	this work and [6]	**L^{2}**	1593<br>1563<br>1540	this work
**7-Cuterpy**	1602<br>1551	[7]	**L^{7}-terpy**	1586<br>1568<br>1544<br>1510	[8]	**7**	1607<br>1547	this work	**L^{7}**	1659<br>1597	this work
**8-Cuterpy**	1608<br>1569<br>1554	[7]	**L^{8}-terpy**	1587<br>1565<br>1541	[9]	**8**	1608<br>1588	this work	**L^{8}**	1597<br>1539	this work
**14-Cuterpy**	1615<br>1560	[10]	**L^{14}-terpy**	1583<br>1564<br>1545	[10]	**14**	1607<br>1552	[10]	**L^{14}**	1594<br>1542	[10]
**15-Cuterpy**	1603<br>1552	[10]	**L^{15}-terpy**	1583<br>1565<br>1541	[10]	**15**	1603<br>1545	[10]	**L^{15}**	1588<br>1540	[10]
**16-Cuterpy**	1604<br>1560	[10]	**L^{16}-terpy**	1584<br>1567	[10]	**16**	1603<br>1552	[10]	**L^{16}**	1596<br>1550	[10]
**17-Cuterpy**	1607<br>1579<br>1551	[11]	**L^{17}-terpy**	1584<br>1579<br>1551	[11]	**17**	1602<br>1575	[10]	**L^{17}**	1584<br>1530	[10]
**18-Cuterpy**	1611<br>1568<br>1552	[12]	**L^{18}-terpy**	1595<br>1583<br>1566<br>1543	[12]	**18**	1608<br>1591	[12]	**L^{18}**	1593	[12]
**19-Cuterpy**	1616<br>1603<br>1588<br>1567	[12]	**L^{19}-terpy**	1582<br>1565<br>1545	[12]	**19**	1609<br>1584<br>1567<br>1549	[12]	**L^{19}**	1601<br>1583<br>1566<br>1538	[12]
**20-Cuterpy**	1615<br>1583	[13]	**L^{20}-terpy**	1610<br>1589	[13]	**20**	1610<br>1578	[13]	**L^{20}**	1606	[13]

	1560			1565<br>1546			1550				
**21-Cuterpy**	1607<br>1569<br>1559	[13]	**L^{21}-terpy**	1599<br>1583<br>1567	[13]	**21**	1611<br>1551<br>1526	[13]	**L^{21}**	1591<br>1539	[13]
**22-Cuterpy**	1611<br>1568<br>1555	[13]	**L^{22}-terpy**	1605<br>1586<br>1568	[13]	**22**	1603<br>1599<br>1544<br>1536	[13]	**L^{22}**	1592	[13]
**23-Cuterpy**	—	[14]	**L^{23}-terpy**	1686	[14]	**23**	—	[6]	**L^{23}**	—	[6]
**24-Cuterpy**	—	—	**L^{24}-terpy**	1583<br>1567<br>1547	[15]	**24**	—	[6]	**L^{24}**	1593	[16]

X-Ray analysis

Table S2. Crystal data and structure refinement of complexes **1**–**6**.

	**1**	**2**	**3**	**4**	**5**	**6**
Empirical formula	$C_{11}H_9Cl_2N_3S_2OCu$	$C_{17}H_{11}Cl_2N_3S_2Cu$	$C_{16}H_{10}Cl_2N_4S_2Cu$	$C_{19}H_{21}Cl_2N_3S_2O_5Cu$	$C_{19}H_{15}Cl_2N_3O_2S_4Cu$	$C_{17}H_{11}Cl_2N_3OS_2Cu$
Formula weight	397.77	455.85	456.84	602.01	580.02	471.85
Temperature [K]	293.0(2)	293.0(2)	293.0(2)	293.0(2)	293.0(2)	293.0(2)
Wavelength [Å]	0.71073	0.71073	0.71073	0.71073	0.71073	0.71073
Crystal system	monoclinic	monoclinic	monoclinic	triclinic	monoclinic	triclinic
Space group	$P2_1/n$	$P2_1/n$	$P2_1/c$	$P\bar{1}$	$P2_1/c$	$P\bar{1}$
Unit cell dimensions [Å, °]	a = 8.1922(5)	a = 18.5235(10)	a = 14.1745(9)	a = 7.5457(7)	a = 9.7299(4)	a = 8.0883(5)
	b =11.1975(6)	b =8.4191(4)	b =11.9978(6)	b =12.6550(10)	b =15.0039(6)	b =10.1003(6)
	c = 15.9212(10)	c = 23.4437(12)	c = 10.4487(6)	c = 13.2542(9)	c = 15.6869(9)	c = 12.0986(7)
				α =77.635(6)		α = 65.536(5)
	β = 104.569(7)	β = 102.266(5)	β = 107.157(7)	β = 86.523(6)	β = 98.481(5)	β = 86.260(5)
				γ =80.957(7)		γ = 89.456(5)
Volume [Å ^{3}]	1413.52(15)	3572.6(3)	1697.87(19)	1220.45(17)	2265.03(19)	897.57(10)
Z	4	8	4	2	4	2
Density (calculated) [g/cm ^{3}]	1.869	1.695	1.787	1.638	1.701	1.746
Absorption coefficient [mm $^{-1}$]	2.214	1.760	1.854	1.407	1.592	1.759
F(000)	796	1832	916	614	1172	474
Crystal size [mm]	0.13 x 0.11 x 0.07	0.28 x 0.25 x 0.11	0.12 x0.09 x 0.07	0.16 x 0.13 x 0.08	0.12 x0.11 x 0.06	0.19 x 0.10 x 0.04
θ range for data collection [°]	3.64 to 25.05	3.52 to 25.05	3.39 to 25.05	3.56 to 25.05	3.83 to 25.05	3.42 to 25.05
Index ranges	$-8 \le h \le 11$	$-22 \le h \le 18$	$-13 \le h \le 16$	$-9 \le h \le 9$	$-13 \le h \le 13$	$-11 \le h \le 10$
	$-13 \le k \le 15$	$-10 \le k \le 9$	$-14 \le k \le 14$	$-15 \le k \le 13$	$-18 \le k \le 20$	$-12 \le k \le 13$
	$-20 \le l \le 20$	$-27 \le l \le 27$	$-12 \le l \le 10$	$-18 \le l \le 17$	$-15 \le l \le 21$	$-15 \le l \le 15$
Reflections collected	8503	15156	7771	11938	11828	8355
Independent reflections	3430(R_{int} = 0.0334)	6293(R_{int} =0.0268)	3001 (R_{int} = 0.0243)	5828(R_{int} = 0.0345)	5361(R_{int} =0.0258)	4201(R_{int} = 0.0321)
Completeness to 2θ [%]	99.7	99.7	99.8	99.7	99.8	99.7
Max. and min. transmission	1.00 and 0.711	1.000 and 0.639	1.00 and 0.84	1.00 and 0.709	1.000 and 0.752	1.000 and 0.447
Data / restraints / parameters	3430/ 0 /184	6293 / 0 /451	3001/ 0 / 226	5828/ 0 /324	5361 / 0 / 280	4201/ 0 / 235
Goodness-of-fit on F^2	1.066	1.032	1.064	1.042	1.021	1.108
Final R indices [$I>2\sigma(I)$]	R_1= 0.0344	R_1= 0.0324	R_1 = 0.0291	R_1= 0.0447	R_1 = 0.0455	R_1 = 0.0472
	wR_2 = 0.0729	wR_2 = 0.0755	wR_2 =0.0735	wR_2 = 0.0965	wR_2 = 0.1068	wR_2 =0.1096
R indices (all data)	R_1 = 0.0529	R_1 = 0.0478	R_1 = 0.0374	R_1 = 0.0748	R_1 =0.0764	R_1 =0.0743
	wR_2 = 0.0819	wR_2 = 0.0815	wR_2 =0.0692	wR_2 = 0.1140	wR_2 = 0.1261	wR_2 =0.1245
Largest diff. peak and hole[e Å $^{-3}$]	0.417 and -0.524	0.300 and -0.390	0.309 and -0.459	0.480 and -0.533	0.837 and -0.594	0.827and -0.450
CCDC	2500835	2500844	2500841	2500843	2500836	2500839

Table S3. Crystal data and structure refinement of complexes **7–11**.

	**7**	**8**	**9**	**10**	**11**
Empirical formula	$C_{18}H_{10}Cl_2N_4S_2Cu$	$C_{17}H_{10}BrCl_2N_3S_2Cu$	$C_{23}H_{15}Cl_2\ N_3S_2Cu$	$C_{21}H_{17}Cl_2N_5S_2Cu$	$C_{19}H_{16}Cl_2N_4S_2Cu$
Formula weight	480.86	534.75	531.94	537.96	498.92
Temperature [K]	293.0(2)	293.0(2)	293.0(2)	293.0(2)	293.0(2)
Wavelength [Å]	0.71073	0.71073	0.71073	0.71073	0.71073
Crystal system	triclinic	monoclinic	triclinic	triclinic	triclinic
Space group	$P\bar{1}$	$P2_1/c$	$P\bar{1}$	$P\bar{1}$	$P\bar{1}$
Unit cell dimensions [Å, °]	a =8.0631(5)	a = 13.3642(8)	*a =8.0603(7)*	a =7.7152(3)	a = 10.9146(5)
	b = 11.0698(10)	b =7.8994(6)	*b* = 8.9973(7)	b = 10.8824(5)	b = 13.4961(8)
	c = 12.7644(10)	c = 22.1169(10)	*c* = 15.1195(12)	c = 14.8139(7)	c = 24.4952(12)
	α =70.692(8)		α =80.145(6	α = 78.339(4)	α = 89.902(4)
	β = 85.962(6)	β = 99.082(5)	β = 84.018(7)	β = 84.515(3)	β = 84.135(4)
	γ = 69.087(7)		γ = 89.634(7)	γ = 82.548(3)	γ = 76.982(5)
Volume [$Å^3$]	1002.94(15)	2305.6(2)	1074.34(15)	1204.72(9)	3496.3(3)
Z	2	4	2	2	6
Density (calculated) [Mg/m^3]	1.592	1.541	1.644	1.483	1.422
Absorption coefficient [mm^{-1}]	1.574	3.099	1.477	1.320	1.357
F(000)	482	1052	538	546	1518
Crystal size [mm]	0.10 x 0.09 x 0.06	0.18 x 0.11 x 0.10	0.14 x 0.09 x 0.05	0.29 x 0.20 x 0.12	0.16 x 0.14 x 0.06
θ range for data collection [°]	3.34 to 25.05	3.79 to 25.05	3.98 to 25.05	4.02 to 25.05	3.39 to 25.05
Index ranges	$-9 \le h \le 9$	$-14 \le h \le 16$	$-9 \le h \le 9$	$-9 \le h \le 9$	$-13 \le h \le 12$
	$-8 \le k \le 13$	$-10 \le k \le 8$	$-9 \le k \le 10$	$-12 \le k \le 12$	$-14 \le k \le 16$
	$-15 \le l \le 15$	$-30 \le l \le 22$	$-15 \le l \le 18$	$-16 \le l \le 17$	$-29 \le l \le 26$
Reflections collected	8077	14589	9432	12497	28024
Independent reflections	3554(R_{int} = 0.0516)	5489 (R_{int} = 0.0391)	3791 (R_{int} = 0. 0593)	4242 (R_{int} = 0.0377)	12705(R_{int} = 0.0712)
Completeness to 2θ [%]	99.7	99.5	99.3	99.7	99.7
Max. and min. transmission	1.00 and 0.916	1.00 and 0.629	1.000 and	1.00 and 0.752	1.000 and 0.643
Data / restraints / parameters	3554/ 0 /244	5489 / 0 /235	3791/0/ 280	4242 / 0 / 281	12705/ 0 /763
Goodness-of-fit on F^2	1.078	1.033	0.974	1.061	1.071
Final R indices [$I>2\sigma(I)$]	R_1 = 0.0394	R_1= 0.0466	R_1 =0.0552	R_1 = 0.0379	R_1 = 0.0786
	wR_2 = 0.0976	wR_2 = 0.1065	wR_2 = 0.1252	wR_2 = 0.1005	wR_2 = 0.1796
R indices (all data)	R_1 = 0.0493	R_1 = 0.0736	R_1 = 0.0889	R_1 = 0.0489	R_1 = 0.1392
	wR_2 = 0.1024	wR_2 = 0.1182	wR_2 = 0.1431	wR_2 = 0.1052	wR_2 = 0.1996
Largest diff. peak and hole[e $Å^{-3}$]	0.366 and -0.380	0.950 and -0.691	0.583 and -0.596	0.672 and -0.466	0.643 and -0.525
CCDC	2500842	2500840	2500845	2500837	2500838

Table S4. Selected bond lengths (Å) and angles (deg) for **1**.

**Bond lengths[Å]**		**Bond angles [°]**	
Cu(1)–N(1)	2.054(2)	N(1)–Cu(1)–N(2)	78.43(8)
Cu(1)–N(2)	1.980(2)	N(1)–Cu(1)–N(3)	153.93(9)
Cu(1)–N(3)	2.050(2)	N(2)–Cu(1)–N(3)	78.15(8)
Cu(1)–Cl(1)	2.2282(7)	N(1)–Cu(1)–Cl(1)	100.21(6)
Cu(1)–Cl(2)	2.4809(8)	N(1)–Cu(1)–Cl(2)	99.79(6)
		N(2)–Cu(1)–Cl(1)	160.00(6)
		N(2)–Cu(1)–Cl(2)	95.92(6)
		N(3)–Cu(1)–Cl(1)	97.87(6)
		N(3)–Cu(1)–Cl(2)	93.86(6)
		Cl(1)–Cu(1)–Cl(2)	103.94(3)

Table S5. Selected bond lengths (Å) and angles (deg) for **2**.

**Bond lengths [Å]**		**Bond angles [°]**	
Cu(1)–N(1)	2.047(2)	N(1)–Cu(1)–N(2)	78.06(8)
Cu(1)–N(2)	1.971(2)	N(1)–Cu(1)–N(3)	154.30(9)
Cu(1)–N(3)	2.035(2)	N(2)–Cu(1)–N(3)	78.23(9)
Cu(1)-Cl(1)	2.2443(8)	N(1)–Cu(1)–Cl(1)	98.19(7)
Cu(1)-Cl(2)	2.4571(8)	N(1)–Cu(1)–Cl(2)	95.71(7)
		N(2)–Cu(1)–Cl(1)	155.02(7)
		N(2)–Cu(1)–Cl(2)	100.63(7)
		N(3)–Cu(1)–Cl(1)	99.10(7)
		N(3)–Cu(1)–Cl(2)	98.26(6)
		Cl(1)–Cu(1)–Cl(2)	104.33(3)
Cu(2)-N(4)	2.045(3)	N(4)–Cu(2)–N(5)	77.88(10)
Cu(2)-N(5)	1.979(2)	N(4)–Cu(2)–N(6)	153.67(10)
Cu(2)-N(6)	2.060(2)	N(5)–Cu(2)–N(6)	77.99(9)
Cu(2)-Cl(3)	2.2136(9)	N(4)–Cu(2)–Cl(3)	98.72(8)
Cu(2)-Cl(4)	2.4495(8)	N(4)–Cu(2)–Cl(4)	102.32(7)
		N(5)–Cu(2)–Cl(3)	158.05(7)
		N(5)–Cu(2)–Cl(4)	97.52(7)
		N(6)–Cu(2)–Cl(3)	99.70(8)
		N(6)–Cu(2)–Cl(4)	91.14(7)
		Cl(3)–Cu(2)–Cl(4)	104.37(3)

TableS6. Selected bond lengths (Å) and angles (deg) for **3**.

**Bond lengths [Å]**		**Bond angles [°]**	
Cu(1)-N(1)	2.062(2)	N(1)–Cu(1)–N(2)	78.38(8)
Cu(1)-N(2)	1.9795(19)	N(1)–Cu(1)–N(3)	154.83(8)
Cu(1)-N(3)	2.079(2)	N(2)–Cu(1)–N(3)	78.15(8)
Cu(1)-Cl(1)	2.2189(7)	N(1)–Cu(1)–Cl(1)	99.17(6)
Cu(1)-Cl(2)	2.4911(7)	N(1)–Cu(1)–Cl(2)	94.16(6)
		N(2)–Cu(1)–Cl(1)	162.27(6)
		N(2)–Cu(1)–Cl(2)	93.87(6)
		N(3)–Cu(1)–Cl(1)	100.39(6)
		N(3)–Cu(1)–Cl(2)	96.36(6)
		Cl(1)–Cu(1)–Cl(2)	103.83(3)

TableS7. Selected bond lengths (Å) and angles (deg) for **4**.

**Bond lengths [Å]**		**Bond angles [°]**	
Cu(1)-N(1)	2.041(3)	N(1)–Cu(1)–N(2)	78.53(10)
Cu(1)-N(2)	1.977(2)	N(1)–Cu(1)–N(3)	155.21(10)
Cu(1)-N(3)	2.039(2)	N(2)–Cu(1)–N(3)	78.26(10)
Cu(1)-Cl(1)	2.2240(9)	N(1)–Cu(1)–Cl(1)	98.45(8)
Cu(1)-Cl(2)	2.5211(9)	N(1)–Cu(1)–Cl(2)	96.58(8)
		N(2)–Cu(1)–Cl(1)	155.13(8)
		N(2)–Cu(1)–Cl(2)	100.12(8)
		N(3)–Cu(1)–Cl(1)	98.92(7)
		N(3)–Cu(1)–Cl(2)	98.92(7)
		Cl(1)–Cu(1)–Cl(2)	104.75(4)

Table S8. Selected bond lengths (Å) and angles (deg) for **5**.

**Bond lengths [Å]**		**Bond angles [°]**	
Cu(1)-N(1)	2.061(3)	N(1)–Cu(1)–N(2)	78.42(11)
Cu(1)-N(2)	1.966(3)	N(1)–Cu(1)–N(3)	155.52(11)
Cu(1)-N(3)	2.060(3)	N(2)–Cu(1)–N(3)	78.63(11)
Cu(1)-Cl(1)	2.2180(11)	N(1)–Cu(1)–Cl(1)	99.47(9)
Cu(1)-Cl(2)	2.5750(12)	N(1)–Cu(1)–Cl(2)	94.89(9)
		N(2)–Cu(1)–Cl(1)	162.28(9)
		N(2)–Cu(1)–Cl(2)	95.57(9)
		N(3)–Cu(1)–Cl(1)	99.78(9)
		N(3)–Cu(1)–Cl(2)	95.70(9)
		Cl(1)–Cu(1)–Cl(2)	102.15(4)

Table S9. Selected bond lengths (Å) and angles (deg) for **6**.

**Bond lengths [Å]**		**Bond angles [°]**	
Cu(1)-N(1)	2.070(3)	N(1)–Cu(1)–N(2)	78.30(11)
Cu(1)-N(2)	1.975(3)	N(1)–Cu(1)–N(3)	153.11(12)
Cu(1)-N(3)	2.050(3)	N(2)–Cu(1)–N(3)	78.10(11)
Cu(1)-Cl(1)	2.2287(11)	N(1)–Cu(1)–Cl(1)	99.29(9)
Cu(1)-Cl(2)	2.4643(11)	N(1)–Cu(1)–Cl(2)	95.70(9)
		N(2)–Cu(1)–Cl(1)	160.15(10)
		N(2)–Cu(1)–Cl(2)	96.52(9)
		N(3)–Cu(1)–Cl(1)	98.52(9)
		N(3)–Cu(1)–Cl(2)	99.61(9)
		Cl(1)–Cu(1)–Cl(2)	103.33(4)

TableS10. Selected bond lengths (Å) and angles (deg) for **7**.

**Bond lengths [Å]**		**Bond angles [°]**	
Cu(1)-N(1)	2.048(2)	N(1)–Cu(1)–N(2)	78.14(10)
Cu(1)-N(2)	1.991(3)	N(1)–Cu(1)–N(3)	156.18(11)
Cu(1)-N(3)	2.044(2)	N(2)–Cu(1)–N(3)	78.41(10)
Cu(1)-Cl(1)	2.2298(10)	N(1)–Cu(1)–Cl(1)	97.44(8)
Cu(1)-Cl(2)	2.4003(11)	N(1)–Cu(1)–Cl(2)	95.36(9)
		N(2)–Cu(1)–Cl(1)	140.71(9)
		N(2)–Cu(1)–Cl(2)	103.88(9)
		N(3)–Cu(1)–Cl(1)	98.01(8)
		N(3)–Cu(1)–Cl(2)	94.24(9)
		Cl(1)–Cu(1)–Cl(2)	115.42(4)

Table S11. Selected bond lengths (Å) and angles (deg) for **8**.

**Bond lengths [Å]**		**Bond angles [°]**	
Cu(1)-N(1)	2.048(3)	N(1)–Cu(1)–N(2)	77.96(12)
Cu(1)-N(2)	1.975(3)	N(1)–Cu(1)–N(3)	154.34(12)
Cu(1)-N(3)	2.054(3)	N(2)–Cu(1)–N(3)	78.90(12)
Cu(1)-Cl(1)	2.2216(11)	N(1)–Cu(1)–Cl(1)	99.97(9)
Cu(1)-Cl(2)	2.5317(11)	N(1)–Cu(1)–Cl(2)	93.78(9)
		N(2)–Cu(1)–Cl(1)	160.92(10)
		N(2)–Cu(1)–Cl(2)	96.64(9)
		N(3)–Cu(1)–Cl(1)	98.26(9)
		N(3)–Cu(1)–Cl(2)	99.76(9)
		Cl(1)–Cu(1)–Cl(2)	102.43(4)

Table S12. Selected bond lengths (Å) and angles (deg) for **9**.

**Bond lengths [Å]**		**Bond angles [°]**	
Cu(1)-N(1)	2.055(4)	N(1)–Cu(1)–N(2)	78.40(15)
Cu(1)-N(2)	1.976(3)	N(1)–Cu(1)–N(3)	153.50(15)
Cu(1)-N(3)	2.033(4)	N(2)–Cu(1)–N(3)	78.46(15)
Cu(1)-Cl(1)	2.2205(13)	N(1)–Cu(1)–Cl(1)	101.12(12)
Cu(1)-Cl(2)	2.5247(14)	N(1)–Cu(1)–Cl(2)	93.30(12)
		N(2)–Cu(1)–Cl(1)	160.76(13)
		N(2)–Cu(1)–Cl(2)	95.29(12)
		N(3)–Cu(1)–Cl(1)	96.50(11)
		N(3)–Cu(1)–Cl(2)	101.52(12)
		Cl(1)–Cu(1)–Cl(2)	103.94(5)

Table S13. Selected bond lengths (Å) and angles (deg) for **10**.

**Bond lengths [Å]**		**Bond angles [°]**	
Cu(1)-N(1)	2.058(2)	N(1)–Cu(1)–N(2)	77.91(9)
Cu(1)-N(2)	1.976(2)	N(1)–Cu(1)–N(3)	153.37(10)
Cu(1)-N(3)	2.044(2)	N(2)–Cu(1)–N(3)	77.78(9)
Cu(1)-Cl(1)	2.2572(9)	N(1)–Cu(1)–Cl(1)	98.17(7)
Cu(1)-Cl(2)	2.4282(8)	N(1)–Cu(1)–Cl(2)	97.32(6)
		N(2)–Cu(1)–Cl(1)	151.81(7)
		N(2)–Cu(1)–Cl(2)	103.88(7)
		N(3)–Cu(1)–Cl(1)	98.10(8)
		N(3)–Cu(1)–Cl(2)	98.90(7)
		Cl(1)–Cu(1)–Cl(2)	104.30(3)

Table S14. Selected bond lengths (Å) and angles (deg) for **11**.

**Bond lengths [Å]**		**Bond angles [°]**	
Cu(1)-N(1)	2.064(6)	N(1)–Cu(1)–N(2)	78.4(2)
Cu(1)-N(2)	1.952(5)	N(1)–Cu(1)–N(3)	156.4(2)
Cu(1)-N(3)	2.045(6)	N(2)–Cu(1)–N(3)	78.4(2)
Cu(1)-Cl(1)	2.265(2)	N(1)–Cu(1)–Cl(1)	97.39(18)
Cu(1)-Cl(2)	2.4107(18)	N(1)–Cu(1)–Cl(2)	100.37(15)
		N(2)–Cu(1)–Cl(1)	142.63(16)
		N(2)–Cu(1)–Cl(2)	112.51(15)
		N(3)–Cu(1)–Cl(1)	98.33(17)
		N(3)–Cu(1)–Cl(2)	92.54(15)
		Cl(1)–Cu(1)–Cl(2)	104.80(8)
Cu(2)-N(5)	2.048(6)	N(5)–Cu(2)–N(6)	77.6(2)
Cu(2)-N(6)	1.964(5)	N(5)–Cu(2)–N(7)	155.3(2)
Cu(2)-N(7)	2.047(6)	N(6)–Cu(2)–N(7)	78.0(2)

Cu(2)-Cl(3)	2.255(2)	N(5)–Cu(2)–Cl(3)	100.72(18)
Cu(2)-Cl(4)	4312(18)	N(5)–Cu(2)–Cl(4)	99.91(15)
		N(6)–Cu(2)–Cl(3)	145.55(15)
		N(6)–Cu(2)–Cl(4)	109.02(14)
		N(7)–Cu(2)–Cl(3)	97.19(19)
		N(7)–Cu(2)–Cl(4)	91.56(16)
		Cl(3)–Cu(2)–Cl(4)	105.17(8)
Cu(3)-N(9)	2.032(6)	N(9)–Cu(3)–N(10)	78.7(2)
Cu(3)-N(10)	1.977(5)	N(9)–Cu(3)–N(11)	155.5(2)
Cu(3)-N(11)	2.054(6)	N(10)–Cu(3)–N(11)	77.2(2)
Cu(3)-Cl(5)	2.2576(19)	N(9)–Cu(3)–Cl(5)	96.93(16)
Cu(3)-Cl(6)	2.420(2)	N(5)–Cu(3)–Cl(6)	98.90(17)
		N(10)–Cu(3)–Cl(5)	143.89(15)
		N(10)–Cu(3)–Cl(6)	112.42(15)
		N(11)–Cu(3)–Cl(5)	99.66(16)
		N(11)–Cu(3)–Cl(6)	94.60(16)
		Cl(5)–Cu(3)–Cl(6)	103.68(8)

Table S15. Short intra- and intermolecular hydrogen bonds in the structure of **1**.

**D—H•••A**	**D—H**	**H•••A**	**D•••A [Å]**	**D—H•••A [°]**
O(1)–H(1A)•••Cl(1)[a]	0.85	2.61	3.362(2)	148.00
O(1)–H(1B)•••Cl(2)[b]	0.85	2.41	3.238(3)	166.00
C(1)–H(1)•••O(1)[c]	0.93	2.43	3.325(5)	161.00
C(5)–H(5)•••Cl(2)[d]	0.93	2.75	3.603(3)	153.00
C(7)–H(7)•••Cl(2)[e]	0.93	2.81	3.494(2)	131.00
C(10)–H(10)•••O(1)	0.93	2.58	3.082(4)	114.00

Symmetry code:a): *x*,-1+*y*,*z*; (b): 1/2-*x*,-1/2+*y*,3/2-*z*; (c): *x*,1+*y*,*z*; (d):1+*x*,*y*,*z*; (e): 3/2-*x*,-1/2+*y*,3/2-*z*

Table S16. Short intra- and intermolecular hydrogen bonds in the structure of **2.**

**D—H•••A**	**D—H**	**H•••A**	**D•••A [Å]**	**D—H•••A [°]**
C(2)–H(2)•••Cl(1)[a]	0.93	2.82	3.478(3)	134.00
C(2)–H(2)•••Cl(2)[a]	0.93	2.74	3.504(3)	140.00
C(7)–H(7)•••Cl(2)[b]	0.93	2.69	3.597(3)	164.00
C(13)–H(13)•••Cl(2)[b]	0.93	2.76	3.647(4)	159.00
C(19)–H(19)•••Cl(1)[c]	0.93	2.76	3.577(3)	147.00
C(27)–H(27)•••Cl(2)	0.93	2.74	3.451(4)	134.00

Symmetry code: a): 3/2-*x*,1/2+*y*,1/2-*z*; (b): 2-*x*,1-*y*,1-*z*; (c):-1/2+*x*,3/2-*y*,1/2+*z*;

Table S17. Short intra- and intermolecular hydrogen bonds in the structure of **3**.

**D—H•••A**	**D—H**	**H•••A**	**D•••A [Å]**	**D—H•••A [°]**
C(2)–H(2)•••Cl(1)[a]	0.93	2.65	3.361(3)	133.00
C(5)–H(5)•••Cl(2)[b]	0.93	2.70	3.623(2)	172.00
C(10)–H(10)•••S(1)[c]	0.93	2.87	3.761(3)	160.00

Symmetry code: a): -*x*,-1/2+*y*,1/2-z; b): *x*,1/2-*y*,1/2+*z*; c): *x*,1+*y*,*z*

Table S18. Short intra- and intermolecular hydrogen bonds in the structure of **4**.

**D—H•••A**	**D—H**	**H•••A**	**D•••A [Å]**	**D—H•••A [°]**
O(3)–H(3)•••Cl(2)	0.82	2.34	3.154(4)	174.00
O(5)–H(5A)•••O(4)[a]	0.85	1.91	2.760(6)	174.00
O(5)–H(5B)•••Cl(2)	0.85	2.46	3.263(4)	157.00
C(10)–H(10)•••O(5)[b]	0.93	2.42	3.112(3)	171.00
C(11)–H(11)•••O(2)[c]	0.93	2.42	3.340(5)	145.00
C(17)–H(17)•••O(5)[d]	0.93	2.44	3.366(5)	172.00

Symmetry code: a): -*x*,1-*y*,1-*z*; (b): -*x*,1-*y*,2-*z*; (c): -1+*x*,1+*y*,*z*; (d): 1-*x*,-*y*,2-*z*

Table S19. Short intra– and intermolecular contacts in the structure of **5**.

**D—H•••A**	**D—H**	**H•••A**	**D•••A [Å]**	**D—H•••A [°]**
O(1)–H(1B)•••Cl(2)[a]	0.79	2.72	3.479(5)	161.00
O(2)–H(2A)•••Cl(2)[b]	0.85	2.43	3.242(3)	160.00
O(2)–H(2B)•••Cl(2)[c]	0.85	2.46	3.298(3)	167.00
C(13)–H(13)•••Cl(2)[d]	0.93	2.81	3.734(4)	175.0

Symmetry code: a): -*x*,-1/2+*y*,1/2-*z*; (b): 1+*x*,*y*,*z*; (c): 1-*x*,-*y*,1-*z*; (d): -*x*,1/2+*y*,1/2-*z*

Table S20. Short intra– and intermolecular contacts in the structure of **6**.

**D—H•••A**	**D—H**	**H•••A**	**D•••A [Å]**	**D—H•••A [°]**
C(1)–H(1)•••Cl(1)[a]	0.93	2.79	3.521(4)	136.00
C(2)–H(2)•••O(1)[b]	0.93	2.60	3.435(6)	150.00
C(7)–H(7)•••Cl(2)[c]	0.93	2.80	3.725(4)	173.00
C(10)–H(10)•••Cl(2)[d]	0.93	2.63	3.516(5)	158.00
C(12)–H(12)•••O(1)	0.93	2.56	2.884(5)	101.00
C(13)–H(13)•••Cl(2)[c]	0.93	2.76	3.639(5)	158.00
C(16)–H(16)•••Cl(2)[e]	0.93	2.81	3.613(5)	144.00
C(17)–H(17)•••Cl(1)[f]	0.93	2.81	3.694(5)	158.00

Symmetry code: (a):-1-*x*,1-*y*,-*z*; (b): -*x*,1-*y*,-*z*(c): 1+*x*,*y*,*z*; (d): -*x*,-*y*,-*z*; (e): 1-*x*,-*y*,1-*z*; (f): 1+*x*,*y*,1+*z*

Table S21. Short intra- and intermolecular hydrogen bonds in the structure of **9**.

**D—H•••A**	**D—H**	**H•••A**	**D•••A [Å]**	**D—H•••A [°]**
C(1)–H(1)•••Cl(1)[a]	0.93	2.65	3.485(5)	149.0
C(10)–H(10)•••Cl(2)[b]	0.93	2.56	3.445(6)	159.0
C(17)–H(17)•••Cl(2)[c]	0.93	2.75	3.649(5)	163.0

Symmetry codes: (a): 1-*x*,1-*y*,1-z; (b): -*x*,-*y*,1-*z*; (c): -1+*x*,*y*,*z*

Table S22. Short π•••π stacking interactions in the structure of **1**.

**Cg(I)•••Cg(J)**	**Cg(I)•••Cg(J) [Å]**	**α [°]**	**β [°]**	**γ [°]**	**Cg(I)-Perp [Å]**	**Cg(J)-Perp [Å]**
Cg(4)•••Cg(5)[f]	3.9007(15)	0.64(13)	30.33	30.01	-3.3778(11)	-3.3667(10)
Cg(5)•••Cg(3)[g]	3.7615(15)	7.68(13)	27.52	20.46	3.5241(10)	3.3360(11)

*α = dihedral angle between*Cg*(I) and*Cg*(J); *Cg*(I)-Perp = Perpendicular distance of*Cg*(I) on ring J;*Cg*(J)-Perp = perpendicular distance of*Cg*(J) on ring I; β = angle*Cg*(I)→*Cg*(J) vector and normal to ring I; γ = angle*Cg*(I) →*Cg*(J) vector and normal to plane J;
Cg(3): S(1)/C(2)/C(1)/N(1)/C(3)
Cg(4): S(2)/C(9)/N(3)/C(11)/C(10)
Cg(5): N(2)/C(4)/C(5)/C(6)/C(7)/C(8)
[&]Symmetry code: (b):1-*x*,1-*y*,1-*z*; (c): 3/2-*x*,-1/2+*y*,3/2-*z*

Table S23. Short π•••π stacking interactions in the structure of **2**.

**Cg(I)•••Cg(J)**	**Cg(I)•••Cg(J) [Å]**	**α [°]**	**β [°]**	**γ [°]**	**Cg(I)-Perp [Å]**	**Cg(J)-Perp [Å]**
Cg(3)•••Cg(10)[d]	3.9106(17)	19.01(15)	17.29	30.21	3.3795(11)	-3.7338(12)

*α = dihedral angle between*Cg*(I) and*Cg*(J); *Cg*(I)-Perp = Perpendicular distance of*Cg*(I) on ring J;*Cg*(J)-Perp = perpendicular distance of*Cg*(J) on ring I; β = angle*Cg*(I)→*Cg*(J) vector and normal to ring I; γ = angle*Cg*(I) →*Cg*(J) vector and normal to plane J;
Cg(3): S(1)/C(2)/C(1)/N(1)/C(3)
Cg(10):S(4)/C(26)/N(6)/C(28)/C(27)
[&]Symmetry code: (d): *x*, *y*, *z*

Table S24. Short π•••π stacking interactions in the structure of **4**.

**Cg(I)•••Cg(J)**	**Cg(I)•••Cg(J) [Å]**	**α [°]**	**β [°]**	**γ [°]**	**Cg(I)-Perp [Å]**	**Cg(J)-Perp [Å]**
Cg(3)•••Cg(3)[e]	3.8131(19) Å	0	16.45	16.45	-3.6621(15)	-3.6619(15)
Cg(4)•••Cg(4)[f]	3.5277(17)	0	17.88	17.88	-3.3547(14)	-3.3547(14)
Cg(5)•••Cg(5)[g]	3.8215(19)	0	21.77	21.77	-3.5923(15)	-3.5924(15)

*α = dihedral angle between*Cg*(I) and*Cg*(J); *Cg*(I)-Perp = Perpendicular distance of*Cg*(I) on ring J;*Cg*(J)-Perp = perpendicular distance of*Cg*(J) on ring I; β = angle*Cg*(I)→*Cg*(J) vector and normal to ring I; γ = angle*Cg*(I) →*Cg*(J) vector and normal to plane J;
Cg(3): S(1)/C(2)/C(1)/N(1)/C(3)
Cg(4): S(2)/C(9)/N(3)/C(11)/C(10)
Cg(5): S(3)/C(12)/C(13)/C(16)/C(17)
[&]Symmetry code: (e): 1-*x*,1-*y*,1-*z*; (f): -*x*,1-*y*,2-*z*; (g): 2-*x*,-*y*,2-*z*

Table S25. Short π•••π stacking interactions in the structure of **5**.

**Cg(I)•••Cg(J)**	**Cg(I)•••Cg(J) [Å]**	**α [°]**	**β [°]**	**γ [°]**	**Cg(I)-Perp [Å]**	**Cg(J)-Perp [Å]**
Cg(4)•••Cg(5)[e]	3.516(2)	1.71(18)	9.48	10.72	-3.4547(14)	-3.4679(16)

*α = dihedral angle between*Cg*(I) and*Cg*(J); *Cg*(I)-Perp = Perpendicular distance of*Cg*(I) on ring J;*Cg*(J)-Perp = perpendicular distance of*Cg*(J) on ring I; β = angle*Cg*(I)→*Cg*(J) vector and normal to ring I; γ = angle*Cg*(I) →*Cg*(J) *vector* and normal to plane J;
Cg(4): S(2)/C(9)/N(3)/C(11)/C(10)
Cg(5): S(3)/C(12)/C(13)/C(14)/C(15)
Symmetry code: (e): 1-*x*,-1/2+*y*,1/2-*z*

Table S26. Short π•••π stacking interactions in **7**.

Cg(I)•••Cg(J)	Cg(I)•••Cg(J) [Å]	α [°]	β [°]	γ [°]	Cg(I)-Perp [Å]	Cg(J)-Perp [Å]
Cg(3)•••Cg(4)^{e}	3.8023	4.886	21.46	19.56	-3.5829	3.5388

*α = dihedral angle between*Cg*(I) and*Cg*(J); *Cg*(I)-Perp = Perpendicular distance of*Cg*(I) on ring J;*Cg*(J)-Perp = perpendicular distance of*Cg*(J) on ring I; β = angle*Cg*(I)→*Cg*(J) vector and normal to ring I; γ = angle*Cg*(I) →*Cg*(J) vector and normal to plane J;
Cg(3): S(1)/C(2)/C(1)/N(1)/C(3)
Cg(4): S(2)/C(9)/N(3)/C(11)/C(10)
Symmetry code: (e) = 1+*x*,*y*,*z*

Table S27. Short π•••π stacking interactions in **8**.

Cg(I)•••Cg(J)	Cg(I)•••Cg(J) [Å]	α [°]	β [°]	γ [°]	Cg(I)-Perp [Å]	Cg(J)-Perp [Å]
Cg(3)•••Cg(4)^{b}	3.838(2)	7.78(19)	23.12	29.37	-3.3447(15)	-3.5296(15)
Cg(6)•••Cg(6)^{c}	3.734(2) Å	0	22.04	22.04	3.4615(16)	3.4615(16)

*α = dihedral angle between*Cg*(I) and*Cg*(J); *Cg*(I)-Perp = Perpendicular distance of*Cg*(I) on ring J;*Cg*(J)-Perp = perpendicular distance of*Cg*(J) on ring I; β = angle*Cg*(I)→*Cg*(J) vector and normal to ring I; γ = angle*Cg*(I) →*Cg*(J) vector and normal to plane J;
Cg(3): S(1)/C(2)/C(1)/N(1)/C(3)
Cg(4): S(2)/C(9)/N(3)/C(11)/C(10)
Cg(6): C(12)/C(13)/C(14)/C(15)/C(16)/C(17)
Symmetrycode: (b):-*x*,1-*y*,1-*z*; (c): 1-*x*,2-*y*,1-*z*

Table S28. Short π•••π stacking interactions in the structure of **9**.

Cg(I)•••Cg(J)	Cg(I)•••Cg(J) [Å]	α [°]	β [°]	γ [°]	Cg(I)-Perp [Å]	Cg(J)-Perp [Å]
Cg(3)•••Cg(4)^{d}	3.807(3) Å	10.7(3)	24.25	30.50	3.280(2)	3.4710(19)
Cg(3)•••Cg(6)^{e}	3.861(3)	5.2(3)	24.44	29.47	3.362(2)	-3.515(2)

*α = dihedral angle between*Cg*(I) and*Cg*(J); *Cg*(I)-Perp = Perpendicular distance of*Cg*(I) on ring J;*Cg*(J)-Perp = perpendicular distance of*Cg*(J) on ring I; β = angle*Cg*(I)→*Cg*(J) vector and normal to ring I; γ = angle*Cg*(I) →*Cg*(J) vector and normal to plane J;
Cg(3): S(1)/C(2)/C(1)/N(1)/C(3)
Cg(4): S(2)/C(9)/N(3)/C(11)/C(10)
Cg(6): C(12)/C(13)/C(14)/C(15)/C(16)/C(17)
$^{\&}$Symmetry code: (d) =-*x*,1-*y*,1-*z*; (e): 1+*x*,*y*,*z*

Table S29. Short π•••π stacking interactions in the structure of **10**.

Cg(I)•••Cg(J)	Cg(I)•••Cg(J) [Å]	α [°]	β [°]	γ [°]	Cg(I)-Perp [Å]	Cg(J)-Perp [Å]
Cg(3)•••Cg(3)^{b}	3.5634(15) Å]	0	18.85	18.85	3.3723(11)	3.3723(11)

*α = dihedral angle between*Cg*(I) and*Cg*(J); *Cg*(I)-Perp = Perpendicular distance of*Cg*(I) on ring J;*Cg*(J)-Perp = perpendicular distance of*Cg*(J) on ring I; β = angle*Cg*(I)→*Cg*(J) vector and normal to ring I; γ = angle*Cg*(I) →*Cg*(J) vector and normal to plane J;
Cg(3): S(1)/C(2)/C(1)/N(1)/C(3)
Symmetrycode: (b): 1-*x*,-*y*,1-*z*

Table S30. Short π•••π stacking interactions in the structure of **11**.

Cg(I)•••Cg(J)	Cg(I)•••Cg(J)	α [°]	β [°]	γ [°]	Cg(I)-Perp [Å]	Cg(J)-Perp [Å]

	**[Å]**					
Cg(1)•••Cg(6)[b]	3.598(4)	8.0(4)	5.04	7.98	3.564(3)	3.584(3)
Cg(2)•••Cg(4)[a]	3.739(4)	2.9(4)	21.76	24.60	-3.399(3)	-3.473(3)
Cg(3)•••Cg(5)[b]	3.662(4)	1.3(3)	14.00	12.92	3.569(3)	3.553(3)

*α = dihedral angle between*Cg*(I) and*Cg*(J); *Cg*(I)-Perp = Perpendicular distance of*Cg*(I) on ring J;*Cg*(J)-Perp = perpendicular distance of*Cg*(J) on ring I; β = angle*Cg*(I)→*Cg*(J) vector and normal to ring I; γ = angle*Cg*(I) →*Cg*(J) vector and normal to plane J;

Cg(1): S(1)/C(2)/C(1)/N(3)/C(3)

Cg(2): S(2)/C(9)/N(1)/C(11)/C(10)

Cg(3): N(2)/C(4)/C(5)/C(6)/C(7)/C(8)

Cg(4): C(31)/C(32)/C(33)/C(34)/C(35)/C(36)

Cg(5): N(10)/C(42)/C(43)/C(44)/C(45)/C(46)

Cg(6): C(50)/C(51)/C(52)/C(53)/C(54)/C(55)

Symmetry code: (a):1-*x*,1-*y*,1-*z*; (b):2-*x*,1-*y*,1-*z*

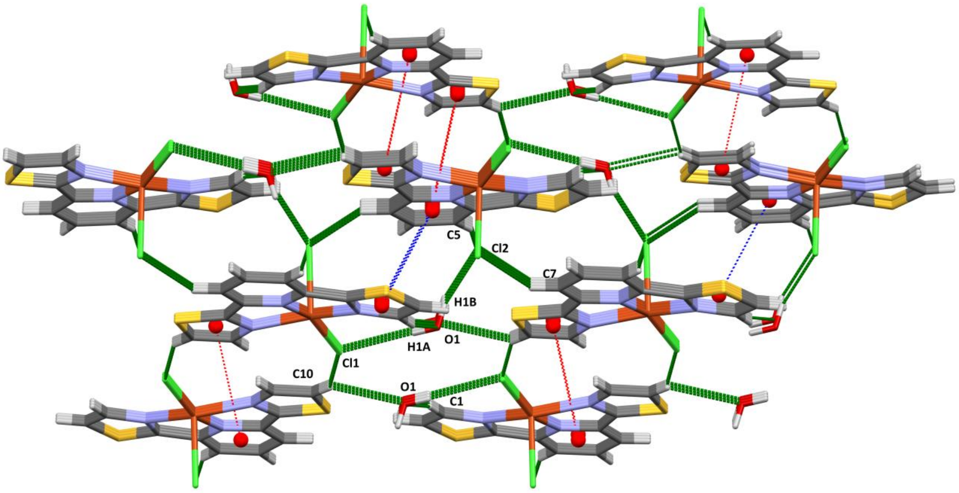

Figure S3. View of the intermolecular interactions and crystal packing arrangement for **1**.

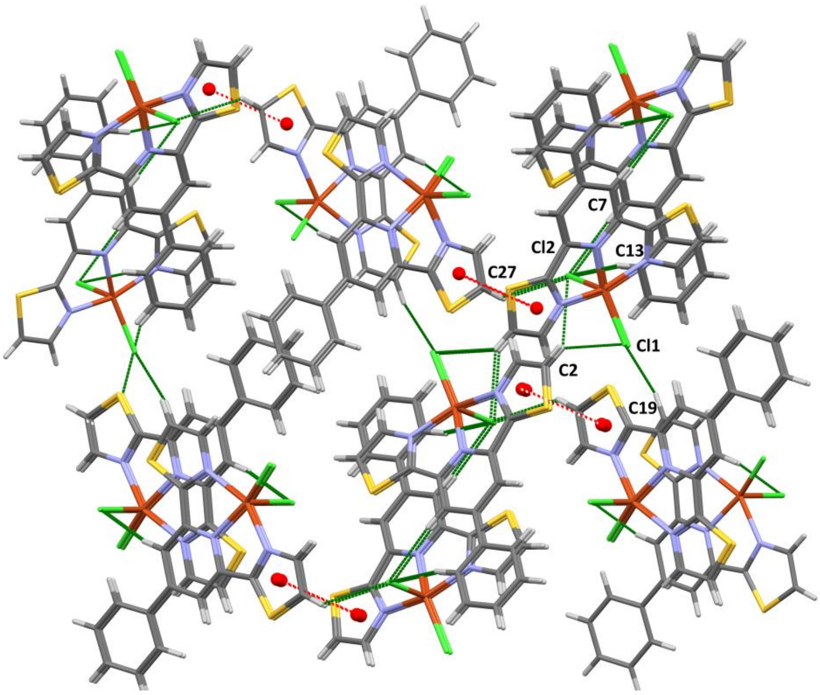

Figure S4. View of the intermolecular interactions and crystal packingarrangement for **2**.

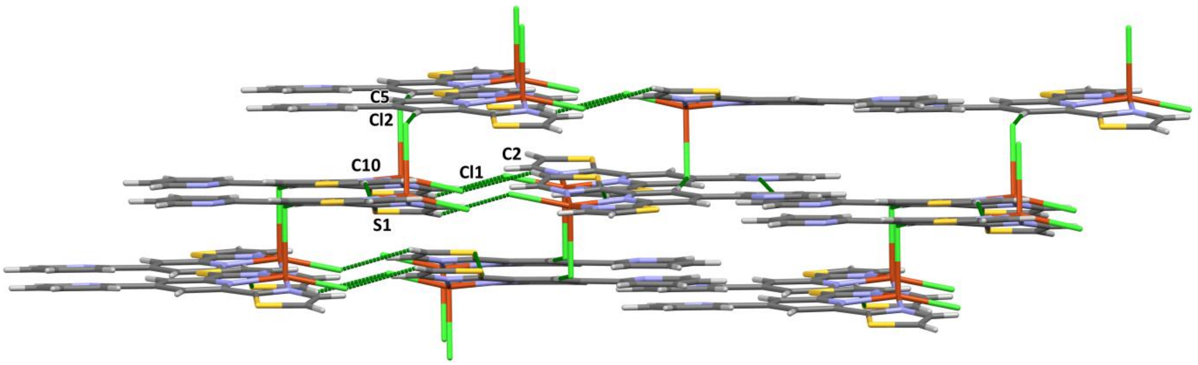

Figure S5. View of the intermolecular interactions and crystal packingarrangement for **3**.

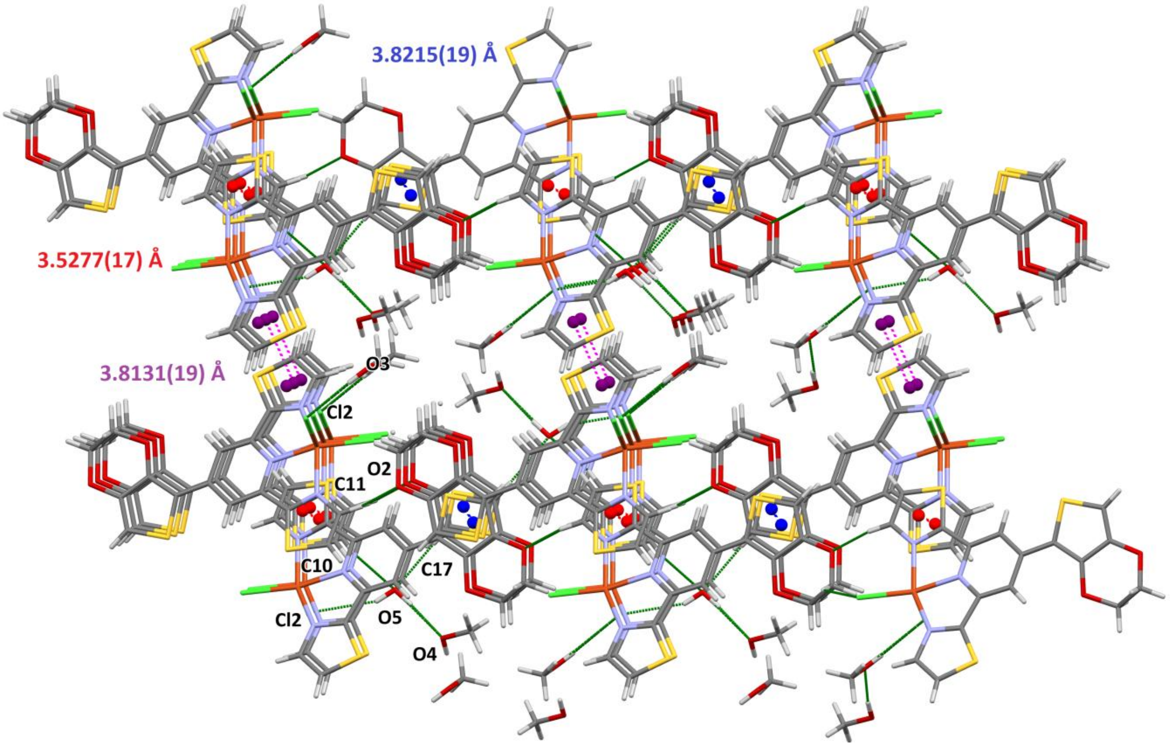

Figure S6. View of the intermolecular interactions and crystal packing arrangement for **4**.

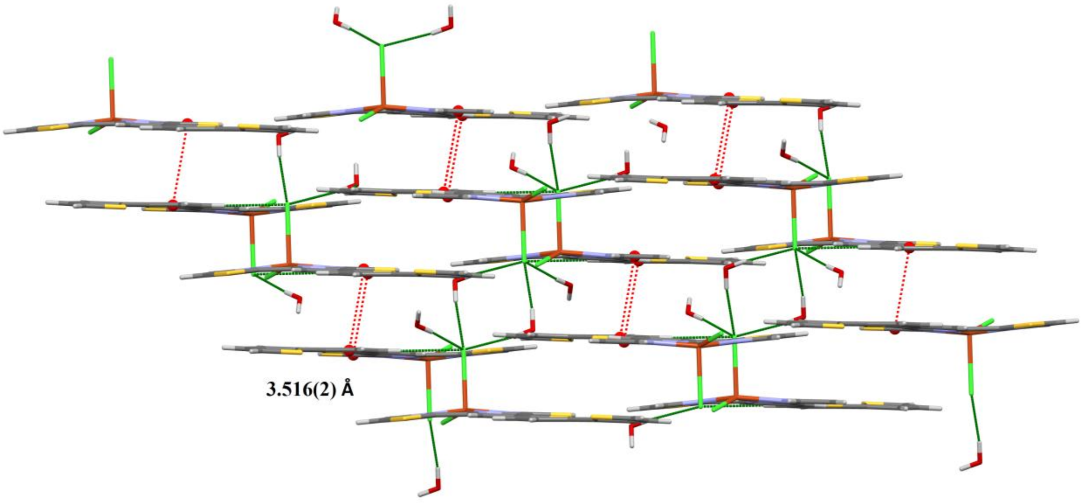

Figure S7. View of the intermolecular interactions and crystal packing arrangement for **5**.

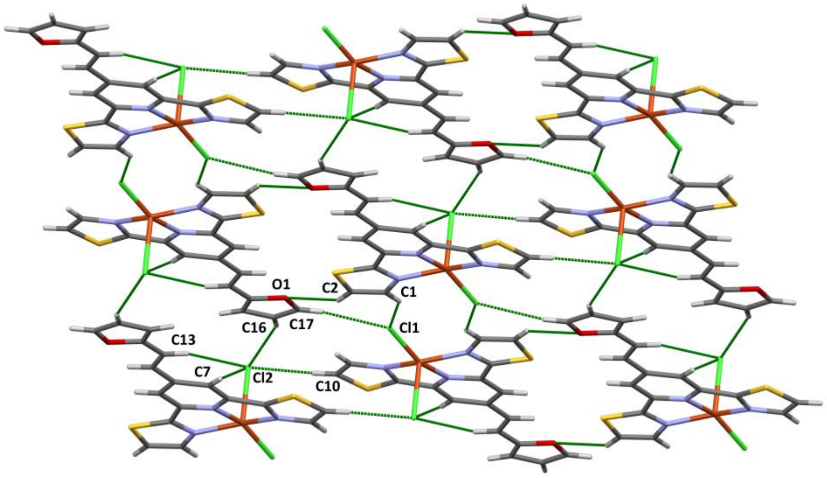

Figure S8. View of the intermolecular interactions and crystal packing arrangement for **6**.

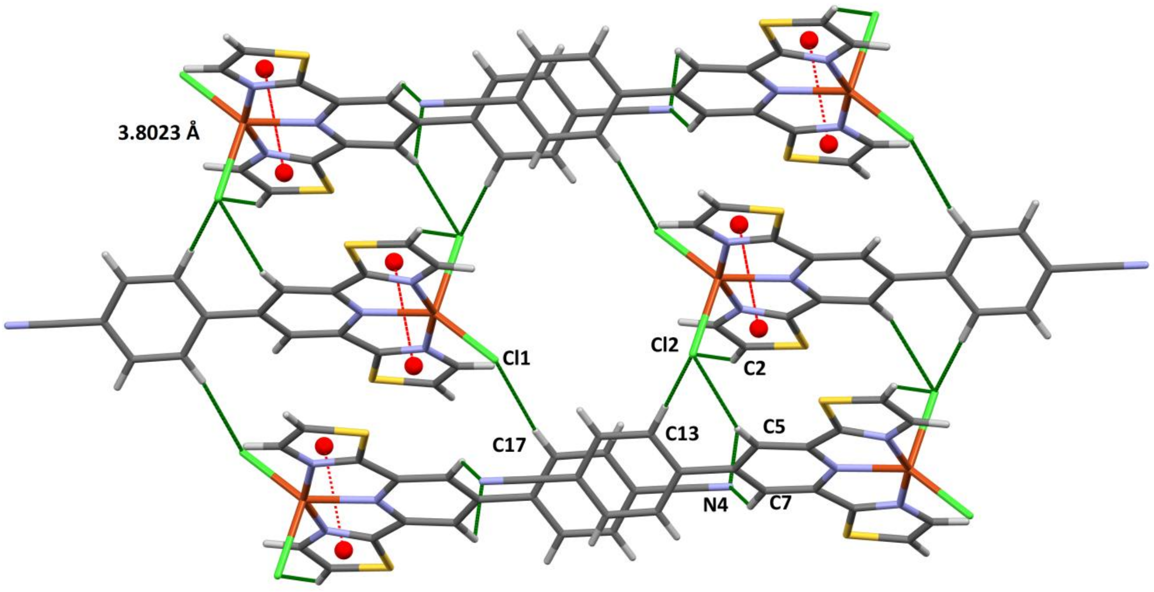

Figure S9. View of the intermolecular interactions and crystal packing arrangement for **7**.

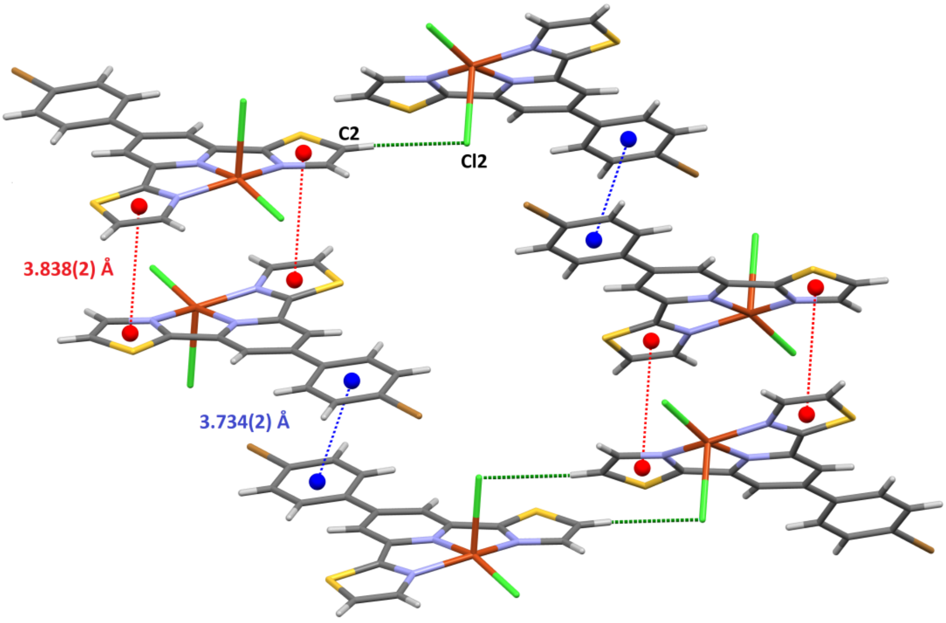

Figure S10. View of the intermolecular interactions and crystal packing arrangement for **8**.

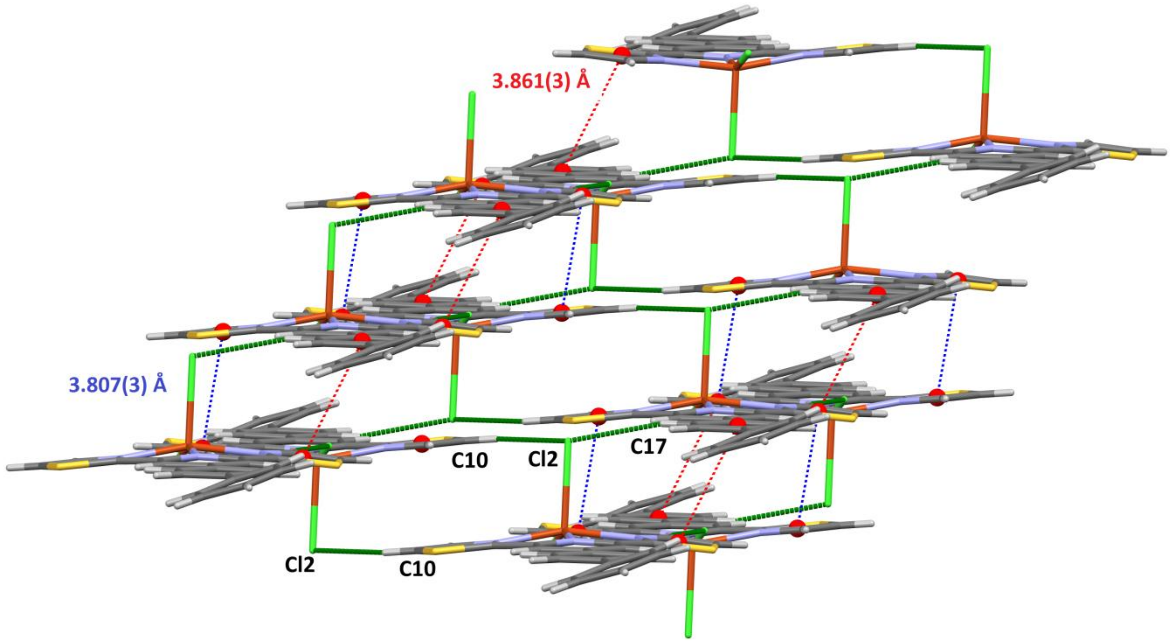

Figure S11. View of the intermolecular interactions and crystal packing arrangement for **9**.

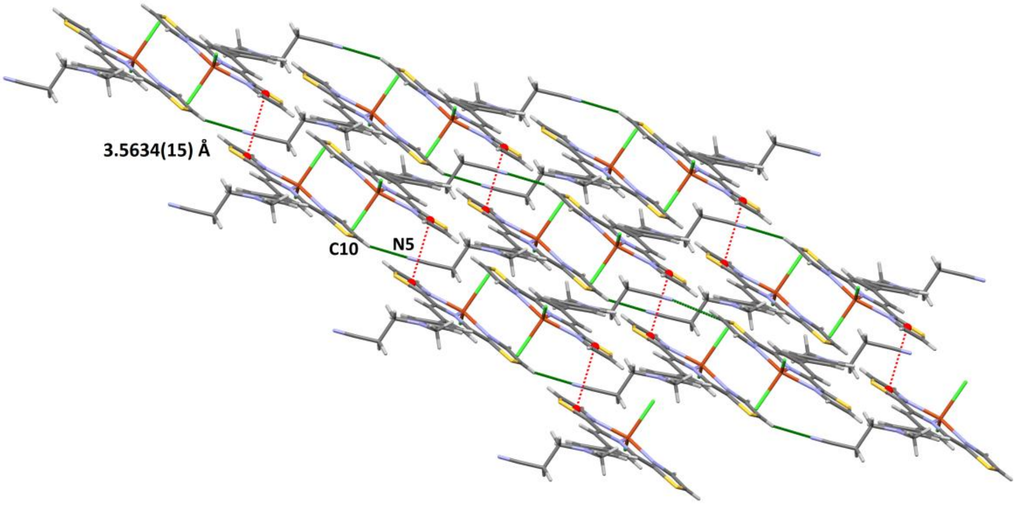

Figure S12. View of the intermolecular interactions and crystal packing arrangement for **10**.

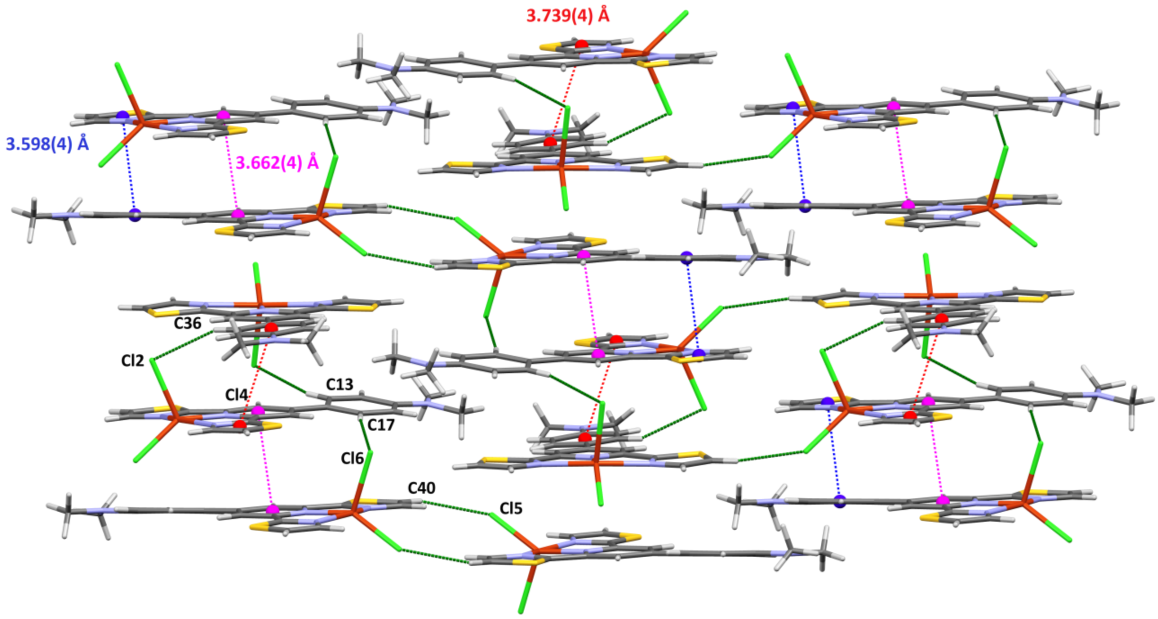

Figure S13. View of the intermolecular interactions and crystal packing arrangement for **11**.

Table S31. Structural features of Cu(II) compounds based on 2,6-bis(thiazol-2-yl)pyridines (dtpy) derivatives

No	Refcode	Substituent	Compound	Cu–Cl [Å]	<R-dtpy	d_{Cu}	Addison parametr τ	S_Q(SPY)	S_Q(TBY)	DevPath	Ref
**1**	NIDQAU	–H	[$CuCl_2$(dtpy)]	2.4460(8)<br>2.2494(7)	–	0.190	0.016	1.724	4.914	51.7	[3]
**2**	PIBWUU	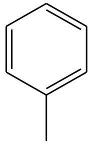	[$CuCl_2$(Phdtpy)]	**Cu1**<br>2.466(1)<br>2.252(1)<br>**Cu2**<br>2.460(1)<br>2.222(1)	**Cu1**<br>6.79<br><br>**Cu2**<br>8.77	**Cu1**<br>0.254<br><br>**Cu2**<br>0.237	**Cu1**<br>0.01<br><br>**Cu2**<br>0.07	**Cu1**<br>1.643<br><br>**Cu2**<br>1.598	**Cu1**<br>5.695<br><br>**Cu2**<br>5.200	**Cu1**<br>57.8<br><br>**Cu2**<br>52.4	[6]
**14**	SUCPEP	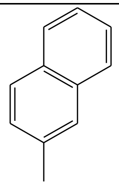	[$CuCl_2$(2-naphtdtpy)]	**Cu1**<br>2.5280(9)<br>2.214(1)<br>**Cu2**<br>2.521(1)<br>2.221(1)	**Cu1**<br>10.47<br>**Cu2**<br>1.67	**Cu1**<br>0.273<br>**Cu2**<br>0.196	**Cu1**<br>0.089<br>**Cu2**<br>0.046	**Cu1**<br>1.654<br>**Cu2**<br>1.673	**Cu1**<br>5.783<br>**Cu2**<br>5.240	**Cu1**<br>58.8<br>**Cu2**<br>54.0	[10]
**15**	SUCPAL	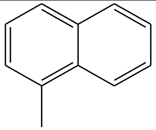	[$CuCl_2$(1-naphtdtpy)]	**Cu1**<br>2.494(2)<br>2.202(2)<br>**Cu2**<br>2.506(2)<br>2.205(2)	**Cu1**<br>60.29<br>**Cu2**<br>73.20	**Cu1**<br>0.302<br>**Cu2**<br>0.293	**Cu1**<br>0.165<br>**Cu2**<br>0.16	**Cu1**<br>1.478<br>**Cu2**<br>1.560	**Cu1**<br>6.577<br>**Cu2**<br>6.663	**Cu1**<br>62.8<br>**Cu2**<br>65.0	[10]
**16**	SUCPIT	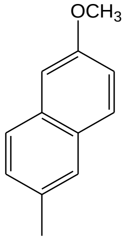	[$CuCl_2$(6-MeOnaphtdtpy)]	2.522(1)<br>2.208(1)	6.61	0.257	0.125	1.631	6.151	61.7	[10]
**17**	TUNSEE	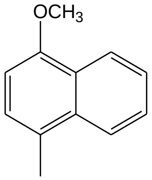	[$CuCl_2$(4-MeOnaphtdtpy)]	**Cu1**<br>2.437(2)<br>2.244(1)<br>**Cu2**<br>2.436(2)<br>2.241(1)	**Cu1**<br>40.13<br>**Cu2**<br>36.63	**Cu1**<br>0.257<br>**Cu2**<br>0.252	**Cu1**<br>0.025<br>**Cu2**<br>0.00	1.530	5.335	52.5	[10]

**18**	CONXIP	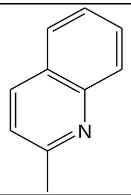	$[CuCl_2(2\text{-quindtpy})]$	2.497(1)<br>2.219(1)	12.13	0.250	0.14	1.545	5.770	56.8	[12]
**19**	CONXOV	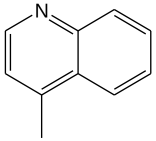	$[CuCl_2(4\text{-quin-dtpy})]\cdot 3H_2O$	2.433(2)<br>2.229(1)	48.73	0.237	0.003	1.537	4.944	48.8	[12]
**20**	FECSIS	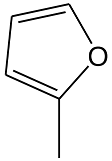	$[CuCl_2(4\text{-furantpy})]$	2.478(1)<br>2.2347(8)	2.92	0.313	0.08	1.466	5.866	56.3	[13]
**21**	FECSUE	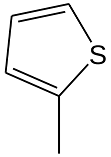	$[CuCl_2(4\text{-thiophendtpy})]$	2.464(1)<br>2.2392(9)	3.16	0.320	0.06	1.491	5.617	54.5	[13]
**22**	FECTAL	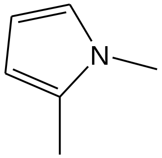	$[CuCl_2(4\text{-Mepyrrdtpy})]$	2.580(1)<br>2.231(2)	10.34	0.134	0.12	1.886	5.929	63.9	[13]
**23**	PIBXAB	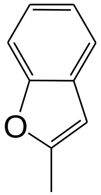	$[CuCl_2(\text{benzfurandtpy})]$	2.456(1)<br>2.2307(9)	9.51	0.320	0.094	1.420	6.004	56.7	[6]
**24**	PIBXEF	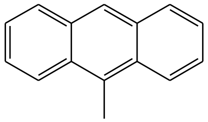	$[CuCl_2(9\text{-anthdtpy})]H_2O$	2.499(2)<br>2.220(2)	84.34	0.206	0.13	1.627	5.985	60.2	[6]

Table S32. Structural features of Cu(II) compounds based on 2,2′:6′,2″-terpyridine (*terpy*) derivatives.

No	Refcode	Substituent	Compound	Cu–Cl [Å]	<R-dtpy	d_{Cu}	Addison parametr τ	S_Q(SPY)	S_Q(TBY)	DevPath	Ref
**Cuterpy-1**	BOLPAT	—H	[$CuCl_2$terpy]·$2H_2O$	2.565<br>2.231	–	0.202	0.085	1.535	5.834	57.2	[17]
	BOLPAT01		[$CuCl_2$terpy]·H_2O	2.553<br>2.220	–	0.199	0.087	1.531	5.852	57.3	[18]
	ZZZKWM01		[$CuCl_2$terpy]	2.469<br>2.252	–	0.242	0.012	1.637	5.195	53.0	[18]
	ZZZKWM02		[$CuCl_2$terpy]	2.4641(4)<br>2.2470(4)	–	0.238	0.007	1.569	5.284	52.7	[19]
	ZZZKWM03		[$CuCl_2$terpy]	2.4699(3)<br>2.2557(3)	–	0.238	0.025	1.568	5.301	52.8	[20]
	ZZZKWM04		[$CuCl_2$terpy]	2.4667(4)<br>2.2538(4)	–	0.237	0.026	1.575	5.300	52.9	[21]
	ZZZKWM05		[$CuCl_2$terpy]	2.4724(8)<br>2.2526(9)	–	0.242	0.021	1.583	5.246	52.6	[22]
	ZZZKWM06		[$CuCl_2$terpy]	2.4699(6)<br>2.2496(6)	–	0.243	0.021	1.592	5.255	52.8	[23]
**Cuterpy-2**	HIQGEV	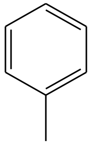	[$CuCl_2$(Phterpy)]	**Cu1**<br>2.4491(9)<br>2.2430(9)<br>**Cu2**<br>2.556(1)<br>2.247(1)	**Cu1**<br>27.53<br><br>**Cu2**<br>25.14	**Cu1**<br>0.263<br><br>**Cu2**<br>0.154	**Cu1**<br>0.047<br><br>**Cu2**<br>0.11	**Cu1**<br>1.608<br><br>**Cu2**<br>1.749	**Cu1**<br>4.657<br><br>**Cu2**<br>5.888	**Cu1**<br><br>47.2<br>**Cu2**<br>61.3	[4]
	HIQGIZ		[$CuCl_2$(Phterpy)]·$2H_2O$	2.505(2)<br>2.251(2)	8.11	0.229	0.05	1.744	4.642	49.3	[4]
	KISLUV		[$CuCl_2$(Phterpy)]	**Cu1**<br>2.4410(7)<br>2.2469(8)<br>**Cu2**<br>2.6570(9)<br>2.2343(7)	**Cu1**<br>30.06<br><br>**Cu2**<br>19.84	**Cu1**<br>0.298<br><br>**Cu2**<br>0.137	**Cu1**<br>0.00<br><br>**Cu2**<br>0.11	**Cu1**<br>1.528<br><br>**Cu2**<br>1.866	**Cu1**<br>5.110<br><br>**Cu2**<br>5.919	**Cu1**<br>50.3<br><br>**Cu2**<br>**63.5**	[24]
	AQOFAQ		[$CuCl_2$(Phterpy)]	**Cu1**<br>2.5405(6)<br>2.2565(5)<br>**Cu2**	**Cu1**<br>26.63<br><br>**Cu2**	**Cu1**<br>0.059<br><br>**Cu2**	**Cu1**<br>0.10<br><br>**Cu2**	**Cu1**<br>1.792<br><br>**Cu2**	**Cu1**<br>5.740<br><br>**Cu2**	**Cu1**<br>60.7<br><br>**Cu2**	[25]

				2.4509(6)<br>2.2534(7)	34.59	0.076	0.07	1.822	4.340	47.4	
	AQOFAQ01		No data available								[26]
**Cuterpy-3**	OGOSUZ	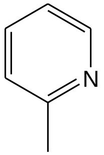	[$CuCl_2$(*meta*-pyterpy)]H_2O	**Cu1**<br>2.5219(5)<br>2.2524(6)<br>**Cu2**<br>2.4433(5)<br>2.2623(5)	**Cu1**<br>4.82<br><br>**Cu2**<br>9.45	**Cu1**<br>0.248<br><br>**Cu2**<br>0.168	**Cu1**<br>0.064<br><br>**Cu2**<br>0.035	**Cu1**<br>5.810<br><br>**Cu2**<br>1.665	**Cu1**<br>1.542<br><br>**Cu2**<br>4.802	**Cu1**<br>57.1<br><br>**Cu2**<br>49.6	[27,28]
	OGOSUZ01		[$CuCl_2$(*meta*-pyterpy)]H_2O	2.460(1)<br>2.247(1)	25.59	0.201	0.04	1.626	4.714	48.1	
**Cuterpy-7**	ETUBON	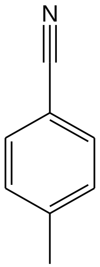	[$CuCl_2$(CN-Phterpy)]	2.5024(8)<br>2.231(1)	13.56	0.339	0.072	5.744	1.606	57.6	[7]
**Cuterpy-8**	LAJCAE	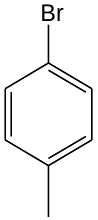	[$CuCl_2$(Br-Phterpy)]	2.4642(5)<br>2.2534(6)	-	0.168	0.27	4.830	1.600	48.8	[29]
**Cuterpy-11**	BOTXIT	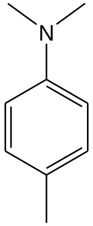	[$CuCl_2$(Me_2NPhterpy)	2.4851(6)<br>2.4851(6)	3.12	0.251	0.02	1.656	4.893	50.4	[30]

**Cuterpy-14**	SUDSUJ	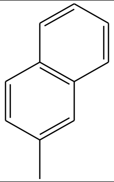	[$CuCl_2$(2-naphtterpy)]	2.4919(8) 2.2196(9)	3.72	0.328	0.12	1.589	6.080	60.4	[10]
**Cuterpy-15**	SUCNOX	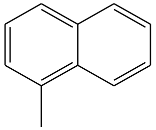	[$CuCl_2$(1-naphtterpy)]·2CH_3OH	2.642(2) 2.221(2)	48.45	0.155	0.18	1.971	6.539	70.6	[10]
**Cuterpy-16**	SUCNUD	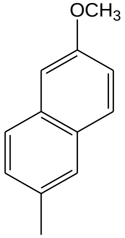	[$CuCl_2$(6-MeOnaphtterpy)]	2.6064(8) 2.2159(9)	1.22	0.338	0.22	1.835	6.682	69.7	[10]
**Cuterpy-17**	HOVNUE	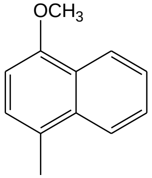	[$CuCl_2$(4-MeOnaphtterpy)]	2.5024(9) 2.230(1)	53.34	0.183	0.32	1.594	5.350	53.7	[11]
**Cuterpy-18**	CONXAH	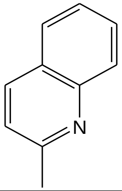	[$CuCl_2$(2-quinterpy)]	2.5299(7) 2.2254(8)	10.97	0.197	0.093	1.673	5.811	59.4	[12]
**Cuterpy-19**	CONXEL	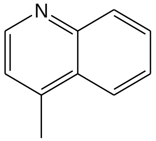	[$CuCl_2$(4-quinterpy)]	2.505(1) 2.229(2)	55.18	0.182	0.056	1.807	5.583	59.4	[12]

**Cuterpy-20**	FECSAK	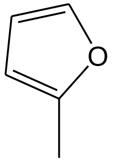	[$CuCl_2$(4-furanterpy)]	2.6006(9)<br>2.2176(9)	3.76	0.245	0.132	1.625	6.293	62.9	[13]
**Cuterpy-21**	FECSOY	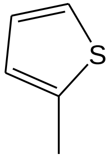	[$CuCl_2$(4-thiophenterpy)]	2.563(1)<br>2.227(1)	3.01	0.237	0.07	1.713	5.697	59.0	[13]
**Cuterpy-22**	FECSEO	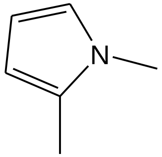	[$CuCl_2$(4-Mepyrrterpy)]	**Cu1**<br>2.4894(8)<br>2.2425(8)<br>**Cu2**<br>2.4008(9)<br>2.304(1)	**Cu1**<br>17.39<br><br>**Cu2**<br>11.79	**Cu1**<br>0.225<br><br>**Cu2**<br>0.013	**Cu1**<br>0.077<br><br>**Cu2**<br>0.56	**Cu1**<br>1.610<br><br>**Cu2**<br>3.940	**Cu1**<br>5.693<br><br>**Cu2**<br>2.489	**Cu1**<br>57.2<br>**Cu2**<br>**53.0**	[13]
**Cuterpy-23**	WACYIM	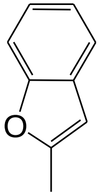	[$CuCl_2$(benzfuranterpy)]	2.4906(7)<br>2.2422(8)	6.24	0.264	0.04	1.505	5.463	53.3	[31]

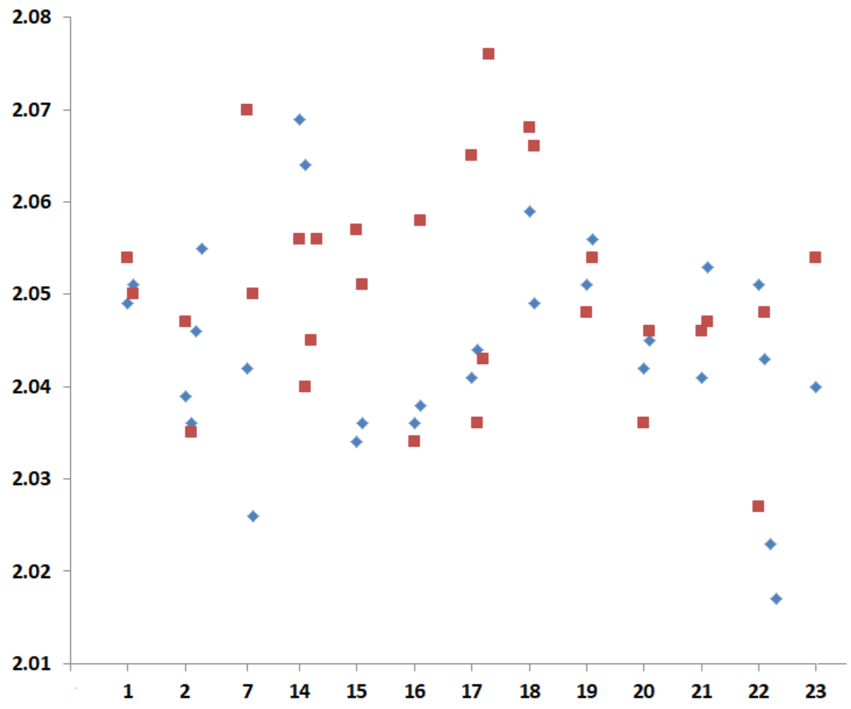

Figure S14. Comparison of Cu-N$_{peripheral}$ bond lengths in groups of copper(II) compounds constructed on *terpy* (♦) and *dtpy* (▪) derivatives

Optical properties

Table S33. Absorption band maxima for $[Cu(R\text{-}dtpy)Cl_2]$

R	σ_m	σ_p	UV-Vis band	Ref
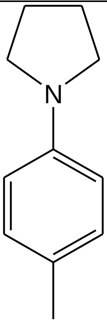 R12	-0.038	-0.155	734 (110)<br><br>465 (18300), 383 (7810), 355 (13850), 309 (24815), 281 (20500)	this work
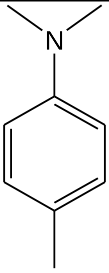 R11	-0.034	-0.147	724 (95)<br><br>456 (15565), 350 (14420), 337 (15050), 309 (23035), 280 (20440)	this work
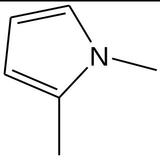 R22	-0.012	-0.101	741(110)<br><br>391(15 700),<br>338(20 700),300(22 100),274(23 300),	[13]
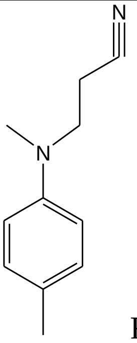 R10	0.000	-0.094	732 (65)<br><br>438 (12840), 347 (13410), 304 (19040), 278 (18135), 209 (22850)	this work
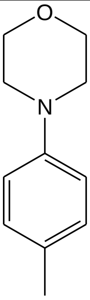 R13	0.010	-0.084	734 (95)<br><br>427 (12010), 340 (18050), 304 (24090), 278 (22770)	this work
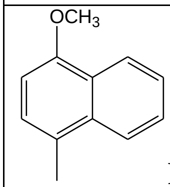 R17	0.061	-0.014	[a] 888 (88)<br><br>[b] 404, 365sh, 342, 308, 296, 277	[10]

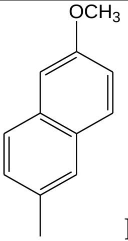 R16	0.042	-0.010	[a] 886 (77)<br><br>[b] 399, 369sh, 344, 310, 299, 278	[10]
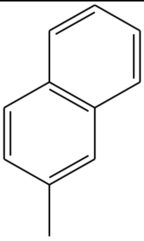 R14	0.053	0.011	742 (85)<br><br>365sh (46500), 332sh (70270), 307 (90510), 281 (93660), 231 (136600), 210 (118165)	[10]
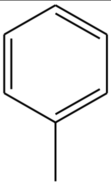 R2	0.055	0.012	742 (80),<br><br>366sh (21260), 349 (30520), 301 (75960), 203 (64240)	this work
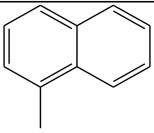 R15	0.078	0.015	[a]893 (79)<br><br>[b] 363sh, 347, 307, 273	[10]
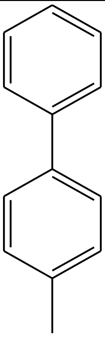 R9	0.060	0.017	739 (100)<br><br>349 (89440), 307 (97925), 263 (76290), 208 (122750)	this work
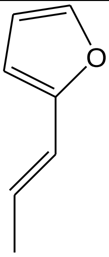 R6	0.098	0.045	746 (90)<br><br>389 (105610), 306 (77780), 276 (86300), 208 (86565)	this work
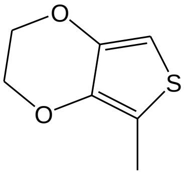 R4	0.153	0.078	737 (83)<br><br>380 (33499), 359 (36645), 335 (44282), 306 (52248), 275 (40021)	this work
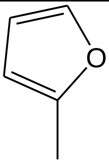 R20	0.142	0.081	741(128)<br><br>352(31 000), 308(28 100), 276(19 700)	[13]

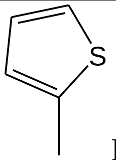 R21	0.147	0.090	736(116)<br><br>352(18 400), 304(20 800),	[13]
H R1	0.092	0.096	742 (80)<br><br>366sh (21260), 349 (30520), 301 (75960), 203 (64240)	this work
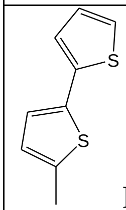 R5	0.174	0.121	727 (88)<br><br>425 (54509), 349 (48544), 309 (57361), 281 (57918), 208 (68145)	this work
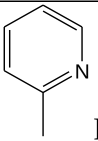 R3	0.104	0.125	760 (100)<br><br>354 (84895), 308 (68145), 271 (63030), 252 (66145), 207 (91680)	this work
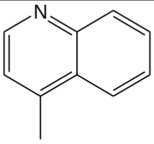 R19	0.161	0.131	745 (80)<br><br>364 (11700), 346 (15500),304 (23300),278 (24300),253 (20500),224 (42400),214 (44600),	[12]
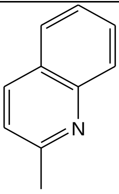 R18	0.104	0.134	748 (80)<br><br>372 (10200),333 (23800),307 (26800),282 (30300),230 (33300),213 (34700),	[12]
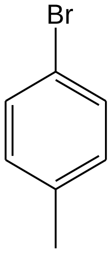 R8	0.154	0.145	742 (83)<br><br>367sh (20746), 351sh (31592), 306 (74253), 233 (40373), 207 (56524)	this work
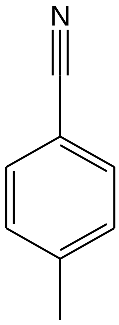 R7	0.177	0.187	742 (80)<br><br>352 (24500), 292 (84115) and 234 (44375)	this work

[a] in DMSO

[b] in EtOH

Table S34. Absorption band maxima for [Cu(R-terpy)Cl_2]

R	σ_m	σ_p	UV-Vis band	Ref
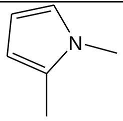 R22	-0.012	-0.101	701(115)<br><br>389(18 900),<br>320(16 600),276(21 021),266(23 900),<br>220(36 900)	[13]
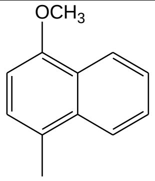 R17	0.061	-0.014	[a] 761 (1300)<br><br>[a] 369 (8500),324 (16 600),289 (16 000), 257(21 100)[a]	[10]
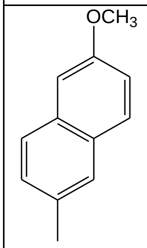 R16	0.042	-0.010	[b]713 (135)<br><br>[b]379 (17480)sh, 348 (21560), 340 (21680), 291 (33840), 274 (32200)	[11]
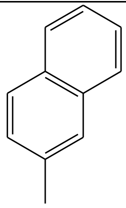 R14	0.053	0.011	[b]720 (158)<br><br>[b]344 (26560), 333 (26720), 291 (43160), 280 (39720), 267 (42640);	[10]
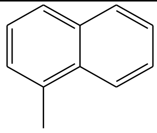 R15	0.078	0.015	[b]709 (126)<br><br>[b]350sh (17840), 327 (22720), 282 (34160), 272 (32160)	[10]
H— R1	0.092	0.096	704 (103)<br><br>339 (12630), 326 (13120), 286 (11870), 258 (17100)	[1]
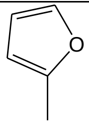 R20	0.142	0.081	701(131)<br><br>347(31 000), 289(21 300),261(23 700), 222(35 700)	[13]
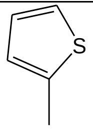 R21	0.147	0.090	701(134)<br><br>347(25 300),<br>287(21 300),266(20 700),221(31 300),	[13]
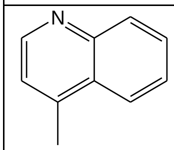 R19	0.161	0.131	703 (100)<br><br>340 (15100),328 (16500),287 (20600),263 (27100), 224 (53800), 216 (51200),	[12]

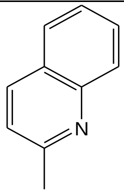 R18	0.104	0.134	*Dalton Trans.,2019*, 48, 12656	[12]

[a] in CH_3CN

[b] in DMSO

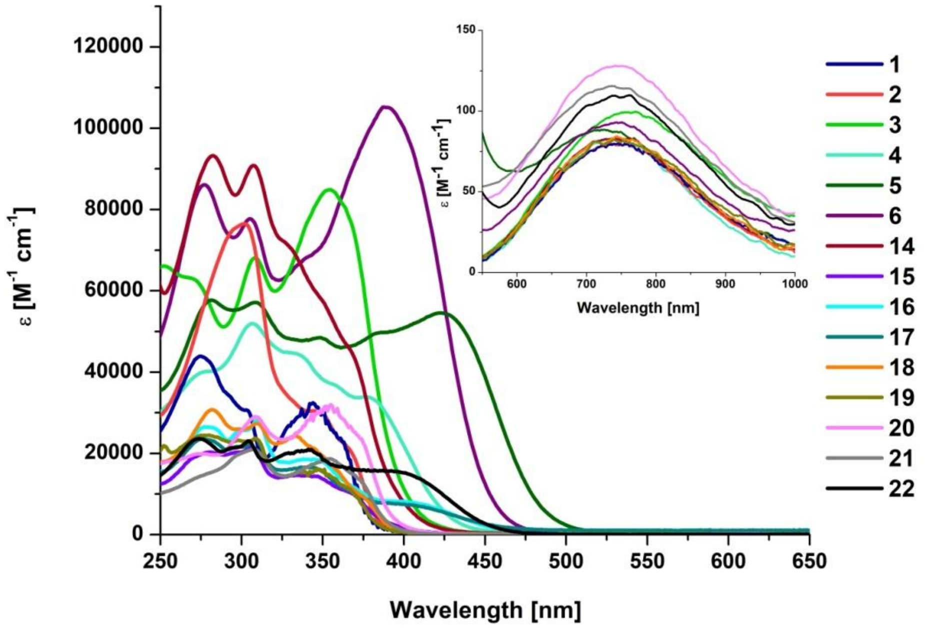

Figure S15. UV-vis spectra of **1**–**6** and **14**–**22** in diluted methanolic solutions, inset: UV-vis spectra of **1**–**6** and **18**–**22** in concentrated methanolic solutions [10, 12-13].

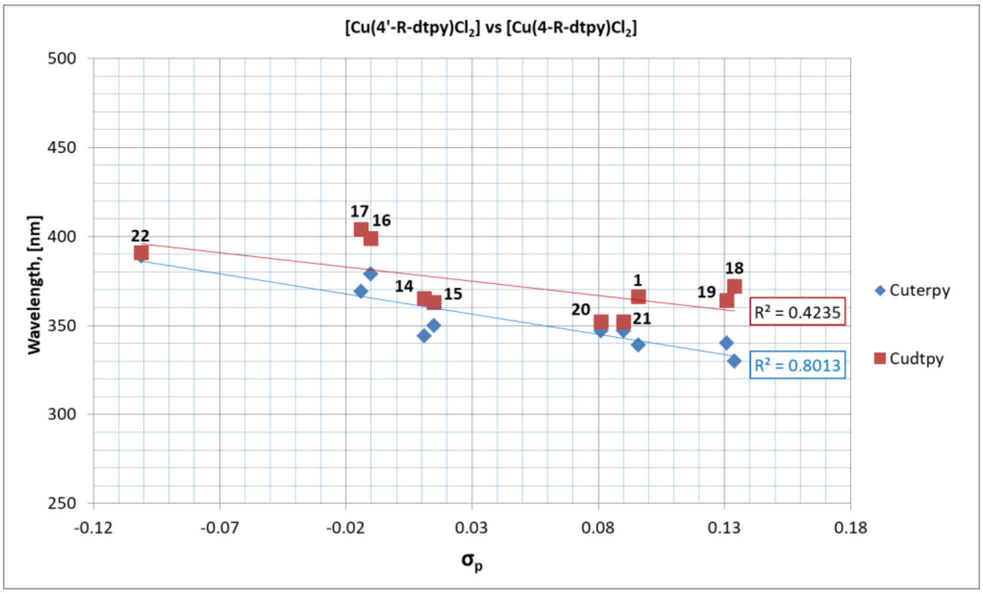

Figure S16. Wavelengths of the longest absorption bands vs σ_p Hammett's constants of substituents (R) for [Cu(4-R-dtpy)Cl_2] and [Cu(4′-R-terpy)Cl_2] in diluted methanolic solutions.

Supplementary References:

1. Hammam, A.M.; Ibrahim, S.A.; El-Gahami, M.A.; Fouad, D. Investigations of Co(II), Ni(II) and Cu(II) (2,2′:6′,2"2"-Terpyridine) Complexes With Sulfur Donor Ligands. *Journal of Thermal Analysis and Calorimetry* **2003**, *74*, 801–810, doi:10.1023/B:JTAN.0000011012.07721.e1.

2. Zhao, Q.-Q.; Ren, N.; Zhang, J.-J. Syntheses, Crystal Structures, Luminescence and Thermal Properties of Three Lanthanide Complexes with 2-Bromine-5-Methoxybenzoate and 2,2:6′,2″-Terpyridine. *Polyhedron* **2018**, *144*, 1–5, doi:10.1016/j.poly.2017.12.036.

3. Li, G.-Y.; Du, K.-J.; Wang, J.-Q.; Liang, J.-W.; Kou, J.-F.; Hou, X.-J.; Ji, L.-N.; Chao, H. Synthesis, Crystal Structure, DNA Interaction and Anticancer Activity of Tridentate Copper(II) Complexes. *Journal of Inorganic Biochemistry* **2013**, *119*, 43–53, doi:10.1016/j.jinorgbio.2012.09.019.

4. Huang, T.-H.; Zhang, M.-H.; Gao, C.-Y.; Wang, L.-T. Synthesis, Structures and Characterization of Metal Complexes Containing 4′-Phenyl-2,2′:6′,2″-Terpyridine Ligands with Extended Π···π Interactions. *Inorganica Chimica Acta* **2013**, *408*, 91–95, doi:10.1016/j.ica.2013.08.024.

5. Mondal, P.C.; Manna, A.K. Synthesis of Heteroleptic Terpyridyl Complexes of Fe(II) and Ru(II): Optical and Electrochemical Studies. *New J. Chem.* **2016**, *40*, 5775–5781, doi:10.1039/C5NJ03106K.

6. Li, L.; Du, K.; Wang, Y.; Jia, H.; Hou, X.; Chao, H.; Ji, L. Self-Activating Nuclease and Anticancer Activities of Copper(II) Complexes with Aryl-Modified 2,6-Di(Thiazol-2-Yl)Pyridine. *Dalton Trans.* **2013**, *42*, 11576–11588, doi:10.1039/C3DT50395J.

7. Li, J.; Yan, H.; Wang, Z.; Liu, R.; Luo, B.; Yang, D.; Chen, H.; Pan, L.; Ma, Z. Copper Chloride Complexes with Substituted 4′-Phenyl-Terpyridine Ligands: Synthesis, Characterization, Antiproliferative Activities and DNA Interactions. *Dalton Trans.* **2021**, *50*, 8243–8257, doi:10.1039/D0DT03989F.

8. Bera, S.; Basu, S.; Jana, B.; Dastidar, P. Real-Time Observation of Macroscopic Helical Morphologies under Optical Microscope: A Curious Case of π–π Stacking Driven Molecular Self-Assembly of an Organic Gelator Devoid of Hydrogen Bonding. *Angewandte Chemie International Edition* **2023**, *62*, e202216447, doi:10.1002/anie.202216447.

9. Zhang, Y.; Yu, W.-D.; Wang, F.-Q.; Wang, X.; Zhou, J.; Liu, C.; Yan, J. Four New Terpyridine Complexes Based Polyoxometalates with [W10O32]4– Anions as High-Efficiency Dual-Site Catalysis for Thioether Oxidation Reaction†. *Chinese Journal of Chemistry* **2024**, *42*, 592–598, doi:10.1002/cjoc.202300556.

10. Choroba, K.; Zowiślok, B.; Kula, S.; Machura, B.; Maroń, A.M.; Erfurt, K.; Marques, C.; Cordeiro, S.; Baptista, P.V.; Fernandes, A.R. Optimization of Antiproliferative Properties of Triimine Copper(II) Complexes. *J. Med. Chem.* **2024**, *67*, 19475–19502, doi:10.1021/acs.jmedchem.4c01806.

11. Maroń, A.; Czerwińska, K.; Machura, B.; Raposo, L.; Roma-Rodrigues, C.; Fernandes, A.R.; Małecki, J.G.; Szlapa-Kula, A.; Kula, S.; Krompiec, S. Spectroscopy, Electrochemistry and Antiproliferative Properties of Au(III), Pt(II) and Cu(II) Complexes Bearing Modified 2,2′:6′,2′′-Terpyridine Ligands. *Dalton Trans.* **2018**, *47*, 6444–6463, doi:10.1039/C8DT00558C.

12. Choroba, K.; Machura, B.; Kula, S.; Raposo, L.R.; Fernandes, A.R.; Kruszynski, R.; Erfurt, K.; Shul'pina, L.S.; Kozlov, Y.N.; Shul'pin, G.B. Copper(II) Complexes with 2,2′:6′,2′′-Terpyridine, 2,6-Di(Thiazol-2-Yl)Pyridine and 2,6-Di(Pyrazin-2-Yl)Pyridine Substituted with Quinolines. Synthesis, Structure, Antiproliferative Activity, and Catalytic Activity in the Oxidation of Alkanes and Alcohols with Peroxides. *Dalton Trans.* **2019**, *48*, 12656–12673, doi:10.1039/C9DT01922G.

13. Czerwińska, K.; Machura, B.; Kula, S.; Krompiec, S.; Erfurt, K.; Roma-Rodrigues, C.; Fernandes, A.R.; Shul'pina, L.S.; Ikonnikov, N.S.; Shul'pin, G.B. Copper(II) Complexes of Functionalized 2,2′:6′,2′′-Terpyridines and 2,6-Di(Thiazol-2-Yl)Pyridine: Structure,

Spectroscopy, Cytotoxicity and Catalytic Activity. *Dalton Trans.* **2017**, *46*, 9591–9604, doi:10.1039/C7DT01244F.

14. Patel, P.N.; Desai, D.H.; Patel, N.C. Synthesis, Spectral, and Single Crystal XRD Studies of Novel Terpyridine Derivatives of Benzofuran-2-Carbaldehyde and Their Cu(II) Complex. *Russ J Coord Chem* **2021**, *47*, 909–914, doi:10.1134/S1070328421120010.

15. Małecka, M.; Szlapa-Kula, A.; Maroń, A.M.; Ledwon, P.; Siwy, M.; Schab-Balcerzak, E.; Sulowska, K.; Maćkowski, S.; Erfurt, K.; Machura, B. Impact of the Anthryl Linking Mode on the Photophysics and Excited-State Dynamics of Re(I) Complexes [ReCl(CO)3(4′-An-Terpy-κ2N)]. *Inorg. Chem.* **2022**, *61*, 15070–15084, doi:10.1021/acs.inorgchem.2c02160.

16. Choroba, K.; Machura, B.; Raposo, L.R.; Małecki, J.G.; Kula, S.; Pająk, M.; Erfurt, K.; Maroń, A.M.; Fernandes, A.R. Platinum(II) Complexes Showing High Cytotoxicity toward A2780 Ovarian Carcinoma Cells. *Dalton Trans.* **2019**, *48*, 13081–13093, doi:10.1039/C9DT02894C.

17. Rojo, T.; Vlasse, M.; Beltran-Porter, D. The Structure of Dicholoro(2,2′:6′,2′′-Terpyridyl)Copper(II) Monohydrate, [Cu(C15H11N3)Cl2].H2O. *Acta Cryst C* **1983**, *39*, 194–199, doi:10.1107/S0108270183004096.

18. Henke, W.; Kremer, S.; Reinen, D. Copper(2+) in Five-Coordination: A Case of a Pseudo-Jahn-Teller Effect. 1. Structure and Spectroscopy of the Compounds Cu(Terpy)X2.nH2O. *Inorg. Chem.* **1983**, *22*, 2858–2863, doi:10.1021/ic00162a018.

19. Schmitt, L.; Stoeckli-Evans, H. CCDC 679674: Experimental Crystal Structure Determination 2008.

20. Manikandamathavan, V.M.; Rajapandian, V.; Freddy, A.J.; Weyhermüller, T.; Subramanian, V.; Nair, B.U. Effect of Coordinated Ligands on Antiproliferative Activity and DNA Cleavage Property of Three Mononuclear Cu(II)-Terpyridine Complexes. *European Journal of Medicinal Chemistry* **2012**, *57*, 449–458, doi:10.1016/j.ejmech.2012.06.039.

21. Paraskevopoulos, J.N.; Smith, P.J.; Hoppe, H.C.; Chopra, D.; Govender, T.; Kruger, H.G.; Maguire, G.E.M. Terpyridyl Complexes as Antimalarial Agents. *S.Afr.J.Chem.* **2013**, *66*, 00.

22. Rawji, G.; Fritz, C.; Nguyen, T.; Lynch, V. CCDC 952841: Experimental Crystal Structure Determination 2017.

23. Pal, P.; Das, K.; Hossain, A.; Frontera, A.; Mukhopadhyay, S. Supramolecular and Theoretical Perspectives of 2,2′:6′,2′′-Terpyridine Based Ni(II) and Cu(II) Complexes: On the Importance of C–H⋯Cl and Π⋯π Interactions. *New J. Chem.* **2020**, *44*, 7310–7318, doi:10.1039/D0NJ00094A.

24. Ma, Z.; Wei, L.; Alegria, E.C.B.A.; Martins, L.M.D.R.S.; Silva, M.F.C.G. da; Pombeiro, A.J.L. Synthesis and Characterization of Copper(II) 4′-Phenyl-Terpyridine Compounds and Catalytic Application for Aerobic Oxidation of Benzylic Alcohols. *Dalton Trans.* **2014**, *43*, 4048–4058, doi:10.1039/C3DT53054J.

25. Xiong, Kai; Zhou, Y.; Karges, J.; Du, K.; Shen, J.; Lin, M.; Kou, J.; Chen, Y.; Chao, H. CCDC 2078509: Experimental Crystal Structure Determination 2021.

26. Xiong, K.; Zhou, Y.; Karges, J.; Du, K.; Shen, J.; Lin, M.; Wei, F.; Kou, J.; Chen, Y.; Ji, L.; et al. Autophagy-Dependent Apoptosis Induced by Apoferritin–Cu(II) Nanoparticles in Multidrug-Resistant Colon Cancer Cells. *ACS Appl. Mater. Interfaces* **2021**, *13*, 38959–38968, doi:10.1021/acsami.1c07223.

27. Beves, J.E.; Constable, E.C.; Decurtins, S.; Dunphy, E.L.; Housecroft, C.E.; Keene, T.D.; Neuburger, M.; Schaffner, S.; Zampese, J.A. Structural Diversity in the Reactions of 4′-(Pyridyl)-2,2′:6′,2″-Terpyridine Ligands and Bis{4′-(4-Pyridyl)-2,2′:6′,2″-Terpyridine}iron(II) with Copper(II) Salts. *CrystEngComm* **2009**, *11*, 2406–2416, doi:10.1039/B909639F.

28. Khavasi, H.R.; Esmaeili, M. Case Study of the Correlation between Metallogelation Ability and Crystal Packing. *Crystal Growth & Design* **2019**, *19*, 4369–4377, doi:10.1021/acs.cgd.9b00117.

29. Karges, J.; Xiong, K.; Blacque, O.; Chao, H.; Gasser, G. Highly Cytotoxic Copper(II) Terpyridine Complexes as Anticancer Drug Candidates. *Inorganica Chimica Acta* **2021**, *516*, 120137, doi:10.1016/j.ica.2020.120137.

30. Bhowmik, S.; Ghosh, B.N.; Rissanen, K. Transition Metal Ion Induced Hydrogelation by Amino-Terpyridine Ligands. *Org. Biomol. Chem.* **2014**, *12*, 8836–8839, doi:10.1039/C4OB01867B.

31. Patel, P.N.; Rajalakshmi, S.; Chadha, A. CCDC 1525024: Experimental Crystal Structure Determination 2020.
